# An unusually high substitution rate in transplant-associated BK polyomavirus *in vivo* is further concentrated in HLA-C-bound viral peptides

**DOI:** 10.1371/journal.ppat.1007368

**Published:** 2018-10-18

**Authors:** Pilar Domingo-Calap, Benjamin Schubert, Mélanie Joly, Morgane Solis, Meiggie Untrau, Raphael Carapito, Philippe Georgel, Sophie Caillard, Samira Fafi-Kremer, Nicodème Paul, Oliver Kohlbacher, Fernando González-Candelas, Seiamak Bahram

**Affiliations:** 1 Plateforme GENOMAX, Laboratoire d’ImmunoRhumatologie Moléculaire, INSERM UMR_S1109, LabEx Transplantex, Centre de Recherche d’Immunologie et d’Hématologie, Faculté de Médecine, Fédération de Médecine Translationnelle de Strasbourg (FMTS), Université de Strasbourg, Strasbourg, France; 2 Fédération Hospitalo-Universitaire, OMICARE, Centre de Recherche d’Immunologie et d’Hématologie, Strasbourg, France; 3 Center for Bioinformatics, University of Tübingen, Tübingen, Germany; 4 Applied Bioinformatics, Department of Computer Science, Tübingen, Germany; 5 Service de Néphrologie et Transplantation Rénale, Hôpitaux Universitaires de Strasbourg, France; 6 Laboratoire de Virologie, Plateau Technique de Microbiologie, Pôle de Biologie, Hôpitaux Universitaires de Strasbourg, France; 7 Laboratoire Central d’Immunologie, Plateau Technique de Biologie, Nouvel Hôpital Civil, France; 8 Quantitative Biology Center, Tübingen, Germany; 9 Faculty of Medicine, University of Tübingen, Tübingen, Germany; 10 Biomolecular Interactions, Max Planck Institute for Developmental Biology, Tübingen, Germany; 11 Institute for Translational Bioinformatics, University Hospital Tübingen, Tübingen, Germany; 12 Unidad Mixta Infección y Salud Pública FISABIO/Universitat de València, Institute for Integrative Systems Biology I2SysBio (CSIC-UV) and CIBER en Epidemiología y Salud Pública, Valencia, Spain; Institut Pasteur, FRANCE

## Abstract

Infection with human BK polyomavirus, a small double-stranded DNA virus, potentially results in severe complications in immunocompromised patients. Here, we describe the *in vivo* variability and evolution of the BK polyomavirus by deep sequencing. Our data reveal the highest genomic evolutionary rate described in double-stranded DNA viruses, i.e., 10^−3^–10^−5^ substitutions per nucleotide site per year. High mutation rates in viruses allow their escape from immune surveillance and adaptation to new hosts. By combining mutational landscapes across viral genomes with *in silico* prediction of viral peptides, we demonstrate the presence of significantly more coding substitutions within predicted cognate HLA-C-bound viral peptides than outside. This finding suggests a role for HLA-C in antiviral immunity, perhaps through the action of killer cell immunoglobulin-like receptors. The present study provides a comprehensive view of viral evolution and immune escape in a DNA virus.

## Author summary

Little is known about the mechanisms of evolution and viral immune escape in double-stranded DNA (dsDNA) viruses. Here, we study the evolution of BK polyomavirus and observe the highest genomic evolutionary rate described so far for a dsDNA virus, in the range of RNA viruses, which usually evolve rapidly. Furthermore, the prediction of viral peptides to determine immune escape suggests a specific role of HLA-C in antiviral immunity. These findings are helpful for future advances in antiviral therapies and provide a step forward in our understanding of *in vivo* viral evolution in humans.

## Introduction

Viral evolutionary rates can vary strongly depending on the method used to estimate them [1, 2]. Among Baltimore groups, the fastest evolving entities are single-stranded (ss) RNA and reverse-transcribing (RT) viruses, with rates ranging between 10^−2^ and 10^−5^ substitutions per site per year (s/s/y). The rates of double-stranded (ds) RNA and ssDNA viruses range between 10^−3^ and 10^−6^ s/s/y, whereas dsDNA viruses evolve more slowly (10^−3^ and 10^−8^ s/s/y) [3, 4]. It is important to note that only few estimates on dsDNA viruses are published. In fact, higher estimates are based on specific genes, as estimated for human papillomavirus 16 (E6 and E7), human adenovirus (hexon), or JC virus (VP1), which are in the order of 10^−3^ s/s/y [4, 5]. Regarding estimates based on dsDNA complete genomes, all of them range between 10^−5^ and 10^−7^ s/s/y [3, 5]. This finding confirms that viruses are fast evolving entities whereas humans have much lower evolutionary rates (10^−8^–10^−9^ s/s/y). However, the well-established co-divergence of viral populations with their hosts suggests the possibility of low evolutionary rates in viruses as well. For example, polyomaviruses were historically considered to be examples of human-virus co-divergence, and have been used as markers for human migration patterns, with proposed estimates ranging from 1.41 × 10^−7^ to 4 × 10^−8^ s/s/y [6, 7]. Detailed studies are needed to better understand dsDNA virus evolution *in vivo*, especially in viruses that can be considered as potential pathogens.

In vertebrates, the major driving force in anti-viral immunity is the high level of polymorphism in human leukocyte antigen (HLA) genes. Despite a few recent reports [8, 9], limited information is presently available on the extent of viral variability *in vivo*, especially at the whole viral genome level, and only a few studies have tackled this variability in conjunction with the HLA genotype of infected individuals. Consequently, viral escape mutants—i.e., viruses that produce mutated peptides that are no longer able to bind to cognate HLA molecules—have been mainly studied for limited model epitopes in *in vitro* systems and in highly relevant RNA viruses such as HIV, HCV, influenza or dengue (see the following historical references [10, 11]; for a recent review and full bibliography on the subject see [12]). It is not surprising that RNA viruses can adapt to circumvent the immune responses [4], but little is known about viral escape in DNA viruses.

A better understanding of the epitopes involved in viral escape from the immune system could be useful for the development of vaccines and specific treatments. Here, we initiate a dual approach using the BK virus (BKV) as a model. BKV, which was detected for the first time in 1971, is a 5.1 kb dsDNA virus of the *Polyomaviridae* family that harbors six genes (Agnogene, VP1 to VP3, large T antigen “LTA” and small t antigen “stA”) [13]. The primary infection occurs essentially in childhood and the virus infects up to 90% of the human population. The virus remains persistent throughout life, primarily in the urinary tract [14]. High-level replication mainly occurs in immunocompromised hosts and, more specifically in those receiving modern immunosuppressive regimens, notably post-kidney transplantation. BKV-associated diseases, especially BKV-associated nephropathy, affect 1–10% of transplant recipients [15, 16] and may lead to loss of the allograft and even death [17]. There are no specific prophylactic or curative treatments, and early diagnosis, as well as quick restoration of immunity (through dampening of immunosuppression), remain the most effective strategies to control the disease.

## Results and discussion

### High level of variability in BKV as detected by NGS

**Fig 1:**
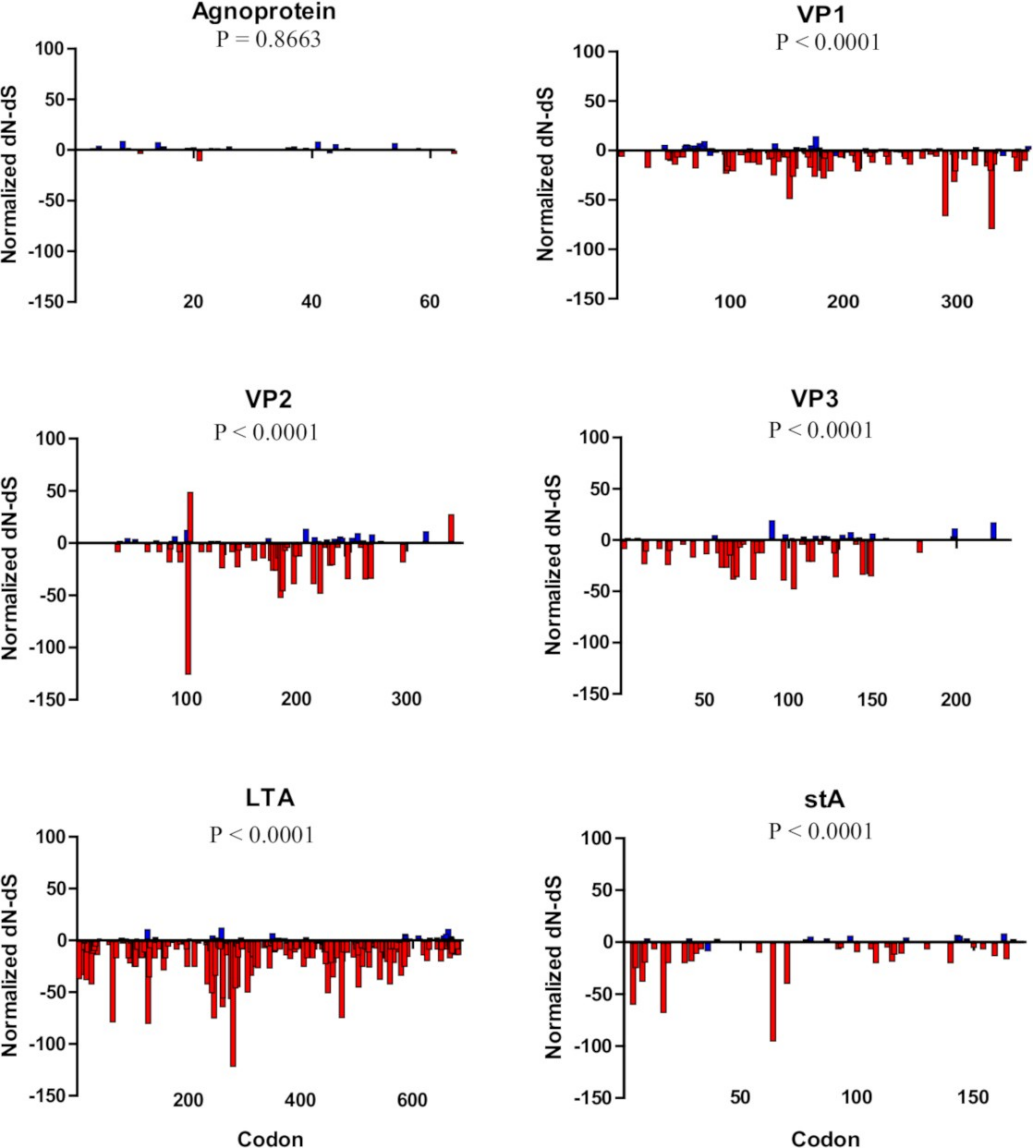
Distribution of the normalized dN-dS per codon among proteins. The six proteins are represented (Agnoprotein, VP1 to VP3, large T antigen “LTA” and small t antigen “stA”). Non-significant values are shown in blue, and significant values in red (positive values for positive selection and negative values for purifying selection, two-tailed binomial distribution). *P-*values correspond to the Nei-Gojobori test of neutrality for each gene.

Access to the virus in the bloodstream and/or urine within a transplant setting, where HLA alleles of both donors and recipients are known, provides a unique opportunity to study viral evolution *in vivo* in the context of the individual’s (both recipient and donor) HLA class I genotype. A retrospective cohort of 96 patients—225 samples—that underwent solid organ (N = 83) or hematopoietic cell transplantations (N = 13), harboring a minimum of 10^4^ viral copies/mL in whole blood or urine, was selected. Quantitative real-time PCR showed that the viral titers in blood (8.98 × 10^4^ ± 2.47 × 10^4^ copies/mL) were significantly lower than those in urine (2.16 ×10^9^ ± 3.94 × 10^8^ copies/mL) (Mann-Whitney U = 315.0, two-tailed, *P* < 0.0001). After complete deep genome sequencing of all 225 samples and alignment to the BKV Dunlop reference strain (GenBank accession number NC001538), an average of 110 ± 3 polymorphisms per sample was observed with an average median coverage of 3043 ± 78 reads/position (S1 Table, GenBank accession numbers KT896230-KT896454; see Methods). In total, 37.88% of all amino acid positions in the Agnoprotein, 12.43% in VP1, 9.97% in VP2, 11.21% in VP3, 8.20% in LTA and 8.72% in stA, were found to be polymorphic (S2 Table). *Agnogene* is the only gene that is not under apparent selective constraints (Nei-Gojobori test, *P* = 0.8663), while the others are under purifying selection (Nei-Gojobori test, *P* < 0.0001, Fig 1, see Methods). Only a few single nucleotide insertions or deletions were detected in the viral genes (S3 Table). Due to methodological limitations (short reads) the non-coding control region was not included in the analyses.

The occurrence of mutations is the main process generating genetic variability, but other processes, such as genetic drift, gene flow, selection and recombination, are responsible for shaping the genetic structure and variation of viral populations. Here, we present evidence that BKV is under strong purifying selection even in the immunocompromised host. Several specific features of the *Polyomaviridae* (e.g., limited size of the genome, small number of genes and overlapping transcription units) likely account for this outcome. In addition, the prevalence of purifying selection in essential genes is anticipated in all viruses as there is a requirement to complete the viral cycle, even in immunocompromised hosts. Most mutations in coding regions must be deleterious, and a high substitution rate implies the accumulation of mutations with deleterious effects [18]. This phenomenon is well known in RNA viruses, which have high mutation rates and short replication times. Similar results have been shown comparing mutational fitness effects and evolution in ssRNA and ssDNA viruses [19, 20]. Our study supports the hypothesis, in concordance with other recent findings [21], that the evolutionary rate gap between small dsDNA and RNA viruses might not be as wide as previously thought. A recent study in lentiviruses has revealed that the combined effects of sequence saturation and purifying selection can explain the time-dependent pattern of rate variation. Purifying selection acts on the genetic diversity over long timeframes by removing a large number of transient deleterious mutations that are still present within short timeframes [4].

### Phylogenetic analysis: Incongruent results between serology and genotyping

**Fig 2:**
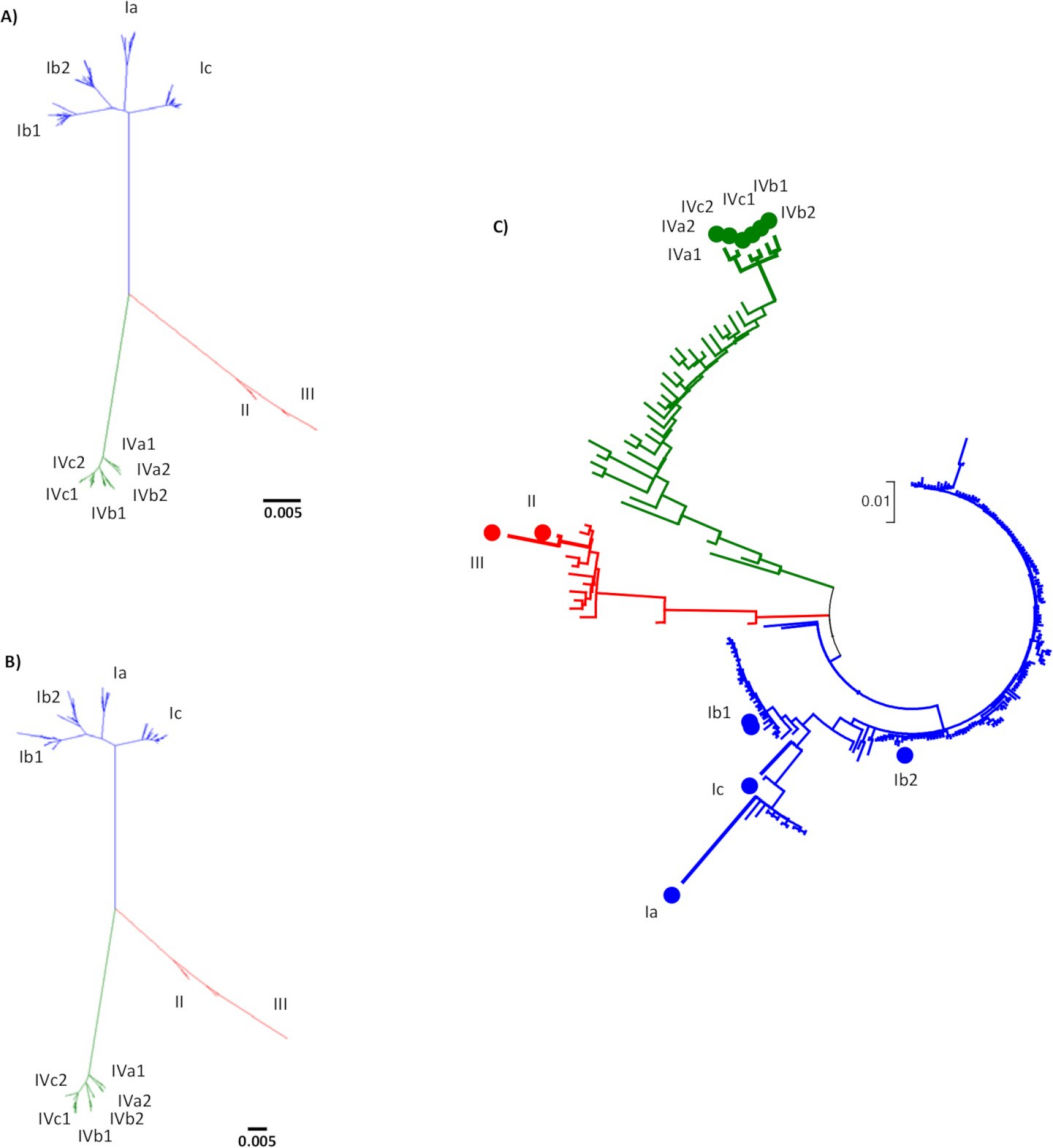
Maximum likelihood phylogenetic trees of BK polyomavirus. Three major groups are found: genotype I in blue, a single group including genotypes II/III in red, and genotype IV in green. (A) Unrooted ML phylogenetic tree with 309 complete genome published sequences retrieved from NCBI. (B) Unrooted ML phylogenetic tree with 309 VP1 gene sequences retrieved from NCBI. (C) Unrooted ML phylogenetic tree with 225 complete genome consensus sequences obtained in this study by next-generation sequencing and one reference strain of each genotype and subtype. Reference strains are marked with dots (Ia, Ib1, Ib2, Ic, II, III, IVa1, IVa2, IVb1, IVb2, IVc1, IVc2).

Phylogenetic analysis with all BKV complete genomes available from GenBank (Fig 2A) suggested the existence of three large groups or genotypes represented by serotypes I, II/III, and IV, with subtypes within genotypes. Limited differences (short branch lengths) between the previously designated genotypes II and III suggested the existence of only one genotype II/III with two subtypes (in contrast to more pronounced differences between serotypes II and III). A similar phylogenetic classification was observed by analyzing only the VP1 gene (Fig 2B). Incidentally, this finding indicated that the current BKV classification should be revised due to inconsistencies between serotyping and genotyping. Next, to establish the genotype of our samples, one reference strain of each genotype and subtype was used for the phylogenetic analysis (Fig 2C). Most of our samples (80.88%) belonged to genotype I, whereas genotypes IV and II/III were less represented (13.78% and 5.3% respectively). The clustering was patient-dependent but independent of the sample origin (urine or blood) and suggested that some samples likely contained a mixture of genotypes. This mixture might be due to multiple lifelong infections or the replication of viruses from the recipient and/or the donor.

### High intra- and inter-patient evolutionary rates in BKV

Intra- and inter-patient evolutionary rates were estimated. BKV sequences from samples with possible recombination or a mixture of genotypes according to the RDP output [22] were removed from the analysis (see Methods). We estimated an intra-patient substitution rate for BKV in transplanted patients in the range of 4.90 × 10^−4^–1.22 × 10^−3^ substitutions per nucleotide site per year (s/s/y). No differences between substitution rates in solid organ and hematopoietic cell transplant recipients were found (t-test, P = 0.2581). To estimate the inter-patient evolutionary rate, the best substitution (molecular clock) and demographic model according to marginal likelihood analyses was the relaxed log-normal uncorrelated clock with Bayesian skyline demographic prior. The estimated inter-patient evolutionary rate ranged from 1.00 × 10^−5^–2.15 × 10^−4^ (95% HDI) for a maximum sampling interval of 568 days. The estimate was quite robust to different demographic and molecular clock models (S4 Table).

The evolutionary rates based on the maximum likelihood and least-squares methods implemented in *treedater* were similar when applied to the whole data set (4.30 × 10^−3^ s/s/y) but with large parametric bootstrap confidence intervals (in the 10^−20^ to 10^14^ range), thus preventing their consideration as reasonable estimates. However, when the dataset was reduced to the sequences of genotype I (n = 56) the average evolutionary rate was estimated at 1.33 × 10^−4^ (95% CI = 3.13 ×10^−6^–5.59 × 10^−3^). These values were close to those obtained with the Bayesian approach described previously.

It is usually assumed that RNA viruses evolve at a rate of 10^−4^ s/s/y, while dsDNA can be close to 10^−8^ s/s/y [23]. ssDNA viruses with small genomes evolving at rates similar to those of RNA viruses have been reported previously [24, 25], as illustrated by the canine parvovirus, with a substitution rate of 1.7 × 10^−4^ s/s/y [26]. In the case of dsDNA, many evolutionary rates have been calculated under the assumption of co-divergence between viral and human populations, as observed for polyomaviruses. Recently, the substitution rate for JC polyomavirus was evaluated at 1.7 × 10^−5^ s/s/y [27]. Based on this result, Bayesian analyses suggested the substitution rate of BKV to be on the order of 10^−5^ s/s/y [5, 28], while another study found only minor nucleotide substitutions in the genes encoding late proteins [29].

**Fig 3:**
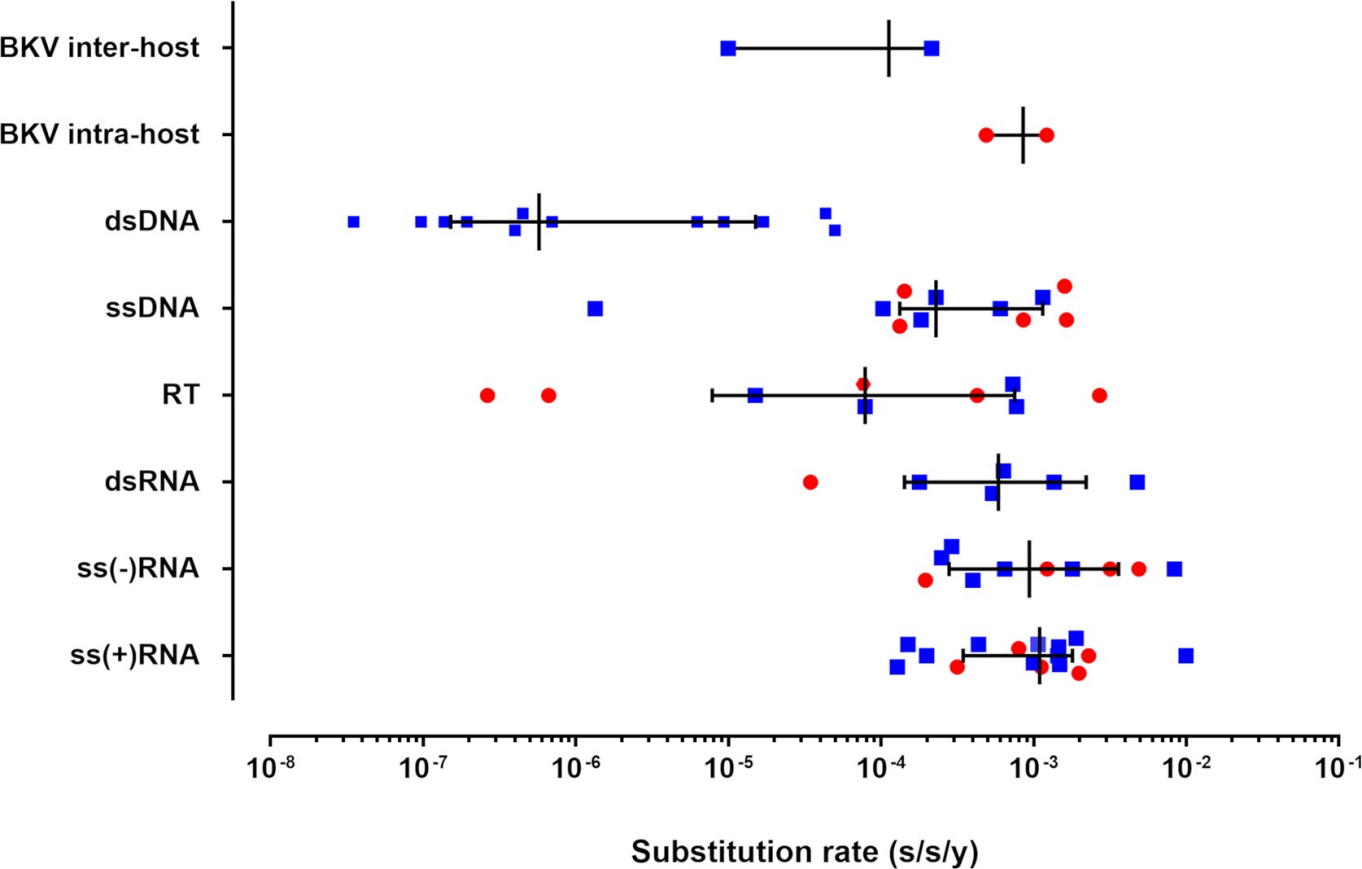
Genomic evolutionary rates for the major Baltimore groups and BKV. Substitution rates are given as substitutions per nucleotide site per year (s/s/y). For the major groups (***dsDNA***: double-stranded DNA viruses—BKV [5, 7, 28] (time span of sequences (TSS) of 29 years (y), 25 y, and 32 y, respectively), JC polyomavirus [27, 31] (TSS 33 y and 13 y, respectively), herpes simplex virus 1 [32, 33] (TSS not available and 21 y, respectively), human papillomavirus 18 [34] (TSS not available), monkeypox virus [35] (TSS 7 y), variola virus [5] (TSS 31 y), varicella zoster virus [5] (TSS 37 y); ***ssDNA***: single-stranded DNA viruses—African cassava mosaic virus [25] (TSS 5 y), banana bunchy top virus [36] (TSS 2 months), human bocavirus [37] (TSS 1 y), human parvovirus B19 [38, 39] (TSS 14 y and 28 y, respectively), porcine circovirus 2 [40] (TSS 27 y), tomato yellow leaf curl virus [41] (TSS 29 y); ***RT***: retroviruses—avian hepatitis B virus [42] (TSS 22 y), human hepatitis B virus [42–44] (TSS 22 y, 25 y and 35 y, respectively); human immunodeficiency virus 1 [45] (TSS 2 y), primate T-cell lymphotropic virus [45] (TSS 2 y); ***dsRNA***: double-stranded RNA viruses—bluetongue virus [46] (TSS 48 y), human rotavirus [47] (TSS 16 y), homalodisca vitripennis virus [48] (TSS 2 y); ***ss(-)RNA***: single-stranded RNA viruses with negative polarity–Ebola virus [49] (TSS 4 months), fever, thrombocytopenia and leukocytopenia syndrome virus [50] (TSS 4 y), influenza A virus [51, 52] (TSS 28 y and 1 y, respectively), hepatitis delta virus [53] (TSS 3 y), human respiratory syncytial virus [54] (TSS 10 y), rabies virus [55] (TSS 30 y), rift valley fever virus [56] (TSS 10 y); and ***ss(+)RNA***: single-stranded RNA viruses with positive polarity—avian coronavirus [57] (TSS 41 y), barley yellow dwarf virus [58] (TSS 2 y), dengue virus [59](TSS 29 y), foot-and-mouth disease virus [60] (TSS 75 y), hepatitis A virus [61] (TSS 13 y), hepatitis C virus [62] (TSS 20 y), Japanese encephalitis virus [63] (TSS 60 y), Middle East respiratory syndrome coronavirus [64](TSS 4 months), porcine reproductive and respiratory syndrome virus [65] (TSS 3 y), rubella virus [66] (TSS not available), severe acute respiratory syndrome coronavirus [67] (TSS 4 months), St. Louis encephalitis virus [68] (TSS 46 y), Venezuelan equine encephalitis virus [69] (TSS 54 y)). Each point represents the value of a previously published genomic evolutionary rate (note that for some references, more than one substitution rate is represented in the caption). Red circles represent short time span estimates (< 5 years) and blue squares represent long-time span estimates (> 5 years). Medians with interquartile ranges are indicated. In the case of the inter- and intra-host genomic evolutionary rates of BKV, the values are represented as a range of values obtained in this study.

Here we estimated a substitution rate for BKV on the order of 10^−3^–10^−5^ s/s/y (Fig 3). Our experimental results show, for the first time using whole-genome sequencing of *in vivo* viral populations (in a large monocentric cohort), that the genomic evolutionary rate of a dsDNA virus can be as high as that of RNA viruses. It is important to note that the sampling window of sequences may affect the estimates of evolutionary rates, because very short timescales can inflate them. A recent study has shown that estimates of evolutionary rates were lower for broader sampling levels and longer timeframes for both, DNA and RNA viruses, suggesting that the time dependence of substitution rates is ubiquitous among all viruses [4]. For example, lentivirus evolutionary rates from serial samples over a few years within a single patient or host are in the order of 10^−3^ s/s/y [30], reflecting those observed in this study in a small dsDNA virus.

In addition, a previous study comparing the evolution of ssRNA and ssDNA viruses has shown that small genomes (< 5 kb) can evolve rapidly [24] regardless of their encoding material, and that the well-known correlation between genome size and mutation rate [70] can also hold for evolutionary rates. Here, we show that small dsDNA genomes can also evolve as fast as single-stranded ones. Although BKV uses the host DNA polymerase for its replication, the virally-encoded Agnoprotein inhibits dsDNA break repair activity, thereby potentially increasing the error rate during BKV DNA replication [71]. Interestingly, cell tropism of RNA viruses was recently suggested as a key factor in their capacity to evolve, since viruses replicating in epithelial cells (as BKV) are characterized by rapid replication and higher substitution rates [72].

To investigate the relationship between the evolutionary rate of the virus and the immunosuppressive drug regimen—hence the strength of the immune system—we analyzed such information in our kidney transplant recipient cohort (the largest subgroup in our cohort). Kidney transplant patients were given either anti-thymocyte globulin (ATG) (immunological high-risk patients) or anti-Interleukin-2 receptor (anti-IL-2R) (immunological low-risk patients) as induction treatments, and tacrolimus (immunological high-risk patients) or cyclosporine (immunological low-risk patients) as maintenance therapy. Mycophenolate mofetil and steroids were also part of both drug regimens (for high- and low-risk patients). Evolutionary analysis of the different subgroups showed no significant differences in the mutational load (full negative binomial mixed model regression with random effect intercept to account for repeated measures) nor in inter-patient substitution rates where ranges overlapped between treatments (ATG 6.12 × 10−4–1.03 × 10^−5^ s/s/y, Anti-IL-2R 8.60 × 10^−4^–1.36 × 10^−5^ s/s/y, tacrolimus 4.64 × 10^−4^–9.31 × 10^−6^ s/s/y, and cyclosporine 1.72 × 10^−3^–1.11 × 10^−5^ s/s/y).

### Immune escape in BKV associated with HLA-C epitopes

To investigate the genetic immune escape mechanism of BKV, potential T-cell epitopes presented by HLA class I were predicted using both donor and recipient HLA alleles, combined with the viral substitutions found herein (S1 Fig, S2, S5 and S6 Tables, see Methods). In this way, we determined the putative HLA ligandome of the virus as linked to the individual’s cognate HLA genotype. Interestingly, the two codons in VP2 that appeared to be under positive selection corresponded to codons within predicted epitopes. The VP2 103 codon, the one with the highest level of significant difference, was found in three predicted HLA-C epitopes (KFFDDWDHKVSTV, FFDDWDHKV and FFDDWDHKVSTV), and codon 340 was located within two HLA-A predicted epitopes (TTNKRRSR and TTNKRRSRSSR).

**Fig 4:**
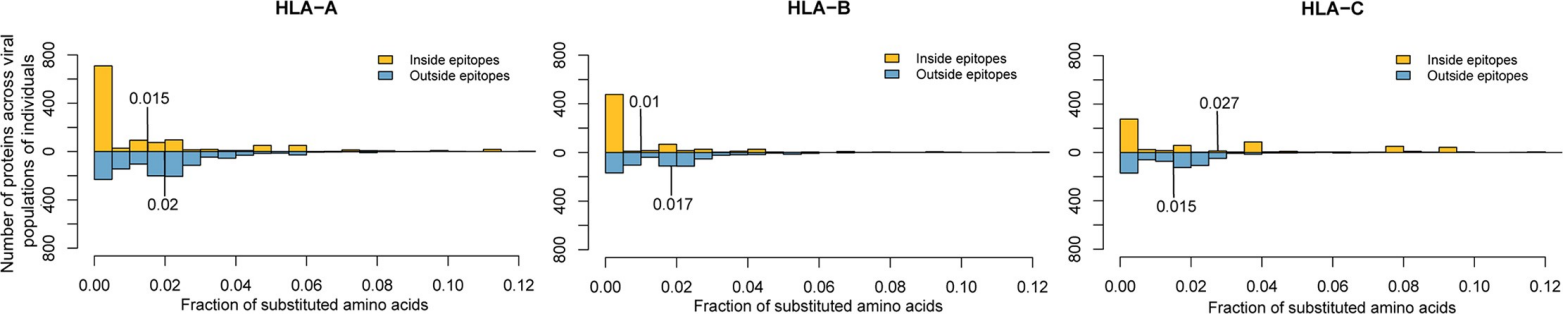
**Fraction of amino acid substitutions within and outside of predicted epitopes presented by HLA-A, -B and -C molecules across individuals**. The detected amino acid substitutions of a viral population were mapped onto reference proteins and the fraction of mutated amino acids within and outside of predicted epitopes of each viral protein and hosts HLA allele were calculated for each viral population found in patient and donor respectively. The fraction of substituted amino acids within HLA-A and -B presented epitopes (yellow) is significantly lower compared with the fraction outside (blue), while the fraction of amino acid substitutions in HLA-C binding epitopes is significantly higher compared with the fraction outside.

We also found a higher fraction of observed amino acid substitutions within HLA-C epitopes compared with the fraction of amino acid substitutions outside of HLA-C epitopes (one-sided Wilcoxon signed test, *P* = 3.71 × 10^−10^). The opposite behavior was observed for HLA-A and -B presented epitopes (one-sided Wilcoxon signed, HLA-A: *P* = 4.17 × 10^−29^; HLA-B: *P* = 1.35 × 10^−26^) (Fig 4). This difference in contribution of HLA loci was independent of the transplantation type (solid organ or hematopoietic) or the origin of the HLA loci (whether from the donor or the recipient) as assessed by a three-way ANOVA (*P* = 0.7947). Therefore, our results suggest that HLA-C might be specifically involved in the immune response against BKV through its peptide selection capacity for viral peptides. A possible mechanistic explanation for this finding stems from the amply documented interaction of HLA-C with natural killer (NK) and T cells expressing the killer cell immunoglobulin-like receptors (KIR). Notably, the relevance of KIR and HLA-C interactions has been described for viral infections [73, 74], and the involvement of NK cells in the immune response against BKV has also been reported [75, 76], although further investigations should be done to confirm this hypothesis.

High evolutionary rates in RNA viruses allow them to escape immune pressures. Interactions between HLA epitopes and viruses have been described for a variety of RNA viruses, such as HIV, HCV, influenza or dengue, while little is known about immune escape in DNA viruses. A few studies in HPV-16 or herpes simplex virus have been done to improve vaccine design and drug development, but those studies have only examined a fraction of the proteins and not at whole-genome sequencing data [77–79]. This work, to our knowledge, is the first in which predicted epitopes from whole genome sequencing have been studied in an *in vivo* cohort, in conjunction with cognate HLA alleles, to understand the mechanism involved in immune escape in a DNA virus. Our results of viral escape combined with the high evolutionary rate described herein suggest that a combination of drugs should be used as potential treatment against BKV, as commonly used in highly variable viruses such as HIV and HCV, due to the variable viral populations present in a single patient as observed in our study.

### Conclusions

The present work describes an unusually fast evolutionary rate for BKV *in vivo* and charts its interaction with the immune system—through the analysis of cognate HLA alleles—whilst considering the whole viral genome and not only candidate epitopes. It further offers a blueprint for similar analyses in other viruses and helps to better rationalize anti-viral therapy and candidate vaccine development. Our results suggest that small dsDNA viruses should be treated as RNA viruses due to their similarities in evolution and immune escape. Thus, a combination of drugs might be necessary for the treatment of BKV, as used for fast evolving RNA viruses. It is important to note that new analytic methods for the study of the evolutionary rates are needed to better understand the effect of time spans and improve the comparison between estimates.

## Materials and methods

### Patients and samples

Ninety-six transplanted patients between 2012 and 2013 from the Strasbourg University Hospitals (France) with high levels of post-transplant BKV viruria—as detected by routine BKV testing at the hospital’s clinical virology laboratory—were enrolled in this study. Sixty-eight patients underwent kidney transplantation, 12 were lung recipients, 3 received double (kidney-heart; heart-lung or kidney-pancreas) transplants and 13 hematopoietic stem cell transplantation. A total of 225 samples, including 197 urine (from 94 patients) and 28 whole blood (from 13 patients) were included. Urine samples were collected longitudinally for 36 patients.

### Ethics statement

All patients were enrolled in the study following the Helsinki guidelines. Written informed consent for genetic testing was obtained from all patients and the study was approved by the Strasbourg University Hospitals institutional review board (RNI DC-2013-1990).

### DNA isolation, quantitative BKV real-time PCR, PCR and sanger sequencing

Urine and whole blood samples were collected, and DNA was purified using the QIAxtractor instrument (Qiagen, Hilden, Germany), following the DX protocol. Extracted DNA was stored at -80°C until analysis. Blood and urine specimens were assessed using the BK virus R-gene quantification kit (Biomérieux, Lyon, France) following the manufacturer’s recommendations. DNA was amplified by Phusion Polymerase (New England Biolabs, MA, USA) using specific overlapping primers. Nested PCR was performed for samples with a low BKV DNA load (usually blood samples). PCR products were purified using the GeneJET DNA purification Kit (ThermoFisher Scientific, Waltham, MA, USA) and quantified with Qubit (ThermoFisher Scientific, Waltham, MA, USA). Twenty-one urine-blood paired samples were used for sequencing by the Sanger method using an ABI Prism 3130 Genetic Analyzer (ThermoFisher Scientific, Waltham, MA, USA). Bi-directional sequencing was performed with the Big Dye Terminator v3.1 kit (ThermoFisher Scientific, Waltham, MA, USA) following the manufacturer’s recommendations. Chromatograms were analyzed with the Staden package (24) to obtain the consensus sequence for each sample. These consensuses were obtained to compare with the results after the next-generation sequencing assembly to validate our pipeline.

### Next-generation sequencing (NGS) and sequence assembly

All 225 urine and blood samples were sequenced by NGS. PCR products from the same samples were pooled in equimolar amounts and library construction with barcodes was performed according to the Fragment Library Preparation protocol using the AB Library Builder System (ThermoFisher Scientific, Waltham, MA, USA). Libraries were quantified by Qubit (ThermoFisher Scientific, Waltham, MA, USA) and then pooled in equimolar amounts for Template beads preparation using the SOLiD EZ beads System (ThermoFisher Scientific, Waltham, MA, USA). Template beads were subjected to sequencing using SOLiD 5500 (ThermoFisher Scientific, Waltham, MA, USA) with the paired-end 75 bp / 35 bp workflow. Sequences were assembled against the Dunlop reference strain (GenBank accession number NC001538) using LifeScope software (ThermoFisher Scientific, Waltham, MA, USA). Comparison with Sanger sequencing was performed to ascertain the correct assemblies. To quantify the variability per sample, mutations were analyzed with SeqMan software (DNASTAR, Madison, Wisconsin, USA). For each sample, we obtained a list of variants with their genomic location, coverage, and quality metrics, among others. To establish a cutoff for variant calling, we introduced internal controls including (a) a clone from the Dunlop reference strain, pBK (BKV34-2) plasmid (ATCC 45025) prepared by minipreparation (ThermoFisher Scientific, Waltham, MA, USA); (b) PCR amplicons from the same clone; and (c) PCR amplicons in duplicate from three of the samples. These controls were processed using the same sequencing methodology to establish the rate of sequencing and PCR errors. The final list of variants was selected by means of a Fisher's exact one-sided test comparing evidence obtained from the data for every potential polymorphism to the estimated error rate using our internal controls. Based on this analysis, BKV sequence variants found in less than 0.5% of reads were removed from the analysis.

### Mutation analysis

Sequences were aligned and assembled against the Dunlop strain by Muscle implemented in MEGA version 6 [80] with default parameters in order to compare and determine point mutations, insertions, deletions, and other sequence variations. For better analysis of coding regions, individual datasets per gene were obtained. Further analysis of synonymous and non-synonymous substitutions and the Nei-Gojobori test of neutrality were performed with MEGA version 6 [80].

### Phylogenetic analyses

Phylogenetic analyses of the whole genome consensus sequences obtained from all samples, and for each gene separately, were performed using MEGA version 6 [80]. Maximum likelihood phylogenetic trees were constructed with the general time reversible model (GTR) of nucleotide substitution with gamma distribution to account for rate heterogeneity among sites, as this model achieved the lowest AIC score. Similar analyses were performed for 309 BKV complete genome sequences collected from GenBank (all items found by searching the NCBI nucleotide database for “BK polyomavirus complete genome”).

To genotype the populations in the different samples, two approaches were performed. First, phylogenetic trees with all our samples and one of the reference strains for each genotype and subtype were obtained following the methodology explained previously. We determined the genotype as the shortest branch distance to one reference. The second approach was based on the methodology proposed by Luo and colleagues, in which point mutations specifically reported in particular genotypes are described [81].

### Estimation of substitution rates

To estimate the evolutionary rates of BKV, intra- and inter-patient analyses were performed. Upon multiple alignment, consensus sequences were tested using RDP software [22] for potential recombination, and those with positive results using at least two different methods implemented in the RDP package were removed from the ensuing analyses. Samples showing mixtures of genotypes were also excluded since they could interfere with the calculation of the substitution rate. To estimate the intra-patient substitution rate, we used urine samples from twenty-five patients collected at different times (the first positive samples and after 6 months). To calculate substitutions per site per year, we considered all the different genomic positions between two different times that were fixed in the populations. All the substitutions that reverted to the reference base were not included since the possibility of them already being present in the ancestral population at a low frequency could not be ruled out. Thereby only substitutions appearing *de novo* and exhibiting a high proportion in the population (fixed substitutions, more than 80% of the reads) were included in this approach. With this methodology, we obtained conservative estimates.

To estimate the inter-patient substitution rate, the consensus sequence for the first available urine sample of each patient with a known date of sampling was selected. After being tested by RDP, a dataset of 79 BKV sequences was used to estimate the inter-patient evolutionary rate (sequences from 15 patients were potential recombinants). A maximum likelihood phylogenetic tree was obtained using Phyml [82] with the GTR model with gamma distribution and invariant sites to account for heterogeneity among sites. This model was determined to be the most appropriate for this dataset with jModeltest [83]. TempEst analysis was conducted to detect a correlation between genetic divergence and sampling time, and it assured a temporal signal in our inter-patient dataset (S2 Fig) [84]. We used Bayesian estimates of the evolutionary rate with dated tips as implemented in BEAST [85]. Based on previous results by Firth et al. [5], we considered three molecular clock models (strict, relaxed log-normal uncorrelated, and relaxed exponential uncorrelated) and two demographic models (constant population size and Bayesian skyline). The GTR model with a gamma distribution and invariant sites was used as the nucleotide substitution model in all combinations. Model selection was performed through computation of the marginal likelihood using path sampling and stepping stone sampling analyses [86]. A lognormalPrior with a mean of 1 × 10^−6^ and a standard deviation of 1.0 was used for the substitution rate. Two independent runs of 30 million steps with 10% burn-in were used to obtain the median and 95% high probability density intervals for the relevant parameters in each model. In all cases, the effective sample size was > 200, as checked with Tracer v. 1.5 (available from http://beast.bio.ed.ac.uk).

In addition, we used the recently developed method of Volz and Frost which uses maximum likelihood and least squares to estimate evolutionary rates and dates based on relaxed molecular clocks. The method is implemented in the R package *treedater* [87].

### Prediction of BKV epitopes

To predict BKV-encoded T-cell epitopes that can be presented by HLA alleles, HLA high-resolution typing (2 fields) was done at the Etablissement Français du Sang Grand Est (Strasbourg) using a sequence-specific oligonucleotide technology. High-resolution typing data of *HLA-A*, *-B* and *-C* of 75 available donor / recipient pairs were used in each analysis, using the recipient’s viral populations in each case (S5 Table).

NetMHC 3.4 [88] was used to predict the peptide binding affinities of potential HLA class I epitopes occurring in BKV Dunlop reference proteins to HLA class I alleles of the patients and donors. Peptides eliciting a predicted IC_50_ of less than 50 nM were considered epitopes. IC_50_ values represent the concentration of the peptide that will displace 50% of a standard peptide from the HLA molecule. The lower the IC_50_ value, the stronger is the affinity of the peptide for the tested HLA molecule. According to the NetMHC parameters, peptides with IC_50_ < 50 nM were considered high-affinity binders. IC_50_ values of 5 nM and 500 nM were also tested, but a cutoff of 50 nM was chosen as the best indicator (at a 5 nM threshold not enough peptides were predicted to bind; at 500 nM all possible peptides within a given proteins were predicted to bind). Furthermore, all predicted epitopes were tested with NetChop 3.1 [89] to predict whether the epitopes could have been produced by the human proteasome using default parameters. All strong binding peptides with a high likelihood of being correctly cleaved (score prediction higher than the default threshold of 0.5) were included in further analyses.

To calculate the fraction of substituted amino acids within and outside of HLA epitopes, the substitutions detected in the specific viral populations of each patient were mapped onto viral reference proteins, and the number of substitutions that occurred within and outside of the predicted epitopes were calculated for each protein and HLA allele of each patient and donor respectively. The counts were normalized to the number of potentially mutable amino acids per category (i.e., within or outside of epitopes), to make them comparable across proteins of varying length.

Statistical comparison of the internal and external fractions was performed with a one-sided Wilcoxon signed test for each HLA allele to identify the direction of the difference. The *P*-values were Bonferroni corrected to account for multiple testing.

## Supporting information

S1 Fig BK polyomavirus proteins and location of predicted epitopes. (A) Agnoprotein, (B) VP1, (C) VP2, (D) VP3, (E) large T antigen “LTA” and (F) small t antigen “stA”. Variable amino acids are shown in yellow. Location of predicted epitopes for each protein presented by HLA-A, -B and -C are presented in grey.
(TIF)

S2 Fig Root-to-tip regression analysis for whole-genome BK polyomavirus sequences. **The** root-to-tip genetic distance against sampling time is shown for the BK polyomavirus phylogeny with a maximum sampling time of 568 days. The sampling time is given in days (R^2^ = 0.086, *P* < 0.05).
(TIF)

S1 Table Patients and samples enrolled in this study. The transplant organ, whether the patient developed the associated nephropathy BKVAN, source (urine/blood), viral load, total number of polymorphisms found in each sample and median coverage are represented.
(PDF)

S2 Table Single nucleotide polymorphisms in the coding regions found in the 225 samples (from 96 patients). The genomic position as well the reference and substitution nucleotides and amino acids are shown. The percentage of samples in which the position was found is indicated. The genomic position and the reference base and amino acid correspond to the BKV Dunlop reference strain.
(PDF)

S3 Table Insertions and deletions detected in the viral genomes of the 225 samples (from 96 patients). The positions, locus, reference and polymorphism, and percentage of samples with the polymorphism are shown. The genomic position and the reference base according to the BKV Dunlop reference strain.
(PDF)

S4 Table Inter-patient substitution rates. Summary of interpatient evolutionary rate estimates (substitutions/site/year, s/s/y) of BKV using different molecular clock (strict, relaxed log-normal uncorrelated and relaxed exponential uncorrelated) and demography (constant size and Bayesian skyline) models. Median and 95% high-density interval (HDI) intervals are shown. Estimates were obtained after two independent runs of 30 million generations each with a 10% burn-in. Convergence of the runs (ESS > 200) was checked with Tracer.
(PDF)

S5 Table Allele frequencies of MHC class I in our cohort. Alleles are shown for HLA-A, -B and -C at the 2nd field of resolution for donors and recipients.
(PDF)

S6 Table **BK polyomavirus predicted epitopes presented by HLA-A, -B and -C by protein**. Agnoprotein, VP1-3, large T antigen “LTA” and small t antigen “stA” predicted peptides presented by HLA-A, -B and -C from the BK polyomavirus Dunlop reference strain are listed. The starting and ending amino acid of the protein, length of the peptide, peptide sequence, and HLA allele that can present peptide are shown. The IC_50_ for each peptide and specific HLA allele are also included.
(PDF)
