# Supplementary figures and images for: An unusually high substitution rate in transplant-associated BK polyomavirus in vivo is further concentrated in HLA-C-bound viral peptides
Source: PLoS Pathog. 2018 Oct 18;14(10):e1007368. doi: 10.1371/journal.ppat.1007368 (PMC6207329; doi:10.1371/journal.ppat.1007368)

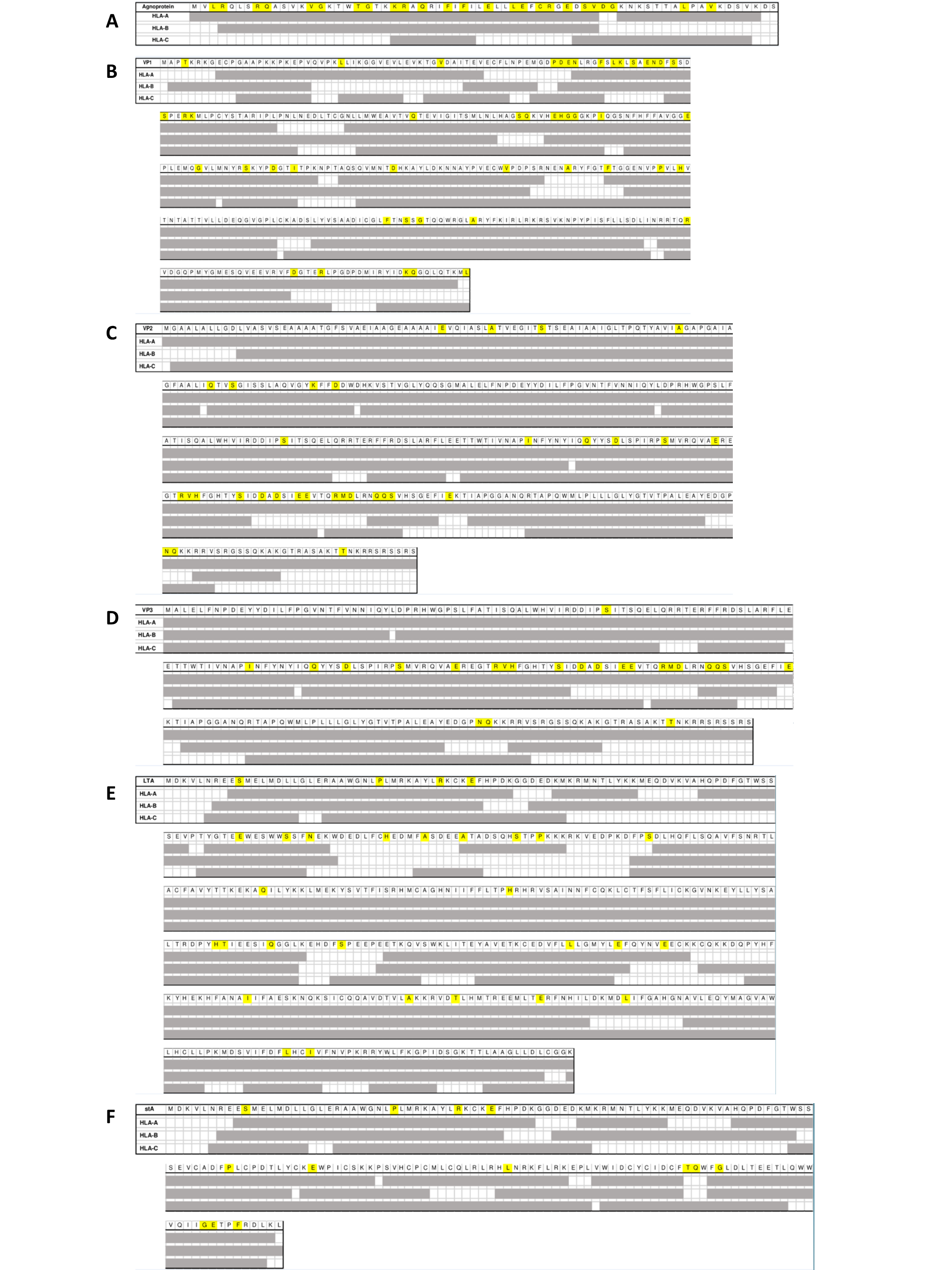

Supplement: S1 Fig — (A) Agnoprotein, (B) VP1, (C) VP2, (D) VP3, (E) large T antigen “LTA” and (F) small t antigen “stA”. Variable amino acids are shown in yellow. Location of predicted epitopes for each protein presented by HLA-A, -B and -C are presented in grey. (TIF) [file ppat.1007368.s001.tif]

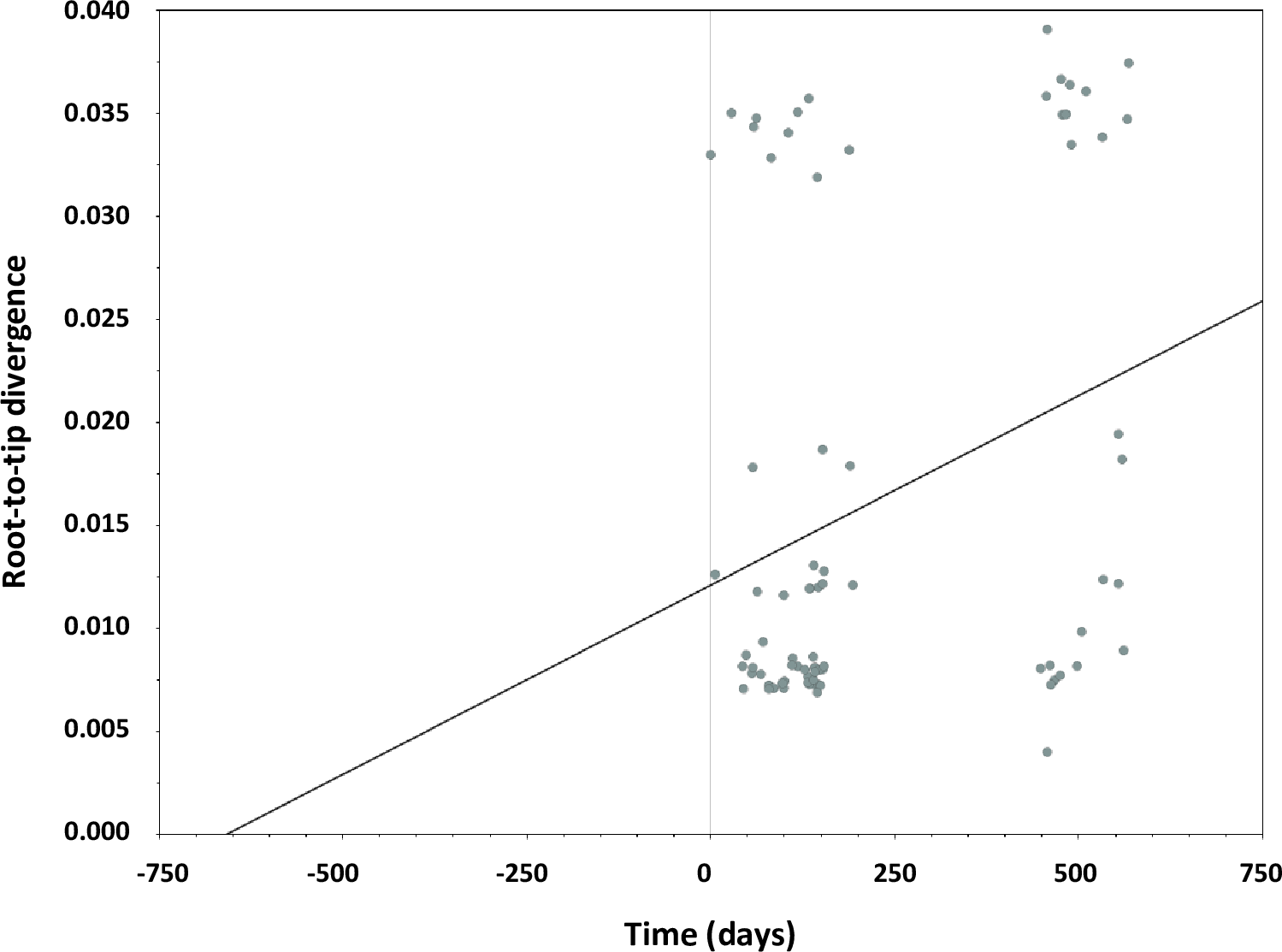

Supplement: S2 Fig — The root-to-tip genetic distance against sampling time is shown for the BK polyomavirus phylogeny with a maximum sampling time of 568 days. The sampling time is given in days (R2 = 0.086, P < 0.05). (TIF) [file ppat.1007368.s002.tif]
