# Supplementary material for: An unusually high substitution rate in transplant-associated BK polyomavirus in vivo is further concentrated in HLA-C-bound viral peptides
Source: PLoS Pathog. 2018 Oct 18;14(10):e1007368. doi: 10.1371/journal.ppat.1007368 (PMC6207329; doi:10.1371/journal.ppat.1007368)
Supplement: S1 Table — The transplant organ, whether the patient developed the associated nephropathy BKVAN, source (urine/blood), viral load, total number of polymorphisms found in each sample and median coverage are represented. (PDF) [file ppat.1007368.s003.pdf]

Patient	Transplant organ	Disease	Sample Code	Sampling day	Source	Viral load (copies/mL)	Number of polymorphisms	Median Coverage
1	Kidney	-	85	02/04/2012	Urine	1.28×10^5	206	3525.58
			110	28/02/2013	Urine	4.87×10^5	216	3373.36
			188	27/03/2013	Urine	3.14×10^5	210	4211.17
			401	26/06/2013	Urine	5.69×10^5	200	1471.41
2	Kidney	-	227	10/04/2013	Urine	1.60×10^8	227	2039.69
3	Kidney	-	250	18/04/2013	Urine	3.24×10^4	89	2996.34
			356	06/06/2013	Urine	4.47×10^4	94	2902.52
4	Kidney	BKVAN	106	25/02/2013	Urine	7.75×10^8	87	4307.11
			124	25/02/2013	Blood	9.66×10^4	199	1499.01
			136	06/03/2013	Urine	3.45×10^8	87	4294.85
			166	22/03/2013	Blood	1.55×10^5	91	900.84
			177	22/03/2013	Urine	3.54×10^9	94	78.89
			205	02/04/2013	Blood	7.31×10^5	86	2535.83
			206	02/04/2013	Urine	1.68×10^9	83	3280.95
			259	26/04/2013	Blood	7.84×10^4	87	2608.37
			260	26/04/2013	Urine	1.97×10^7	88	3308.75
			311	17/05/2013	Blood	5.50×10^4	84	1730.06
			312	17/05/2013	Urine	5.30×10^8	87	3481.97
			411	28/06/2013	Urine	2.58×10^5	87	3572.11
			455	19/07/2013	Urine	4.89×10^4	87	3652.80
5	Hematopoietic cells	-	145	11/03/2013	Urine	1.74×10^6	77	3322.81
6	Kidney	-	66	23/04/2012	Urine	4.20×10^9	237	2526.34
7	Kidney	-	112	28/02/2013	Urine	7.84×10^5	81	4114.20

			310	17/05/2013	Urine	1.01×10^{7}	80	4194.10
8	Kidney	-	217	03/04/2013	Urine	3.24×10^{6}	86	3056.76
			338	29/05/2013	Urine	7.51×10^{9}	88	3631.38
			475	26/07/2013	Urine	6.10×10^{6}	90	3988.68
9	Hematopoietic cells	-	326	23/05/2013	Urine	4.83×10^{4}	207	2621.78
10	Hematopoietic cells	-	358	06/06/2013	Urine	3.24×10^{4}	87	3168.83
11	Kidney	-	321	21/05/2013	Urine	8.29×10^{4}	88	3251.38
12	Kidney	-	74	13/04/2012	Urine	2.03×10^{7}	89	3111.96
13	Kidney	-	323	22/05/2013	Urine	1.55×10^{5}	78	3169.21
			460	22/07/2013	Urine	5.10×10^{6}	78	3667.22
14	Kidney	-	467	24/07/2013	Urine	2.19×10^{9}	223	2777.15
15	Kidney	-	109	26/02/2013	Urine	9.28×10^{6}	89	4390.23
			126	26/02/2013	Blood	4.93×10^{4}	92	1075.91
16	Kidney	-	101	22/02/2013	Urine	2.40×10^{8}	84	4959.78
			115	04/03/2013	Urine	6.74×10^{8}	86	4159.59
			214	02/04/2013	Urine	1.06×10^{8}	85	3660.63
			272	02/05/2013	Blood	3.99×10^{4}	79	2045.59
			273	02/05/2013	Urine	4.72×10^{8}	83	3473.22
			307	16/05/2013	Blood	6.82×10^{4}	86	913.72
			308	16/05/2013	Urine	1.56×10^{9}	85	3515.31
			389	20/06/2013	Urine	6.77×10^{8}	88	3789.52
			424	04/07/2013	Urine	7.48×10^{8}	88	4342.32
17	Kidney	-	103	22/02/2013	Urine	6.65×10^{5}	97	4206.71
18	Hematopoietic cells	-	97	16/01/2013	Urine	1.67×10^{10}	42	4093.16
			107	25/02/2013	Urine	4.86×10^{9}	40	3553.37

			144	11/03/2013	Urine	5.50×10^9	43	3278.05
19	Kidney	-	102	22/02/2013	Urine	1.88×10^9	78	3920.65
			189	27/03/2013	Urine	1.02×10^9	80	3118.98
			289	10/05/2013	Urine	1.25×10^9	80	4820.01
			361	07/06/2013	Urine	5.86×10^8	78	3868.72
			428	05/07/2013	Urine	1.61×10^8	84	4241.46
20	Lung	-	190	27/03/2013	Urine	1.24×10^8	94	3349.74
21	Kidney	BKVAN	45	10/01/2012	Urine	6.66×10^7	87	4697.85
			440	12/07/2013	Urine	1.40×10^6	95	4156.54
22	Kidney	-	104	22/02/2013	Urine	8.97×10^5	44	4619.11
			119	05/03/2013	Urine	4.31×10^6	45	4859.80
23	Kidney-Heart	-	69	05/04/2012	Urine	2.87×10^9	89	4195.00
			122	05/03/2013	Urine	1.57×10^8	94	2902.52
			147	12/03/2013	Urine	1.57×10^8	100	3320.05
			196	05/04/2012	Urine	3.13×10^8	92	3165.89
			264	29/04/2013	Urine	7.45×10^7	96	3473.79
			290	10/05/2013	Urine	2.39×10^7	96	4097.09
			414	28/06/2013	Urine	2.46×10^6	94	3062.33
			443	12/07/2013	Urine	1.07×10^7	94	3004.62
24	Kidney	-	96	23/03/2012	Urine	6.42×10^7	231	2324.96
			224	05/04/2013	Urine	6.90×10^7	219	2564.30
			345	31/05/2013	Urine	7.97×10^6	217	2820.29
			429	05/07/2013	Urine	2.01×10^7	213	2523.24
25	Hematopoietic cell	-	336	28/05/2013	Urine	1.31×10^9	88	2644.57
			384	19/06/2013	Urine	1.03×10^7	86	3189.81

26	Kidney	BKVAN	89	13/03/2012	Urine	1.30×10^5	91	3946.81
			146	12/03/2013	Urine	2.18×10^7	95	3361.77
27	Kidney	-	59	19/04/2012	Urine	1.70×10^5	89	1781.02
28	Lung	-	148	12/03/2013	Urine	2.33×10^7	88	2718.09
29	Kidney	-	26	07/02/2012	Urine	5.71×10^9	79	4129.27
30	Kidney	-	140	07/03/2013	Urine	5.15×10^6	88	3023.00
			221	04/04/2013	Urine	4.16×10^6	88	3445.18
			275	02/05/2013	Urine	4.87×10^6	87	4204.63
			469	25/07/2013	Urine	5.35×10^4	87	3729.29
31	Kidney	BKVAN	41	03/01/2012	Urine	8.11×10^7	226	2657.17
			244	17/04/2013	Urine	5.38×10^6	200	2231.70
			470	25/07/2013	Urine	5.38×10^6	220	2396.03
32	Lung	-	99	20/02/2013	Urine	9.29×10^6	75	5163.43
33	Kidney	BKVAN	417	01/07/2013	Urine	2.48×10^4	82	2344.34
34	Lung	-	120	05/03/2013	Urine	1.37×10^8	90	4049.26
35	Kidney	-	3	01/03/2012	Urine	6.96×10^6	219	2711.70
			375	12/06/2013	Urine	4.65×10^7	232	2348.83
36	Kidney	-	42	05/01/2012	Urine	3.22×10^5	205	3914.14
37	Kidney	BKVAN	117	04/03/2013	Urine	2.17×10^5	90	4296.58
38	Kidney	BKVAN	116	04/03/2013	Urine	1.35×10^9	86	3567.42
			125	25/02/2013	Blood	1.72×10^4	85	2148.74
			134	14/03/2013	Blood	5.40×10^4	88	1421.38
			154	14/03/2013	Urine	1.33×10^9	85	3119.26
			231	17/04/2013	Blood	8.13×10^4	87	29.47
			232	17/04/2013	Urine	1.63×10^8	83	3336.37

			261	29/04/2013	Urine	6.38×10^{9}	82	5276.67
			294	13/05/2013	Blood	1.25×10^{4}	83	43.05
			295	13/05/2013	Urine	4.26×10^{9}	86	3409.84
			366	10/06/2013	Urine	2.21×10^{9}	85	4168.20
			419	02/07/2013	Urine	4.72×10^{8}	86	3033.91
			446	15/07/2013	Urine	3.41×10^{9}	83	3242.63
			473	26/07/2013	Urine	2.85×10^{9}	86	4412.25
39	Kidney	-	6	05/03/2012	Urine	2.30×10^{8}	88	4383.84
40	Kidney	BKVAN	77	18/04/2012	Urine	1.59×10^{7}	84	3232.55
41	Kidney	-	93	21/03/2012	Urine	1.52×10^{7}	210	1097.98
42	Kidney	-	34	17/01/2012	Urine	4.27×10^{4}	80	2209.43
43	Kidney	-	32	12/01/2012	Urine	8.57×10^{9}	39	4060.45
44	Hematopoietic Cells	-	265	29/04/2013	Urine	1.19×10^{10}	87	3427.05
45	Kidney	-	223	05/04/2013	Urine	2.92×10^{9}	85	3126.17
			288	10/05/2013	Urine	1.22×10^{9}	89	4081.04
			377	14/06/2013	Urine	3.69×10^{8}	89	3633.49
			472	26/07/2013	Urine	5.31×10^{7}	91	4653.67
46	Kidney	-	30	08/02/2012	Urine	1.63×10^{8}	220	1952.51
47	Kidney	-	172	18/03/2013	Urine	2.17×10^{10}	91	3117.66
			219	04/04/2013	Urine	4.81×10^{9}	88	3044.41
			262	29/04/2013	Urine	1.15×10^{10}	90	3773.04
			318	21/05/2013	Urine	2.74×10^{10}	90	3769.08
			365	10/06/2013	Blood	8.09×10^{4}	91	111.67
			367	10/06/2013	Urine	3.33×10^{10}	91	4605.01
			399	25/06/2013	Urine	5.41×10^{9}	91	2718.62

			432	08/07/2013	Urine	8.14×10^9	92	3704.41
48	Hematopoietic cell	-	283	06/05/2013	Urine	7.76×10^7	87	894.30
49	Kidney	-	243	16/04/2013	Urine	1.04×10^7	79	3142.13
50	Lung	-	303	14/05/2013	Urine	2.55×10^9	97	2713.93
51	Hematopoietic cell	-	153	14/03/2013	Urine	1.53×10^{10}	85	2908.82
			242	16/04/2013	Urine	3.69×10^9	85	4065.94
52	Kidney	BKVAN	20	27/02/2012	Urine	2.82×10^7	88	4188.16
53	Kidney	-	139	07/03/2013	Urine	1.16×10^7	83	3039.86
54	Kidney	-	277	03/05/2013	Urine	6.43×10^6	202	1980.24
55	Lung	-	339	29/05/2013	Urine	2.22×10^6	87	3317.25
56	Kidney	-	82	28/03/2012	Urine	1.67×10^8	218	2357.51
57	Kidney	-	113	01/03/2013	Urine	8.63×10^7	220	3035.92
			121	05/03/2013	Urine	5.64×10^7	223	2487.83
			127	01/03/2013	Blood	3.20×10^4	180	427.55
			128	05/03/2013	Blood	5.69×10^4	172	422.88
			208	02/04/2013	Urine	7.49×10^6	217	2235.23
			271	02/05/2013	Urine	9.08×10^6	216	2931.36
58	Kidney	-	213	28/03/2013	Urine	4.79×10^6	85	3166.76
59	Lung	-	403	26/06/2013	Urine	2.73×10^8	221	1949.47
60	Kidney	-	370	11/06/2013	Urine	1.16×10^4	90	3494.72
61	Hematopoietic cell	-	335	28/05/2013	Urine	5.78×10^4	40	3318.41
			369	10/06/2013	Urine	9.95×10^5	80	3963.16
			422	02/07/2013	Urine	2.98×10^4	40	3884.24
62	Kidney	-	111	28/02/2013	Urine	1.12×10^6	88	3899.97
			439	11/07/2013	Urine	4.70×10^6	86	4042.02

63	Kidney	-	16	17/02/2012	Urine	1.08×10^{5}	180	645.16
64	Lung-Heart	-	35	17/01/2012	Urine	2.20×10^{9}	48	4281.55
65	Kidney	BKVAN	105	25/02/2013	Urine	9.55×10^{7}	88	4165.68
			160	15/03/2013	Urine	4.78×10^{9}	85	4583.10
			215	02/04/2013	Urine	5.35×10^{9}	92	3355.17
			266	30/04/2013	Urine	4.07×10^{10}	89	5072.37
			287	10/05/2013	Urine	7.55×10^{8}	88	4572.30
			349	03/06/2013	Urine	4.50×10^{9}	90	3722.81
			393	21/06/2013	Urine	5.56×10^{8}	91	3223.45
			462	22/07/2013	Urine	1.39×10^{9}	90	4292.28
66	Hematopoietic cell	-	98	17/01/2013	Urine	4.23×10^{9}	205	3481.96
67	Lung	-	118	04/03/2013	Urine	1.48×10^{8}	86	4209.22
68	Hematopoietic cell	-	108	25/02/2013	Urine	9.72×10^{5}	63	1795.48
69	Kidney	-	53	02/05/2012	Urine	1.68×10^{5}	89	922.64
			182	25/03/2013	Urine	2.90×10^{5}	86	3020.13
			247	18/04/2013	Urine	3.12×10^{8}	92	3335.01
			382	18/06/2013	Urine	3.93×10^{7}	92	3963.22
			430	05/07/2013	Urine	1.64×10^{8}	92	3477.82
			454	18/07/2013	Urine	2.10×10^{6}	92	4010.13
70	Kidney	BKVAN	31	12/01/2013	Urine	7.14×10^{9}	80	3746.17
71	Kidney	-	137	06/03/2013	Urine	1.16×10^{8}	115	3254.26
			376	12/06/2013	Urine	5.00×10^{7}	158	3534.12
72	Lung	-	181	25/03/2013	Urine	2.81×10^{10}	96	3070.61
73	Kidney	-	453	18/07/2013	Urine	1.11×10^{6}	79	3710.45
74	Kidney	-	220	04/04/2013	Urine	1.98×10^{6}	93	3782.87

75	Kidney	-	67	24/04/2012	Urine	2.72×10^{7}	219	2739.43
76	Kidney-Pancreas	-	65	23/04/2012	Urine	1.72×10^{5}	136	874.04
77	Kidney	BKVAN	157	14/03/2013	Urine	1.28×10^{7}	87	3091.79
			284	06/05/2013	Urine	6.20×10^{9}	88	4402.07
			449	15/07/2013	Urine	2.76×10^{9}	87	3629.62
78	Kidney	-	280	06/05/2013	Blood	6.45×10^{4}	90	120.57
			281	06/05/2013	Urine	4.09×10^{8}	87	3302.82
			387	20/06/2013	Urine	2.71×10^{9}	88	3640.69
			459	22/07/2013	Urine	7.27×10^{8}	87	4788.12
79	Kidney	BKVAN	314	17/05/2013	Urine	3.79×10^{9}	86	3813.18
			342	31/05/2013	Urine	2.03×10^{8}	85	1226.93
			413	28/06/2013	Blood	7.38×10^{4}	59	2009.53
			442	12/07/2013	Urine	1.60×10^{9}	87	4615.58
			456	19/07/2013	Blood	1.11×10^{5}	85	1683.20
			457	19/07/2013	Urine	9.88×10^{8}	88	4099.06
			463	23/07/2013	Blood	1.40×10^{5}	86	1522.66
			464	23/07/2013	Urine	4.82×10^{9}	87	4475.89
80	Kidney	-	237	15/04/2013	Urine	1.70×10^{5}	85	1455.13
81	Kidney	-	364	10/06/2013	Urine	2.97×10^{6}	88	2361.61
82	Kidney	BKVAN	151	13/03/2013	Urine	4.49×10^{8}	219	3050.83
			226	10/04/2013	Urine	6.64×10^{8}	210	1894.78
			305	15/05/2013	Urine	9.43×10^{8}	226	2536.32
			434	10/07/2013	Urine	2.93×10^{8}	206	1804.97
83	Lung	-	257	18/04/2013	Blood	4.06×10^{4}	138	1553.08
84	Kidney	-	25	06/02/2012	Urine	2.08×10^{5}	100	1350.93

85	Kidney	BKVAN	193	03/04/2012	Blood	3.80×10^{3}	168	101.25
86	Kidney	-	149	12/03/2013	Urine	4.71×10^{5}	78	1911.90
			229	16/04/2013	Blood	1.46×10^{4}	94	813.86
			230	16/04/2013	Urine	3.51×10^{7}	78	3940.28
			268	30/04/2013	Blood	2.04×10^{4}	80	1812.80
			269	30/04/2013	Urine	3.11×10^{7}	76	3382.60
			301	14/05/2013	Blood	2.23×10^{4}	103	175.20
			302	14/05/2013	Urine	4.35×10^{7}	78	3343.33
			333	28/05/2013	Blood	2.17×10^{4}	79	1953.84
			334	28/05/2013	Urine	2.01×10^{7}	78	4242.63
			420	02/07/2013	Urine	6.82×10^{5}	78	3392.39
87	Kidney	-	355	06/06/2013	Urine	1.07×10^{5}	95	1375.44
88	Kidney	-	88	04/04/2012	Urine	2.39×10^{9}	223	2581.30
89	Kidney	-	423	03/07/2013	Urine	7.11×10^{5}	122	2897.71
90	Kidney	BKVAN	191	28/03/2013	Urine	1.49×10^{9}	210	2583.98
91	Hematopoietic cells	-	331	27/05/2013	Urine	1.05×10^{10}	203	3610.15
			471	25/07/2013	Urine	4.12×10^{4}	199	1937.03
92	Kidney	-	7	07/03/2012	Urine	1.29×10^{3}	73	53.89
93	Lung	-	100	20/02/2013	Urine	1.84×10^{10}	88	3623.62
			123	20/02/2013	Blood	1.52×10^{5}	88	1826.52
			165	22/03/2013	Blood	2.17×10^{5}	70	1182.31
94	Lung	-	407	27/06/2013	Blood	2.20×10^{4}	83	1454.54
95	Kidney	-	240	16/04/2013	Urine	4.44×10^{5}	87	3047.68
			397	24/06/2013	Urine	2.37×10^{6}	86	3730.36
96	Hematopoietic cells	-	114	01/03/2013	Urine	3.08×10^{9}	87	3898.58

239	15/04/2013	Urine	1.13×10^{10}	87	4043.74
291	10/05/2013	Urine	4.08×10^{7}	86	4449.19
