# Supplementary material for: An unusually high substitution rate in transplant-associated BK polyomavirus in vivo is further concentrated in HLA-C-bound viral peptides
Source: PLoS Pathog. 2018 Oct 18;14(10):e1007368. doi: 10.1371/journal.ppat.1007368 (PMC6207329; doi:10.1371/journal.ppat.1007368)
Supplement: S2 Table — The genomic position as well the reference and substitution nucleotides and amino acids are shown. The percentage of samples in which the position was found is indicated. The genomic position and the reference base and amino acid correspond to the BKV Dunlop reference strain. (PDF) [file ppat.1007368.s004.pdf]

Locus	Genomic position	Gene position	Reference base	Sample base	Reference amino acid	Sample amino acid	Percentage of samples
**NCCR**	20		G	T			0.44
	20		G	A			2.67
	22		A	G			0.88
	41		G	T			13.78
	57		G	T			0.44
	84		G	C			12.44
	84		G	T			0.44
	85		A	T			3.11
	86		G	A			3.55
	102		A	C			15.11
	102		A	G			0.44
	160		T	A			13.78
	161		A	T			11.56
	165		A	T			1.33
	173		C	T			13.78
	174		A	G			0.44
	184		T	G			0.88
	193		G	A			17.78
	197		G	C			4.44
	197		G	T			0.88
	198		A	C			4.88
	208		A	G			0.44
	211		G	T			1.33
	217		C	G			6.22
	236		A	G			8.89
	239		G	A			0.44
	253		A	G			81.33
	255		A	C			4.89
	261		C	T			1.78
	261		C	G			0.44
	274		G	A			0.44
	283		G	C			0.44
	287		C	G			10.67
	289		G	A			12.00
	290		G	T			4.89
	297		G	T			0.44
	310		C	A			0.44
	317		G	A			1.78
	342		A	G			63.56
	346		T	G			11.56

	348		G	A			4.89
	353		C	T			1.33
	353		C	G			0.88
	362		G	T			0.44
	364		C	G			1.33
	377		G	T			0.44
	386		C	G			1.33
**Agnoprotein**	394	7	C	G	L	V	3.11
	396	9	G	A	L	L	12.44
	398	11	G	T	R	L	0.89
	410	23	G	A	R	Q	2.22
	412	25	C	G	Q	E	0.44
	420	33	A	T	S	S	92.44
	427	40	G	A	V	I	4.89
	427	40	G	C	V	L	64.0
	429	42	T	A	V	V	0.89
	430	43	G	A	G	S	25.33
	443	56	C	G	T	S	0.44
	446	59	G	A	G	E	0.44
	450	63	A	T	T	T	16.0
	455	68	A	G	K	R	0.89
	458	71	G	C	R	T	2.67
	459	72	A	G	R	R	0.44
	463	76	C	G	Q	E	0.44
	465	78	G	C	Q	H	3.11
	472	85	T	C	F	L	11.11
	479	92	T	A	F	Y	0.44
	487	100	G	C	E	Q	0.44
	487	100	G	T	E	*	0.44
	496	109	C	G	L	V	0.44
	499	112	G	A	E	K	0.44
	499	112	G	C	E	Q	0.44
	506	119	G	T	C	F	1.78
	507	120	T	G	C	W	0.89
	509	122	G	A	R	K	0.44
	514	127	G	A	E	K	11.11
	515	128	A	T	E	V	0.44
	520	133	A	G	S	G	0.44
	522	135	T	G	S	R	0.44
	523	136	G	A	V	I	4.89
	526	139	G	A	D	N	0.44

	529	142	G	T	G	W	0.44
	553	166	T	G	L	V	10.22
	566	179	A	G	K	R	0.44
	585	198	C	A	S	S	0.44
**Intergenic**	598		C	G			5.33
	604		T	G			0.44
	623		C	G			87.11
**VP2**	734	111	T	C	A	A	4.89
	738	115	G	C	E	Q	0.44
	759	136	G	T	A	S	0.89
	781	158	G	C	S	T	4.89
	815	192	T	A	T	T	4.89
	837	214	G	A	A	T	0.44
	848	225	T	A	P	P	19.11
	875	252	A	G	L	L	4.0
	878	255	T	C	I	I	0.44
	879	256	C	G	Q	E	0.44
	889	266	G	C	S	T	94.67
	902	279	C	T	S	S	18.22
	905	282	A	G	L	L	99.11
	922	299	A	G	K	R	92.89
	926	303	C	T	F	F	79.11
	930	307	G	A	D	N	73.78
	930	307	G	T	D	Y	11.56
	931	308	A	G	D	G	74.22
	965	342	C	T	L	L	19.11
**VP2 / VP3**	986	363 / 6	T	A	A / A	A / A	10.22
	990	367 / 10	G	C	E / E	Q / Q	0.44
	1008	385 / 28	G	C	E / E	Q / Q	0.44
	1022	399 / 42	T	A	I / I	I / I	66.67
	1023	400 / 43	C	T	L / L	L / L	94.67
	1049	426 / 69	T	A	V / V	V / V	94.67
	1064	441 / 84	C	T	Y / Y	Y / Y	4.89
	1067	444 / 87	T	A	L / L	L / L	18.67
	1091	468 / 111	T	C	S / S	S / S	11.56
	1109	486 / 129	C	T	S / S	S / S	72.44
	1133	510 / 153	G	A	R / R	R / R	4.89
	1146	523 / 166	T	G	S / S	A / A	94.67
	1154	531 / 174	C	T	T / T	T / T	17.33
	1160	537 / 180	G	A	Q / Q	Q / Q	93.78
	1169	546 / 189	G	A	Q / Q	Q / Q	93.78

1172	549 / 192	A	G	R / R	R / R	4.0
1175	552 / 195	A	G	R / R	R / R	1.78
1181	558 / 201	A	G	E / E	E / E	13.33
1187	564 / 207	T	C	F / F	F / F	13.33
1193	570 / 213	A	G	R / R	R / R	4.44
1199	576 / 219	C	T	S / S	S / S	4.44
1217	594 / 237	G	A	E / E	E / E	19.56
1223	600 / 243	T	A	T / T	T / T	1.33
1223	600 / 243	T	C	T / T	T / T	4.89
1232	609 /252	A	C	T / T	T / T	5.78
1248	625 / 268	A	G	I / I	V / V	13.78
1250	627 / 270	A	G	I / I	M / M	2.22
1271	648 / 291	A	G	Q / Q	Q / Q	11.56
1272	649 / 292	C	G	Q / Q	E / E	95.11
1274	651 / 294	A	T	Q / Q	H / H	12.0
1284	661 / 304	G	A	D /D	N / N	0.44
1287	664 / 307	C	T	L / L	L / L	6.22
1289	666 / 309	T	G	L / L	L / L	8.89
1304	681 / 324	C	T	P / P	P / P	17.33
1306	683 / 326	C	T	S / S	L / L	0.44
1313	690 / 333	T	C	V / V	V / V	0.44
1316	693 / 336	A	G	R / R	R / R	17.33
1322	699 / 342	A	T	V / V	V / V	12.89
1322	699 / 342	A	G	V / V	V / V	12.0
1326	703 / 346	G	A	E / E	K / K	1.33
1337	714 / 357	T	A	G / G	G / G	11.11
1342	719 / 362	G	A	R / R	H / H	5.33
1344	721 / 364	G	T	V / V	L / L	0.44
1347	724 / 367	C	A	H / H	N / N	5.33
1361	738 / 381	T	C	T / T	T / T	14.67
1364	741 / 384	T	C	Y / Y	Y / Y	14.67
1367	744 / 387	T	A	S / S	R / R	12.0
1367	744 / 387	T	C	S / S	S / S	4.44
1374	751/ 394	G	A	D /D	N / N	0.44
1374	751 / 394	G	C	D /D	H / H	1.78
1380	757 / 400	G	A	D /D	N / N	0.89
1389	766 / 409	G	A	E / E	K / K	0.89
1389	766 / 409	G	C	E / E	Q / Q	13.33
1392	769/ 412	G	A	E / E	K / K	0.44
1400	777 / 420	A	C	T / T	T / T	17.33
1405	782 / 425	G	A	R / R	K / K	0.44

	1409	786 / 429	G	A	M / M	I / I	4.44
	1412	789 / 432	C	T	D /D	D /D	5.78
	1412	789 / 432	C	G	D /D	E / E	17.33
	1422	799 / 442	C	A	Q / Q	K / K	7.11
	1424	801 / 444	A	G	Q / Q	Q / Q	3.56
	1425	802 / 445	C	G	Q / Q	E / E	5.78
	1427	804 / 447	A	G	Q / Q	Q / Q	2.67
	1429	806 / 449	G	C	S / S	T / T	91.11
	1452	829 / 472	G	A	E / E	K / K	0.44
	1514	891 / 534	A	G	L / L	L / L	19.56
**VP2 / VP3 / VP1**	1574	951 / 594 / 11	C	A	N / N / T	K / K / N	0.89
	1575	952 / 595 / 12	C	A	Q / Q / T	K / K / T	64.89
	1644	1021 / 664 / 81	A	G	T / T / L	A / A / L	32.0
**VP1**	1687	124	G	C	V	L	66.22
	1698	135	T	A	I	I	93.33
	1704	141	G	A	E	E	14.22
	1716	153	C	T	F	F	17.78
	1722	159	C	T	N	N	13.33
	1737	174	T	C	D	D	0.89
	1738	175	C	G	P	A	0.44
	1741	178	G	A	D	N	1.33
	1741	178	G	C	D	H	1.78
	1744	181	G	A	E	K	11.11
	1746	183	A	T	E	D	15.11
	1747	184	A	C	N	H	2.22
	1747	184	A	T	N	Y	0.44
	1747	184	A	G	N	D	8.44
	1760	197	T	A	F	Y	15.56
	1765	202	C	G	L	V	1.78
	1766	203	T	A	L	Q	0.89
	1769	206	A	G	K	R	0.89
	1770	207	G	A	K	K	2.22
	1770	207	G	C	K	N	0.89
	1775	212	G	C	S	T	5.78
	1780	217	G	A	E	K	3.11
	1780	217	G	C	E	Q	5.33
	1781	218	A	C	E	A	0.44
	1784	221	A	C	N	T	7.56
	1786	223	G	A	D	N	5.33
	1787	224	A	C	D	A	12.0
	1788	225	C	T	D	D	0.44

1792	229	A	C	S	R	0.89
1792	229	A	G	S	G	11.11
1793	230	G	A	S	N	12.0
1794	231	C	A	S	R	0.44
1803	240	C	G	S	R	0.44
1807	244	G	A	E	K	0.44
1807	244	G	C	E	Q	4.0
1809	246	G	A	E	E	67.56
1809	246	G	C	E	D	27.11
1811	248	G	A	R	K	5.33
1815	252	A	C	K	N	0.44
1824	261	C	T	P	P	4.44
1848	285	C	A	P	P	17.33
1851	288	C	A	L	L	8.89
1851	288	C	G	L	L	11.11
1854	291	C	T	P	P	8.0
1858	295	T	C	L	L	4.89
1860	297	A	G	L	L	8.0
1869	306	C	T	D	D	12.89
1890	327	G	A	L	L	19.56
1905	342	A	G	V	V	13.78
1908	345	T	A	T	T	67.11
1912	349	C	A	Q	K	19.11
1923	360	T	C	V	V	75.11
1938	375	C	T	S	S	14.22
1960	397	T	G	S	A	0.89
1962	399	A	C	S	S	4.0
1963	400	C	A	Q	K	1.33
1965	402	A	G	Q	Q	13.33
1971	408	G	A	V	V	13.33
1971	408	G	T	V	V	4.0
1977	414	G	A	E	E	10.67
1977	414	G	C	E	D	0.89
1978	415	C	A	H	N	19.11
1980	417	T	A	H	Q	0.44
1985	422	G	T	G	V	1.33
1986	423	A	T	G	G	0.44
1988	425	G	A	G	E	0.44
1989	426	A	C	G	G	16.89
1989	426	A	T	G	G	77.33
1996	433	A	G	I	V	4.44

1998	435	T	C	I	I	3.56
1998	435	T	G	I	M	0.89
2007	444	T	C	S	S	12.0
2013	450	C	T	F	F	12.0
2019	456	C	T	F	F	76.0
2028	465	A	T	V	V	88.0
2028	465	A	G	V	V	8.44
2034	471	A	G	G	G	6.22
2037	474	A	C	E	D	97.33
2054	491	G	C	G	A	1.33
2058	495	G	A	V	V	11.11
2061	498	A	T	L	L	12.44
2067	504	T	C	N	N	0.89
2073	510	G	A	R	R	7.11
2074	511	T	A	S	T	93.78
2076	513	A	C	S	S	12.44
2085	522	T	A	P	P	91.56
2086	523	G	C	D	H	5.78
2088	525	T	A	D	E	75.56
2095	532	A	G	I	V	2.67
2103	540	T	A	P	P	86.22
2106	543	A	G	K	K	0.44
2109	546	C	T	N	N	2.67
2112	549	A	C	P	P	0.44
2112	549	A	T	P	P	6.22
2118	555	C	T	A	A	18.67
2127	564	G	A	Q	Q	67.11
2139	576	T	A	T	T	0.44
2140	577	G	A	D	N	0.89
2142	579	C	T	D	D	4.89
2142	579	C	G	D	E	0.44
2154	591	T	C	Y	Y	13.78
2172	609	T	C	A	A	0.44
2184	621	G	A	E	E	13.78
2191	628	G	A	V	I	75.56
2193	630	A	T	V	V	91.56
2199	636	T	C	D	D	1.33
2202	639	C	T	P	P	12.44
2218	655	G	A	A	T	98.22
2223	660	G	A	R	R	0.89
2232	669	G	A	G	G	18.67

2235	672	T	A	T	T	13.33
2237	674	T	A	F	Y	18.22
2238	675	C	T	F	F	0.89
2244	681	A	G	G	G	2.22
2261	698	C	A	P	Q	0.44
2269	706	C	T	H	Y	0.44
2274	711	G	A	V	V	14.22
2274	711	G	T	V	V	4.89
2280	717	C	T	N	N	1.33
2301	738	A	G	L	L	18.67
2313	750	T	A	G	G	0.44
2322	759	T	C	P	P	4.89
2325	762	T	A	L	L	2.67
2325	762	T	G	L	L	16.89
2337	774	T	C	D	D	2.67
2370	807	C	A	G	G	12.44
2370	807	C	G	G	G	4.0
2375	812	T	C	F	S	0.44
2385	822	C	A	S	R	0.44
2391	828	A	G	G	G	5.33
2406	843	A	G	R	R	15.11
2413	850	G	C	A	P	15.11
2430	867	C	T	I	I	80.0
2451	888	A	G	V	V	5.33
2454	891	G	A	K	K	76.89
2457	894	T	C	N	N	15.56
2481	918	A	T	L	L	15.11
2481	918	A	G	L	L	8.44
2508	945	G	A	Q	Q	4.89
2510	947	G	A	R	K	4.89
2541	978	A	G	E	E	14.22
2544	981	C	T	S	S	17.33
2550	987	A	T	V	V	4.44
2550	987	A	G	V	V	10.22
2553	990	A	G	E	E	17.78
2559	996	T	C	V	V	13.78
2559	996	T	G	V	V	5.33
2562	999	G	A	R	R	1.33
2569	1006	G	C	D	H	0.44
2581	1018	A	C	R	R	19.11
2582	1019	G	A	R	K	43.56

	2583	1020	A	G	R	R	19.11
	2613	1050	T	C	Y	Y	0.44
	2619	1056	C	T	D	D	12.0
	2621	1058	A	G	K	R	20.44
	2623	1060	C	G	Q	E	0.44
	2625	1062	G	A	Q	Q	81.78
	2640	1077	C	A	T	T	20.0
	2640	1077	C	T	T	T	11.56
	2647	1084	C	G	L	V	32.44
**Intergenic**	2656		G	A			0.44
	2670		A	T			64.89
	2671		C	G			64.44
	2677		C	T			5.33
	2681		T	C			4.00
	2684		T	A			0.44
	2698		G	A			7.55
	2702		A	T			0.88
	2704		G	C			2.22
	2705		C	A			2.22
	2708		C	G			81.78
	2709		T	G			1.78
	2715		G	T			10.22
	2719		G	C			3.11
**LTA**	2746	2064	G	A	R	R	5.33
	2752	2058	T	C	F	F	0.44
	2758	2052	G	A	Q	Q	12.89
	2759	2051	A	T	Q	L	0.44
	2779	2031	G	A	L	L	20.0
	2782	2028	G	C	E	D	0.44
	2784	2026	G	C	E	Q	0.44
	2787	2023	C	G	Q	E	19.56
	2788	2022	T	C	S	S	5.33
	2791	2019	T	C	H	H	4.44
	2794	2016	C	T	P	P	4.44
	2801	2009	C	T	S	L	15.11
	2802	2008	T	C	S	P	14.22
	2802	2008	T	G	S	A	0.89
	2808	2002	C	A	Q	K	0.44
	2809	2001	C	A	S	S	4.0
	2814	1996	G	C	D	H	0.89
	2817	1993	G	A	E	K	2.22

2818	1992	T	A	A	A	0.89
2818	1992	T	C	A	A	1.78
2830	1980	T	C	T	T	0.44
2831	1979	C	A	T	N	1.78
2831	1979	C	G	T	S	0.44
2842	1968	A	G	Q	Q	0.44
2845	1965	C	A	S	S	3.56
2849	1961	C	T	S	F	0.44
2863	1947	C	A	S	S	0.89
2863	1947	C	T	S	S	13.78
2868	1942	G	A	E	K	0.44
2868	1942	G	C	E	Q	0.89
2906	1904	A	G	D	G	0.44
2907	1903	G	C	D	H	0.89
2908	1902	G	A	E	E	95.11
2920	1890	T	A	I	I	13.78
2920	1890	T	C	I	I	71.56
2938	1872	G	A	G	G	71.11
2959	1851	G	A	M	I	0.44
2966	1844	C	T	S	L	0.89
3012	1798	G	C	E	Q	0.44
3025	1785	A	G	Q	Q	20.44
3034	1776	T	A	T	T	99.56
3035	1775	C	A	T	N	18.22
3036	1774	A	C	T	P	14.22
3038	1772	C	T	A	V	2.22
3039	1771	G	T	A	S	4.44
3040	1770	T	C	F	F	1.33
3045	1765	G	A	D	N	0.44
3058	1752	T	C	F	F	14.67
3070	1740	G	T	L	L	0.44
3079	1731	G	T	L	F	1.33
3081	1729	T	C	L	L	1.78
3082	1728	C	T	T	T	1.33
3099	1711	T	C	L	L	44.0
3100	1710	T	A	I	I	20.44
3117	1693	T	C	L	L	0.89
3118	1692	C	T	F	F	25.78
3121	1689	G	A	E	E	14.22
3123	1687	G	A	E	K	0.89
3133	1677	A	G	L	L	19.11

3135	1675	T	C	L	L	15.11
3139	1671	A	G	K	K	5.78
3151	1659	A	T	I	I	10.22
3157	1653	C	T	P	P	11.11
3160	1650	G	A	R	R	30.22
3172	1638	A	G	Q	Q	93.33
3190	1620	A	G	Q	Q	95.11
3195	1615	C	T	L	L	14.22
3196	1614	C	T	T	T	5.33
3202	1608	T	C	P	P	13.78
3205	1605	C	T	V	V	13.33
3207	1603	G	T	V	F	1.33
3208	1602	T	A	P	P	3.56
3210	1600	C	T	P	S	0.89
3214	1596	G	A	E	E	1.78
3216	1594	G	A	E	K	0.89
3216	1594	G	C	E	Q	0.89
3229	1581	G	A	L	L	20.89
3231	1579	T	C	L	L	76.0
3232	1578	C	T	G	G	14.22
3232	1578	C	G	G	G	5.33
3238	1572	A	T	P	P	2.67
3250	1560	C	A	T	T	5.78
3265	1545	T	C	H	H	15.11
3271	1539	G	A	K	K	0.44
3283	1527	A	T	V	V	95.11
3286	1524	G	A	K	K	17.78
3287	1523	A	G	K	R	0.44
3288	1522	A	G	K	E	0.44
3289	1521	T	C	V	V	11.56
3298	1512	T	C	D	D	0.44
3303	1507	T	C	L	L	5.33
3310	1500	A	G	R	R	19.56
3313	1497	G	A	L	L	15.56
3351	1459	T	C	L	L	2.67
3367	1443	A	G	G	G	18.22
3370	1440	A	T	T	T	8.89
3372	1438	G	A	T	A	0.89
3373	1437	G	A	G	G	91.11
3376	1434	A	G	K	K	10.67
3379	1431	A	G	V	V	11.56

3400	1410	C	T	Y	Y	14.67
3415	1395	A	G	V	V	17.78
3421	1389	A	G	L	L	12.44
3424	1386	G	A	E	E	93.78
3430	1380	C	T	T	T	6.67
3433	1377	A	T	L	L	2.22
3433	1377	A	G	L	L	14.22
3450	1360	C	T	L	L	2.67
3451	1359	C	T	N	N	6.67
3454	1356	A	T	V	V	0.89
3454	1356	A	G	V	V	3.11
3463	1347	C	T	A	A	80.89
3469	1341	T	C	G	G	4.44
3472	1338	T	A	G	G	99.56
3478	1332	G	A	L	L	19.56
3481	1329	T	C	D	D	5.33
3490	1320	G	A	G	G	0.44
3501	1309	C	T	L	L	5.33
3502	1308	A	C	T	T	5.33
3511	1299	A	C	G	G	5.33
3523	1287	C	A	P	P	20.44
3523	1287	C	T	P	P	0.44
3532	1278	T	C	F	F	13.33
3562	1248	C	T	F	F	94.67
3570	1240	A	G	I	V	4.89
3577	1233	G	T	L	F	0.89
3579	1231	T	C	L	L	16.0
3580	1230	T	C	F	F	15.11
3589	1221	A	T	I	I	5.33
3592	1218	A	T	V	V	100.0
3612	1198	C	T	L	L	12.44
3619	1191	C	T	H	H	5.33
3622	1188	G	A	L	L	3.56
3628	1182	T	C	A	A	15.56
3634	1176	T	A	G	G	15.11
3634	1176	T	G	G	G	5.33
3649	1161	A	G	E	E	0.44
3652	1158	A	G	L	L	12.44
3654	1156	C	T	L	L	65.78
3670	1140	T	C	A	A	0.44
3673	1137	A	G	G	G	65.78

3684	1126	T	A	L	I	0.44
3709	1101	C	T	F	F	95.11
3715	1095	A	C	E	D	14.67
3733	1077	G	A	R	R	89.78
3736	1074	C	T	T	T	64.89
3745	1065	T	C	L	L	14.22
3749	1061	C	G	T	S	65.78
3757	1053	A	G	R	R	14.22
3763	1047	A	G	K	K	5.33
3767	1043	C	G	A	G	0.44
3772	1038	T	G	V	V	14.67
3781	1029	A	G	V	V	14.67
3790	1020	G	A	Q	Q	4.0
3826	984	T	A	I	I	9.78
3826	984	T	C	I	I	58.67
3829	981	T	C	I	I	12.89
3830	980	T	C	I	T	88.89
3831	979	A	G	I	V	0.44
3844	966	C	T	H	H	94.22
3859	951	G	A	K	K	12.89
3868	942	T	C	Y	Y	17.33
3871	939	T	C	P	P	11.56
3874	936	G	A	Q	Q	100.0
3886	924	G	A	Q	Q	21.78
3904	906	G	A	E	E	12.0
3906	904	G	C	E	Q	0.44
3916	894	A	G	Q	Q	14.22
3919	891	T	C	F	F	0.89
3924	886	G	C	E	Q	0.89
3934	876	T	A	G	G	5.33
3937	873	A	G	L	L	32.0
3941	869	T	A	L	*	0.44
3942	868	T	A	L	I	1.33
3942	868	T	C	L	L	8.44
3945	865	T	C	L	L	5.33
3949	861	G	C	V	V	5.33
3955	855	G	A	E	E	8.44
3967	843	G	A	E	E	73.78
3970	840	A	G	V	V	0.89
3979	831	G	A	E	E	15.56
3985	825	T	A	I	I	12.89

3997	813	T	C	S	S	19.56
4000	810	G	C	V	V	65.33
4006	804	G	A	K	K	0.44
4021	789	G	A	E	E	11.11
4024	786	A	G	E	E	15.11
4031	779	G	A	S	N	94.22
4036	774	T	C	D	D	5.33
4045	765	G	A	K	K	0.44
4050	760	T	C	L	L	16.89
4059	751	C	A	Q	K	4.44
4063	747	C	T	S	S	11.56
4069	741	A	G	E	E	14.22
4075	735	T	A	T	T	94.22
4076	734	C	T	T	I	6.67
4080	730	C	T	H	Y	1.78
4081	729	C	T	Y	Y	2.67
4087	723	T	A	D	E	1.78
4087	723	T	G	D	E	4.44
4090	720	A	G	R	R	5.78
4099	711	C	T	A	A	1.78
4105	705	T	C	Y	Y	17.78
4108	702	A	G	L	L	2.22
4111	699	A	G	L	L	0.44
4153	657	C	T	T	T	19.56
4174	636	T	C	N	N	5.78
4203	607	C	A	H	N	0.44
4213	597	T	C	F	F	6.67
4216	594	C	T	F	F	0.44
4219	591	A	T	I	I	0.89
4231	579	G	T	G	G	19.56
4252	558	T	C	I	I	5.33
4270	540	A	G	K	K	5.33
4293	517	C	T	L	L	4.0
4298	512	A	T	Q	L	14.22
4299	511	C	T	Q	*	14.22
4299	511	C	G	Q	E	0.89
4315	495	T	C	T	T	6.22
4330	480	C	T	C	C	3.56
4336	474	T	C	L	L	28.44
4339	471	C	A	T	T	4.0
4339	471	C	T	T	T	7.11

	4363	447	T	C	S	S	0.89
	4380	430	C	T	L	L	0.44
	4385	425	C	T	S	F	0.89
	4385	425	C	G	S	C	0.44
	4387	423	C	T	P	P	88.44
	4411	399	G	A	K	K	0.44
	4417	393	A	G	K	K	12.89
	4423	387	A	G	K	K	83.56
	4427	383	C	A	P	H	13.78
	4435	375	A	C	S	S	12.89
	4435	375	A	T	S	S	0.89
	4436	374	C	G	S	*	0.44
	4444	366	T	C	S	S	17.78
	4457	353	C	A	A	E	0.44
	4459	351	A	G	E	E	12.44
	4462	348	A	G	E	E	12.44
	4471	339	C	A	A	A	0.44
	4471	339	C	T	A	A	0.44
	4473	337	G	A	A	T	0.44
	4483	327	A	G	E	E	4.89
	4488	322	C	A	H	N	1.33
	4492	318	T	C	F	F	17.33
	4498	312	T	C	D	D	5.78
	4517	293	A	T	N	I	0.44
	4522	288	C	T	S	S	0.44
	4525	285	T	C	S	S	18.22
	4525	285	T	G	S	R	12.0
	4534	276	C	T	S	S	26.67
	4545	265	G	C	E	Q	0.44
	4564	246	G	A	V	V	0.44
**LTA intron**	4570		T	C			0.44
	4575		A	C			68.44
	4580		A	T			0.44
	4589		G	A			2.22
	4595		T	C			0.44
	4597		T	A			92.00
	4598		A	T			1.33
	4606		A	C			8.44
	4606		A	T			8.00
	4613		A	C			4.00
	4620		T	G			12.44

	4625		G	A			18.22
	4626		T	G			67.56
**LTA intron / stA**	4653	- / 501	C	G	- / F	- / L	0.44
	4662	- / 492	A	G	- / E	- / E	12.44
	4664	- / 490	G	C	- / E	- / Q	0.44
	4666	- / 488	G	A	- / G	- / E	1.33
	4677	- / 477	C	T	- / V	- / V	1.78
	4692	- / 462	T	C	- / T	- / T	4.89
	4704	- / 450	A	C	- / L	- / L	4.89
	4715	- / 439	G	T	- / G	- / C	0.44
	4724	- / 430	C	G	- / Q	- / E	5.78
	4726	- / 428	C	G	- / T	- / R	5.33
	4734	- / 420	C	T	- / D	- / D	4.89
	4764	- / 390	C	A	- / P	- / P	4.89
	4791	- / 363	A	T	- / L	- / F	0.44
	4797	- / 357	G	A	- / R	- / R	12.44
	4806	- / 348	T	A	- / L	- / L	16.89
	4809	- / 345	G	A	- / Q	- / Q	0.44
	4815	- / 339	A	G	- / L	- / L	12.0
	4830	- / 324	C	T	- / H	- / H	4.89
	4836	- / 318	T	A	- / S	- / S	17.33
	4854	- / 300	T	C	- / I	- / I	0.89
	4863	- / 291	A	T	- / E	- / D	13.33
	4877	- / 277	C	T	- / L	- / L	4.89
	4878	- / 276	C	T	- / T	- / T	4.89
	4894	- / 260	C	A	- / P	- / H	0.44
**LTA / stA**	4915	239 / 239	C	T	S / S	L / L	0.44
	4921	233 / 233	G	A	S / S	N / N	4.89
	4944	210 / 210	T	C	H / H	H / H	13.33
	4962	192 / 192	G	A	Q / Q	Q / Q	67.56
	4980	174 / 174	G	A	L / L	L / L	99.56
	5036	118 / 118	G	C	E / E	Q / Q	0.89
	5046	108 / 108	G	A	R / R	R / R	17.33
	5047	107 / 107	G	A	R / R	K / K	4.89
	5055	99 / 99	T	C	A / A	A / A	94.67
	5061	93 / 93	A	G	R / R	R / R	12.44
	5069	85 / 85	T	C	L / L	L / L	2.67
	5072	82 / 82	C	A	P / P	T / T	0.44
	5076	78 / 78	T	C	N / N	N / N	92.44
	5097	57 / 57	T	A	L / L	L / L	10.67
	5103	51 / 51	A	G	L / L	L / L	61.33

5115	39 / 39	C	G	L / L	L / L	0.89
5125	29 / 29	C	A	S / S	Y / Y	0.44
5127	27 / 27	A	G	E / E	E / E	0.89
5130	24 / 24	A	G	E / E	E / E	16.44
5139	15 / 15	T	A	L / L	L / L	11.56
5142	12/12	T	G	V / V	V / V	90.22
