# Supplementary material for: An unusually high substitution rate in transplant-associated BK polyomavirus in vivo is further concentrated in HLA-C-bound viral peptides
Source: PLoS Pathog. 2018 Oct 18;14(10):e1007368. doi: 10.1371/journal.ppat.1007368 (PMC6207329; doi:10.1371/journal.ppat.1007368)
Supplement: S3 Table — The positions, locus, reference and polymorphism, and percentage of samples with the polymorphism are shown. The genomic position and the reference base according to the BKV Dunlop reference strain. (PDF) [file ppat.1007368.s005.pdf]

Locus	Genomic position	Gene position	Reference base	Sample base	Percentage of samples
**Intergenic**	32		-	C	42.67
	261		-	T	0.44
	262		-	TC	0.44
	374		-	T	16.00
**Agnoprotein**	515-523	128-136	AAGACAGTG	-	0.44
	548-556	161-169	CTGCTTTAC[1]	-	10.61
**Intergenic**	598		C	-	3.56
	605		-	G	1.33
	2655		-	A	17.78
	2680		-	GC	19.11
	2708		C	-	1.78
**LTA**	2799-2804	2011-2006	CTGACC[2]	-	3.56
**Intron LTA**	4579		-	A	3.11
	4579		-	AA	5.78
	4579		-	C	8.89
	4579		-	CA	11.56

[1] Previously reported in GenBank accession numbers: EF376992, AB301101, AB263920, AB269868, AB263920, AB263920, AB263920, AB269845, AB269841, AB269838, AB269834, AB269826, AB263925, AB211388, AB211387, AB365150, AB365149, AB365141, AB365138, AB269839, AB269862, AB269835, AB211373.
[2] Previously reported in GenBank accession numbers: M23122, EF376992, AB301101, AB263920, AB211386, AB263916, AB365139, AB365130, JN192440.
