# Supplementary material for: An unusually high substitution rate in transplant-associated BK polyomavirus in vivo is further concentrated in HLA-C-bound viral peptides
Source: PLoS Pathog. 2018 Oct 18;14(10):e1007368. doi: 10.1371/journal.ppat.1007368 (PMC6207329; doi:10.1371/journal.ppat.1007368)
Supplement: S4 Table — Summary of interpatient evolutionary rate estimates (substitutions/site/year, s/s/y) of BKV using different molecular clock (strict, relaxed log-normal uncorrelated and relaxed exponential uncorrelated) and demography (constant size and Bayesian skyline) models. Median and 95% high-density interval (HDI) intervals are shown. Estimates were obtained after two independent runs of 30 million generations each with a 10% burn-in. Convergence of the runs (ESS > 200) was checked with Tracer. (PDF) [file ppat.1007368.s006.pdf]

Model	Median	95% HDI
Strict Constant	6.25×10^{-5}	[6.85×10^{-6} – 1.32×10^{-4}]
Relaxed Log-Normal Constant	1.51×10^{-4}	[1.71×10^{-5} – 3.43×10^{-4}]
Relaxed Exponential Constant	1.82×10^{-4}	[1.06×10^{-5}– 4.24×10^{-4}]
Strict Bayesian Skyline	4.80×10^{-5}	[7.11×10^{-6}– 1.05×10^{-4}]
Relaxed Log-Normal Bayesian Skyline	9.64×10^{-5}	[1.00×10^{-5} – 2.15×10^{-4}]
Relaxed Exponential Bayesian Skyline	1.09×10^{-4}	[1.12×10^{-5} – 2.70×10^{-4}]
