# Supplementary material for: An unusually high substitution rate in transplant-associated BK polyomavirus in vivo is further concentrated in HLA-C-bound viral peptides
Source: PLoS Pathog. 2018 Oct 18;14(10):e1007368. doi: 10.1371/journal.ppat.1007368 (PMC6207329; doi:10.1371/journal.ppat.1007368)
Supplement: S5 Table — Alleles are shown for HLA-A, -B and -C at the 2nd field of resolution for donors and recipients. (PDF) [file ppat.1007368.s007.pdf]

HLA-A	Donors	Recipients
HLA-A*01:01	0.190	0.260
HLA-A*01:02		0.014
HLA-A*02:01	0.483	0.466
HLA-A*02:05	0.034	
HLA-A*02:06	0.017	0.027
HLA-A*03:01	0.241	0.247
HLA-A*11:01	0.069	0.110
HLA-A*23:01	0.052	0.068
HLA-A*24:01		0.014
HLA-A*24:02	0.207	0.205
HLA-A*24:02L		0.014
HLA-A*25:01		0.041
HLA-A*26:01	0.052	0.041
HLA-A*29:01		0.041
HLA-A*29:02	0.103	0.068
HLA-A*30:01	0.017	
HLA-A*30:02	0.052	0.041
HLA-A*30:04	0.017	
HLA-A*31:01	0.052	0.027
HLA-A*32:01	0.069	0.055
HLA-A*33:01	0.052	0.014
HLA-A*33:03	0.017	0.014
HLA-A*36:01		0.014
HLA-A*39:01	0.017	
HLA-A*43:01		0.014
HLA-A*66:01	0.017	
HLA-A*68:01	0.069	0.041
HLA-A*68:02	0.034	
HLA-A*80:01		0.014

HLA-B	Donors	Recipients
HLA-B*07:02	0.241	0.192
HLA-B*07:05		0.014
HLA-B*08:01	0.103	0.205
HLA-B*13:02	0.052	0.014
HLA-B*14:01		0.014
HLA-B*14:02	0.103	0.041
HLA-B*15:01	0.121	0.096
HLA-B*15:17		0.014
HLA-B*15:18		0.014
HLA-B*15:24		0.014
HLA-B*18:01	0.155	0.164
HLA-B*27:02	0.034	0.014
HLA-B*27:05	0.103	0.041
HLA-B*35:01	0.086	0.110
HLA-B*35:02		0.027
HLA-B*35:03	0.034	0.041
HLA-B*35:08	0.017	
HLA-B*37:01		0.027
HLA-B*38:01	0.052	
HLA-B*39:01	0.052	0.041
HLA-B*40:01	0.052	0.068
HLA-B*40:02	0.034	0.055
HLA-B*41:01		0.014
HLA-B*41:02	0.017	
HLA-B*44:01		0.014
HLA-B*44:02	0.138	0.151
HLA-B*44:03	0.069	0.082
HLA-B*44:05		0.014
HLA-B*47:01	0.017	
HLA-B*47:02	0.017	
HLA-B*48:01		0.014
HLA-B*49:01	0.086	0.041
HLA-B*50:01	0.034	0.068
HLA-B*51:01	0.121	0.164

HLA-B*51:08	0.017	
HLA-B*53:01	0.014	
HLA-B*52:01		0.014
HLA-B*54:01		0.014
HLA-B*55:01	0.034	
HLA-B*56:01	0.017	0.027
HLA-B*57:01	0.069	0.041
HLA-B*58:01	0.017	0.041

HLA-C	Donors	Recipients
HLA-C*01:02	0.121	0.055
HLA-C*02:02	0.138	0.110
HLA-C*02:07		0.014
HLA-C*02:10	0.017	
HLA-C*03:02	0.017	0.014
HLA-C*03:03	0.138	0.082
HLA-C*03:04	0.052	0.068
HLA-C*04:01	0.138	0.205
HLA-C*05:01	0.172	0.164
HLA-C*06:02	0.155	0.151
HLA-C*07:01	0.276	0.370
HLA-C*07:02	0.224	0.233
HLA-C*07:04	0.034	0.027
HLA-C*08:02	0.103	0.055
HLA-C*08:03		0.014
HLA-C*12:02		0.014
HLA-C*12:03	0.121	0.041
HLA-C*14:02	0.017	0.082
HLA-C*15:02	0.052	0.068
HLA-C*16:01	0.052	0.041
HLA-C*16:02	0.017	0.041
HLA-C*17:01		0.014
HLA-C*17:03	0.017	0.027
