# Supplementary material for: An unusually high substitution rate in transplant-associated BK polyomavirus in vivo is further concentrated in HLA-C-bound viral peptides
Source: PLoS Pathog. 2018 Oct 18;14(10):e1007368. doi: 10.1371/journal.ppat.1007368 (PMC6207329; doi:10.1371/journal.ppat.1007368)
Supplement: S6 Table — BK polyomavirus predicted epitopes presented by HLA-A, -B and -C by protein. Agnoprotein, VP1-3, large T antigen “LTA” and small t antigen “stA” predicted peptides presented by HLA-A, -B and -C from the BK polyomavirus Dunlop reference strain are listed. The starting and ending amino acid of the protein, length of the peptide, peptide sequence, and HLA allele that can present peptide are shown. The IC50 for each peptide and specific HLA allele are also included. (PDF) [file ppat.1007368.s008.pdf]

Protein	Aa start	Aa end	Length (aa)	Peptide	HLA Allele x_1	ic50 (nM) x_1	HLA Allele x_2	ic50 (nM) x_2	HLA Allele x_3	ic50 (nM) x_3	HLA Allele x_4	ic50 (nM) x_4	HLA Allele x_5	ic50 (nM) x_5
Agnoprotein	1	8	8	MVLRQLSR	HLA-A*31:01	39.00	HLA-A*33:01	49.00						
Agnoprotein	1	12	12	MVLRQLSRQASV	HLA-A*02:06	13.00	HLA-A*68:02	46.00						
Agnoprotein	1	13	13	MVLRQLSRQASVK	HLA-A*03:01	32.00	HLA-A*11:01	35.00						
Agnoprotein	1	14	14	MVLRQLSRQASVKV	HLA-A*02:06	18.00								
Agnoprotein	4	12	9	RQLSRQASV	HLA-A*02:06	17.00								
Agnoprotein	4	13	10	RQLSRQASVK	HLA-A*03:01	49.00								
Agnoprotein	4	16	13	RQLSRQASVKVGK	HLA-A*31:01	33.00	HLA-B*27:05	48.00						
Agnoprotein	6	13	8	LSRQASVK	HLA-A*30:01	18.00								
Agnoprotein	6	16	11	LSRQASVKVGK	HLA-A*30:01	16.00								
Agnoprotein	6	18	13	LSRQASVKVGKTW	HLA-B*57:01	15.00	HLA-B*58:01	24.00						
Agnoprotein	8	16	9	RQASVKVGK	HLA-A*31:01	48.00								
Agnoprotein	8	18	11	RQASVKVGKTW	HLA-B*58:01	44.00								
Agnoprotein	9	18	10	QASVKVGKTW	HLA-B*58:01	17.00								
Agnoprotein	10	18	9	ASVKVGKTW	HLA-B*57:01	17.00	HLA-B*58:01	11.00						
Agnoprotein	11	22	12	SVKVGKTWTGTK	HLA-A*30:01	19.00								
Agnoprotein	11	23	13	SVKVGKTWTGTKK	HLA-A*30:01	45.00								
Agnoprotein	14	27	14	VGKTWTGTKKRAQR	HLA-A*31:01	47.00								
Agnoprotein	16	23	8	KTWTGTKK	HLA-A*11:01	30.00								
Agnoprotein	16	24	9	KTWTGTKKR	HLA-A*31:01	41.00								
Agnoprotein	16	27	12	KTWTGTKKRAQR	HLA-A*31:01	11.00								
Agnoprotein	16	28	13	KTWTGTKKRAQRI	HLA-A*32:01	31.00								
Agnoprotein	16	29	14	KTWTGTKKRAQRIF	HLA-A*32:01	12.00	HLA-B*57:01	39.00	HLA-B*58:01	39.00				
Agnoprotein	20	27	8	GTKKRAQR	HLA-A*31:01	16.00								
Agnoprotein	20	30	11	GTKKRAQRIFI	HLA-A*30:01	16.00								
Agnoprotein	20	32	13	GTKKRAQRIFIFI	HLA-A*30:01	17.00								
Agnoprotein	22	30	9	KKRAQRIFI	HLA-A*30:01	8.00								
Agnoprotein	22	32	11	KKRAQRIFIFI	HLA-A*30:01	10.00								
Agnoprotein	23	31	9	KRAQRIFIF	HLA-B*27:05	40.00								
Agnoprotein	23	32	10	KRAQRIFIFI	HLA-B*27:05	26.00								
Agnoprotein	23	35	13	KRAQRIFIFILEL	HLA-B*27:05	35.00								

Agnoprotein	23	36	14	KRAQRIFIFILELL	HLA-B*27:05	34.00				
Agnoprotein	24	31	8	RAQRIFIF	HLA-B*58:01	41.00				
Agnoprotein	24	32	9	RAQRIFIFI	HLA-A*30:01	39.00	HLA-C*12:03	46.00		
Agnoprotein	25	32	8	AQRIFIFI	HLA-A*30:01	19.00				
Agnoprotein	26	33	8	QRIFIFIL	HLA-B*39:01	27.00				
Agnoprotein	26	35	10	QRIFIFILEL	HLA-B*27:05	42.00				
Agnoprotein	26	37	12	QRIFIFILELLL	HLA-B*27:05	46.00				
Agnoprotein	27	39	13	RIFIFILELLLEF	HLA-A*32:01	12.00				
Agnoprotein	28	36	9	IFIFILELL	HLA-A*23:01	41.00				
Agnoprotein	28	39	12	IFIFILELLLEF	HLA-A*23:01	13.00	HLA-A*29:02	21.00		
Agnoprotein	28	41	14	IFIFILELLLEFCR	HLA-A*31:01	27.00	HLA-A*33:01	14.00	HLA-A*68:01	16.00
Agnoprotein	29	41	13	FIFILELLLEFCR	HLA-A*33:01	33.00	HLA-A*68:01	7.00		
Agnoprotein	30	41	12	IFILELLLEFCR	HLA-A*33:01	34.00				
Agnoprotein	31	39	9	FILELLLEF	HLA-A*02:06	12.00	HLA-A*29:02	48.00		
Agnoprotein	31	41	11	FILELLLEFCR	HLA-A*33:01	40.00	HLA-A*68:01	44.00		
Agnoprotein	33	46	14	LELLLEFCRGEDSV	HLA-B*40:02	40.00				
Agnoprotein	34	41	8	ELLLEFCR	HLA-A*33:01	15.00				
Agnoprotein	35	46	12	LLLEFCRGEDSV	HLA-A*02:01	12.00	HLA-A*02:06	9.00		
Agnoprotein	44	56	13	DSVDGKNKSTTAL	HLA-C*03:03	50.00				
Agnoprotein	49	60	12	KNKSTTALPAVK	HLA-A*30:01	38.00				
Agnoprotein	52	59	8	STTALPAV	HLA-A*02:06	44.00	HLA-A*68:02	29.00		
Agnoprotein	52	60	9	STTALPAVK	HLA-A*11:01	29.00				
Agnoprotein	52	63	12	STTALPAVKDSV	HLA-A*68:02	12.00				
Agnoprotein	53	60	8	TTALPAVK	HLA-A*11:01	33.00	HLA-A*68:01	19.00		
Agnoprotein	53	63	11	TTALPAVKDSV	HLA-A*68:02	5.00				
Agnoprotein	53	64	12	TTALPAVKDSVK	HLA-A*68:01	20.00				
Agnoprotein	54	63	10	TALPAVKDSV	HLA-C*03:03	47.00	HLA-C*12:03	28.00		
VP1	2	14	13	APTKRKGECPGAA	HLA-B*07:02	27.00				
VP1	9	22	14	ECPGAAPKKPKEPV	HLA-A*68:02	29.00				
VP1	12	22	11	GAAPKKPKEPV	HLA-C*03:03	40.00				
VP1	14	22	9	APKKPKEPV	HLA-B*07:02	22.00				

VP1	22	29	8	VQVPKLLI	HLA-A*02:06	24.00						
VP1	22	33	12	VQVPKLLIKGGV	HLA-A*02:06	24.00						
VP1	22	35	14	VQVPKLLIKGGVEV	HLA-A*02:06	7.00						
VP1	27	35	9	LLIKGGVEV	HLA-A*02:01	25.00	HLA-A*02:06	15.00				
VP1	27	36	10	LLIKGGVEVL	HLA-C*03:03	14.00						
VP1	27	38	12	LLIKGGVEVLEV	HLA-A*02:01	16.00	HLA-A*02:06	18.00				
VP1	34	42	9	EVLEVKTGV	HLA-A*68:02	7.00						
VP1	34	45	12	EVLEVKTGVDAI	HLA-A*68:02	28.00						
VP1	37	48	12	EVKTGVDAITEV	HLA-A*68:02	23.00						
VP1	40	52	13	TGVDAITEVECFL	HLA-C*03:03	21.00						
VP1	41	48	8	GVDAITEV	HLA-A*02:06	29.00						
VP1	43	52	10	DAITEVECFL	HLA-C*03:03	38.00						
VP1	46	56	11	TEVECFLNPEM	HLA-B*40:02	22.00						
VP1	48	56	9	VECFLNPEM	HLA-B*40:02	33.00						
VP1	56	66	11	MGDPDENLRGF	HLA-C*05:01	35.00						
VP1	60	68	9	DENLRGFSL	HLA-B*18:01	26.00						
VP1	62	69	8	NLRGFSLK	HLA-A*03:01	47.00	HLA-A*30:01	47.00				
VP1	70	83	14	LSAENDFSSDSPER	HLA-A*68:01	35.00						
VP1	72	85	14	AENDFSSDSPERKM	HLA-B*44:02	34.00						
VP1	76	83	8	FSSDSPER	HLA-A*68:01	19.00						
VP1	76	85	10	FSSDSPERKM	HLA-C*03:03	7.00	HLA-C*12:03	34.00				
VP1	76	86	11	FSSDSPERKML	HLA-C*03:03	8.00	HLA-C*12:03	17.00				
VP1	76	89	14	FSSDSPERKMLPCY	HLA-A*01:01	20.00	HLA-B*35:01	24.00				
VP1	77	89	13	SSDSPERKMLPCY	HLA-C*05:01	42.00						
VP1	84	92	9	KMLPCYSTA	HLA-A*02:01	22.00	HLA-A*02:06	14.00				
VP1	84	93	10	KMLPCYSTAR	HLA-A*31:01	4.00						
VP1	84	94	11	KMLPCYSTARI	HLA-A*02:01	46.00						
VP1	84	96	13	KMLPCYSTARIPL	HLA-A*02:01	9.00	HLA-A*02:06	9.00	HLA-A*32:01	33.00	HLA-C*14:02	22.00
VP1	85	94	10	MLPCYSTARI	HLA-C*14:02	34.00						
VP1	85	96	12	MLPCYSTARIPL	HLA-A*02:01	18.00	HLA-A*02:06	36.00	HLA-C*14:02	12.00		
VP1	86	96	11	LPCYSTARIPL	HLA-B*07:02	27.00	HLA-B*35:01	45.00				

VP1	88	96	9	CYSTARIPL	HLA-C*14:02	7.00				
VP1	88	99	12	CYSTARIPLPNL	HLA-C*14:02	30.00				
VP1	89	96	8	YSTARIPL	HLA-C*03:03	6.00	HLA-C*14:02	34.00		
VP1	89	99	11	YSTARIPLPNL	HLA-C*03:03	22.00				
VP1	100	111	12	NEDLTCGNLLMW	HLA-B*44:02	40.00				
VP1	103	111	9	LTCGNLLMW	HLA-B*57:01	33.00	HLA-B*58:01	22.00		
VP1	107	114	8	NLLMWEAV	HLA-A*02:06	24.00				
VP1	107	116	10	NLLMWEAVTV	HLA-A*02:01	25.00				
VP1	107	120	14	NLLMWEAVTVQTEV	HLA-A*02:01	15.00	HLA-A*02:06	49.00		
VP1	108	116	9	LLMWEAVTV	HLA-A*02:01	9.00	HLA-A*02:06	14.00		
VP1	108	120	13	LLMWEAVTVQTEV	HLA-A*02:01	6.00	HLA-A*02:06	10.00		
VP1	108	121	14	LLMWEAVTVQTEVI	HLA-A*02:01	49.00				
VP1	109	116	8	LMWEAVTV	HLA-A*02:01	47.00	HLA-A*02:06	5.00	HLA-C*12:03	29.00
VP1	109	120	12	LMWEAVTVQTEV	HLA-A*02:01	4.00	HLA-A*02:06	4.00		
VP1	109	121	13	LMWEAVTVQTEVI	HLA-A*02:01	19.00	HLA-C*12:03	47.00		
VP1	111	121	11	WEAVTVQTEVI	HLA-B*40:01	34.00				
VP1	111	123	13	WEAVTVQTEVIGI	HLA-B*40:01	36.00				
VP1	112	120	9	EAVTVQTEV	HLA-A*68:02	7.00				
VP1	112	123	12	EAVTVQTEVIGI	HLA-A*68:02	20.00				
VP1	114	126	13	VTVQTEVIGITSM	HLA-C*03:03	39.00				
VP1	114	127	14	VTVQTEVIGITSML	HLA-C*03:03	32.00				
VP1	116	123	8	VQTEVIGI	HLA-A*02:06	15.00				
VP1	116	127	12	VQTEVIGITSML	HLA-A*02:06	25.00				
VP1	116	129	14	VQTEVIGITSMLNL	HLA-A*02:06	39.00				
VP1	118	127	10	TEVIGITSML	HLA-B*40:01	36.00				
VP1	118	129	12	TEVIGITSMLNL	HLA-B*40:01	19.00				
VP1	119	126	8	EVIGITSM	HLA-A*25:01	23.00	HLA-A*26:01	3.00	HLA-A*68:02	17.00
VP1	119	127	9	EVIGITSML	HLA-A*26:01	4.00	HLA-A*68:02	3.00		
VP1	119	129	11	EVIGITSMLNL	HLA-A*26:01	10.00	HLA-A*68:02	7.00		
VP1	119	130	12	EVIGITSMLNLH	HLA-A*26:01	15.00				
VP1	119	131	13	EVIGITSMLNLHA	HLA-A*68:02	8.00				

VP1	123	135	13	ITSMLNLHAGSQK	HLA-A*11:01	22.00	HLA-A*68:01	33.00						
VP1	124	135	12	TSMLNLHAGSQK	HLA-A*11:01	17.00	HLA-A*68:01	45.00						
VP1	124	136	13	TSMLNLHAGSQKV	HLA-C*15:02	37.00								
VP1	125	136	12	SMLNLHAGSQKV	HLA-A*02:01	19.00	HLA-A*02:06	17.00						
VP1	126	135	10	MLNLHAGSQK	HLA-A*03:01	20.00								
VP1	135	143	9	KVHEHGGGK	HLA-A*30:01	4.00								
VP1	135	145	11	KVHEHGGGKPI	HLA-A*30:01	23.00	HLA-C*12:03	41.00	HLA-C*14:02	29.00				
VP1	137	145	9	HEHGGGKPI	HLA-B*40:01	12.00	HLA-B*40:02	21.00						
VP1	143	155	13	KPIQGSNFHFFAV	HLA-B*07:02	17.00								
VP1	145	152	8	IQGSNFHF	HLA-B*15:01	33.00								
VP1	145	153	9	IQGSNFHFF	HLA-A*23:01	34.00	HLA-A*24:02	32.00	HLA-B*15:01	34.00				
VP1	147	155	9	GSNFHFFAV	HLA-A*02:06	43.00								
VP1	148	155	8	SNFHFFAV	HLA-A*68:02	48.00								
VP1	149	160	12	NFHFFAVGGEPL	HLA-C*14:02	18.00								
VP1	150	160	11	FHFFAVGGEPL	HLA-B*39:01	12.00	HLA-C*03:03	16.00	HLA-C*14:02	9.00				
VP1	150	162	13	FHFFAVGGEPLEM	HLA-B*39:01	42.00	HLA-C*06:02	25.00	HLA-C*07:01	13.00	HLA-C*14:02	30.00		
VP1	151	160	10	HFFAVGGEPL	HLA-C*14:02	6.00								
VP1	151	162	12	HFFAVGGEPLEM	HLA-C*14:02	20.00								
VP1	152	160	9	FFAVGGEPL	HLA-C*03:03	5.00	HLA-C*14:02	7.00						
VP1	152	162	11	FFAVGGEPLEM	HLA-C*03:03	40.00	HLA-C*14:02	35.00						
VP1	153	160	8	FAVGGEPL	HLA-A*02:06	36.00	HLA-B*35:01	29.00	HLA-C*03:03	1.00	HLA-C*12:03	17.00		
VP1	153	162	10	FAVGGEPLEM	HLA-B*35:01	46.00	HLA-C*03:03	2.00	HLA-C*12:03	24.00				
VP1	153	163	11	FAVGGEPLEMQ	HLA-C*03:03	25.00								
VP1	153	165	13	FAVGGEPLEMQGV	HLA-A*02:06	10.00	HLA-C*03:03	3.00	HLA-C*12:03	37.00				
VP1	153	166	14	FAVGGEPLEMQGVL	HLA-C*03:03	1.00	HLA-C*12:03	22.00						
VP1	158	169	12	EPLEMQGVLMNY	HLA-B*35:01	31.00								
VP1	158	170	13	EPLEMQGVLMNYR	HLA-A*33:01	27.00								
VP1	160	167	8	LEMQGVLM	HLA-B*40:01	39.00								
VP1	160	169	10	LEMQGVLMNY	HLA-B*15:01	23.00	HLA-B*18:01	24.00						
VP1	160	173	14	LEMQGVLMNYRSKY	HLA-B*18:01	14.00	HLA-B*44:02	33.00	HLA-B*44:03	38.00				
VP1	161	170	10	EMQGVLMNYR	HLA-A*31:01	42.00	HLA-A*33:01	12.00	HLA-A*68:01	24.00				

VP1	162	170	9	MQGVLMNYR	HLA-A*31:01	11.00								
VP1	162	173	12	MQGVLMNYRSKY	HLA-A*30:02	28.00								
VP1	164	172	9	GVLMNYRSK	HLA-A*30:01	32.00								
VP1	165	172	8	VLMNYRSK	HLA-A*03:01	25.00	HLA-A*11:01	29.00						
VP1	165	173	9	VLMNYRSKY	HLA-A*29:02	49.00	HLA-A*30:02	19.00						
VP1	166	173	8	LMNYRSKY	HLA-A*29:02	34.00	HLA-A*30:02	48.00						
VP1	168	178	11	NYRSKYPDGTI	HLA-C*14:02	28.00								
VP1	168	181	14	NYRSKYPDGTITPK	HLA-A*30:01	30.00								
VP1	170	181	12	RSKYPDGTITPK	HLA-A*30:01	4.00	HLA-A*31:01	27.00						
VP1	179	190	12	TPKNPTAQSQVM	HLA-B*07:02	44.00								
VP1	182	190	9	NPTAQSQVM	HLA-B*07:02	50.00	HLA-B*35:01	31.00						
VP1	185	197	13	AQSQVMNTDHKAY	HLA-B*15:01	36.00								
VP1	185	198	14	AQSQVMNTDHKAYL	HLA-A*02:06	29.00								
VP1	187	197	11	SQVMNTDHKAY	HLA-B*15:01	38.00								
VP1	187	198	12	SQVMNTDHKAYL	HLA-A*02:06	15.00								
VP1	188	195	8	QVMNTDHK	HLA-A*11:01	42.00	HLA-A*68:01	50.00						
VP1	188	197	10	QVMNTDHKAY	HLA-B*15:01	28.00								
VP1	188	198	11	QVMNTDHKAYL	HLA-A*68:02	35.00	HLA-C*14:02	37.00						
VP1	188	200	13	QVMNTDHKAYLDK	HLA-A*11:01	37.00								
VP1	191	198	8	NTDHKAYL	HLA-C*05:01	34.00								
VP1	191	204	14	NTDHKAYLDKNNAY	HLA-A*01:01	7.00	HLA-C*05:01	26.00						
VP1	195	204	10	KAYLDKNNAY	HLA-C*12:03	34.00								
VP1	195	206	12	KAYLDKNNAYPV	HLA-C*12:03	18.00	HLA-C*15:02	32.00						
VP1	196	204	9	AYLDKNNAY	HLA-C*14:02	8.00								
VP1	196	206	11	AYLDKNNAYPV	HLA-C*14:02	6.00								
VP1	197	204	8	YLDKNNAY	HLA-A*01:01	9.00	HLA-C*05:01	3.00						
VP1	197	205	9	YLDKNNAYP	HLA-C*05:01	10.00								
VP1	197	206	10	YLDKNNAYPV	HLA-A*02:01	6.00	HLA-A*02:06	22.00	HLA-C*05:01	3.00	HLA-C*08:02	40.00	HLA-C*12:03	40.00
VP1	197	209	13	YLDKNNAYPVECW	HLA-C*05:01	6.00								
VP1	197	210	14	YLDKNNAYPVECWV	HLA-A*02:01	8.00	HLA-A*02:06	16.00	HLA-C*05:01	3.00				
VP1	202	209	8	NAYPVECW	HLA-B*53:01	24.00	HLA-B*58:01	17.00						

VP1	202	210	9	NAYPVECWV	HLA-C*12:03	36.00		
VP1	202	215	14	NAYPVECWVPDPSR	HLA-A*68:01	13.00		
VP1	204	212	9	YPVECWVPD	HLA-B*35:01	39.00		
VP1	204	214	11	YPVECWVPDPS	HLA-B*35:01	15.00		
VP1	210	222	13	VPDPSRNENARYF	HLA-C*05:01	11.00		
VP1	214	222	9	SRNENARYF	HLA-C*06:02	7.00	HLA-C*07:01	9.00
VP1	214	225	12	SRNENARYFGTF	HLA-C*06:02	50.00		
VP1	220	231	12	RYFGTFTGGENV	HLA-C*14:02	14.00		
VP1	221	234	14	YFGTFTGGENVPPV	HLA-C*14:02	27.00		
VP1	222	235	14	FGTFTGGENVPPVL	HLA-C*03:03	36.00		
VP1	224	234	11	TFTGGENVPPV	HLA-C*14:02	13.00		
VP1	224	235	12	TFTGGENVPPVL	HLA-C*14:02	30.00		
VP1	225	234	10	FTGGENVPPV	HLA-A*02:01	36.00	HLA-A*02:06	15.00
VP1	225	235	11	FTGGENVPPVL	HLA-C*03:03	37.00		
VP1	225	237	13	FTGGENVPPVLHV	HLA-A*02:06	35.00	HLA-C*12:03	44.00
VP1	228	235	8	GENVPPVL	HLA-B*40:01	13.00		
VP1	230	237	8	NVPPVLHV	HLA-A*02:06	27.00	HLA-A*68:02	12.00
VP1	230	241	12	NVPPVLHVTNTA	HLA-A*68:02	23.00		
VP1	235	245	11	LHVTNTATTVL	HLA-B*39:01	15.00		
VP1	235	246	12	LHVTNTATTVLL	HLA-B*39:01	25.00		
VP1	237	244	8	VTNTATTV	HLA-C*15:02	35.00		
VP1	239	246	8	NTATTVLL	HLA-A*68:02	11.00		
VP1	239	251	13	NTATTVLLDEQGV	HLA-A*68:02	14.00		
VP1	242	251	10	TTVLLDEQGV	HLA-A*68:02	36.00		
VP1	242	254	13	TTVLLDEQGVGPL	HLA-A*68:02	19.00	HLA-C*03:03	50.00
VP1	244	251	8	VLLDEQGV	HLA-A*02:06	14.00		
VP1	244	254	11	VLLDEQGVGPL	HLA-C*03:03	20.00		
VP1	245	254	10	LLDEQGVGPL	HLA-C*05:01	6.00	HLA-C*08:02	39.00
VP1	250	261	12	GVGPLCKADSLY	HLA-A*29:02	24.00		
VP1	256	267	12	KADSLYVSAADI	HLA-C*05:01	50.00		
VP1	259	267	9	SLYVSAADI	HLA-C*14:02	29.00		

VP1	259	270	12	SLYVSAADICGL	HLA-C*14:02	25.00		
VP1	260	267	8	LYVSAADI	HLA-C*14:02	16.00		
VP1	260	270	11	LYVSAADICGL	HLA-C*14:02	11.00		
VP1	260	271	12	LYVSAADICGLF	HLA-A*23:01	24.00	HLA-C*14:02	32.00
VP1	261	270	10	YVSAADICGL	HLA-A*02:06	40.00	HLA-C*03:03	14.00
VP1	261	271	11	YVSAADICGLF	HLA-B*35:01	18.00		
VP1	262	270	9	VSAADICGL	HLA-C*03:03	28.00		
VP1	263	270	8	SAADICGL	HLA-C*03:03	8.00		
VP1	264	271	8	AADICGLF	HLA-C*05:01	5.00		
VP1	268	280	13	CGLFTNSSGTQQW	HLA-B*58:01	34.00		
VP1	271	281	11	FTNSSGTQQWR	HLA-A*68:01	17.00		
VP1	271	283	13	FTNSSGTQQWRGL	HLA-C*03:03	38.00		
VP1	273	280	8	NSSGTQQW	HLA-B*58:01	12.00		
VP1	274	286	13	SSGTQQWRGLARY	HLA-A*30:02	31.00		
VP1	276	288	13	GTQQWRGLARYFK	HLA-A*11:01	18.00	HLA-A*31:01	50.00
VP1	277	286	10	TQQWRGLARY	HLA-B*15:01	34.00		
VP1	277	288	12	TQQWRGLARYFK	HLA-A*11:01	22.00		
VP1	278	289	12	QQWRGLARYFKI	HLA-A*02:06	29.00		
VP1	281	288	8	RGLARYFK	HLA-A*30:01	18.00	HLA-A*31:01	20.00
VP1	281	290	10	RGLARYFKIR	HLA-A*31:01	38.00		
VP1	281	292	12	RGLARYFKIRLR	HLA-A*31:01	17.00		
VP1	281	294	14	RGLARYFKIRLRKR	HLA-A*31:01	23.00		
VP1	282	293	12	GLARYFKIRLRK	HLA-A*03:01	31.00		
VP1	283	290	8	LARYFKIR	HLA-A*31:01	37.00		
VP1	283	292	10	LARYFKIRLR	HLA-A*31:01	19.00		
VP1	283	293	11	LARYFKIRLRK	HLA-A*30:01	14.00		
VP1	283	294	12	LARYFKIRLRKR	HLA-A*31:01	33.00		
VP1	284	292	9	ARYFKIRLR	HLA-B*27:05	35.00		
VP1	284	293	10	ARYFKIRLRK	HLA-B*27:05	38.00		
VP1	284	296	13	ARYFKIRLRKRSV	HLA-C*06:02	43.00	HLA-C*07:01	33.00
VP1	285	292	8	RYFKIRLR	HLA-A*31:01	5.00		

VP1	285	293	9	RYFKIRLRK	HLA-A*30:01	10.00	HLA-A*31:01	15.00		
VP1	285	294	10	RYFKIRLRKR	HLA-A*31:01	6.00				
VP1	285	296	12	RYFKIRLRKRSV	HLA-C*14:02	27.00				
VP1	285	297	13	RYFKIRLRKRSVK	HLA-A*30:01	12.00	HLA-A*31:01	11.00		
VP1	286	294	9	YFKIRLRKR	HLA-A*31:01	35.00	HLA-A*33:01	15.00		
VP1	286	296	11	YFKIRLRKRSV	HLA-B*08:01	24.00				
VP1	288	296	9	KIRLRKRSV	HLA-A*30:01	15.00	HLA-B*08:01	39.00		
VP1	288	297	10	KIRLRKRSVK	HLA-A*03:01	34.00				
VP1	288	300	13	KIRLRKRSVKNPY	HLA-A*30:01	17.00				
VP1	290	297	8	RLRKRSVK	HLA-A*03:01	24.00	HLA-A*30:01	4.00	HLA-A*31:01	44.00
VP1	290	300	11	RLRKRSVKNPY	HLA-A*30:01	20.00				
VP1	290	302	13	RLRKRSVKNPYPI	HLA-A*30:01	13.00	HLA-A*32:01	25.00	HLA-B*08:01	17.00
VP1	290	303	14	RLRKRSVKNPYPIS	HLA-A*30:01	26.00				
VP1	294	302	9	RSVKNPYPI	HLA-A*32:01	25.00	HLA-B*58:01	44.00	HLA-C*15:02	24.00
VP1	294	304	11	RSVKNPYPISF	HLA-B*58:01	15.00				
VP1	294	306	13	RSVKNPYPISFLL	HLA-B*58:01	26.00	HLA-C*15:02	30.00		
VP1	295	302	8	SVKNPYPI	HLA-A*30:01	28.00				
VP1	295	305	11	SVKNPYPISFL	HLA-A*30:01	19.00				
VP1	295	306	12	SVKNPYPISFLL	HLA-A*30:01	24.00				
VP1	298	305	8	NPYPISFL	HLA-C*12:03	28.00				
VP1	298	306	9	NPYPISFLL	HLA-B*35:01	40.00	HLA-B*39:01	34.00	HLA-B*53:01	17.00
VP1	298	310	13	NPYPISFLLSDLI	HLA-B*53:01	27.00	HLA-C*12:03	29.00		
VP1	299	306	8	PYPISFLL	HLA-C*14:02	36.00				
VP1	299	309	11	PYPISFLLSDL	HLA-C*14:02	22.00				
VP1	299	310	12	PYPISFLLSDLI	HLA-C*14:02	39.00				
VP1	300	309	10	YPISFLLSDL	HLA-B*35:01	34.00				
VP1	300	310	11	YPISFLLSDLI	HLA-B*53:01	13.00	HLA-C*12:03	29.00		
VP1	302	312	11	ISFLLSDLINR	HLA-A*68:01	21.00				
VP1	302	313	12	ISFLLSDLINRR	HLA-A*31:01	43.00	HLA-A*68:01	23.00		
VP1	304	317	14	FLLSDLINRRTQRV	HLA-A*02:01	4.00	HLA-A*02:06	4.00		
VP1	305	317	13	LLSDLINRRTQRV	HLA-A*02:01	16.00	HLA-A*02:06	36.00		

VP1	308	316	9	DLINRRTQR	HLA-A*33:01	15.00		
VP1	309	316	8	LINRRTQR	HLA-A*31:01	23.00		
VP1	312	325	14	RRTQRVDGQPMYGM	HLA-B*27:05	36.00		
VP1	313	322	10	RTQRVDGQPM	HLA-C*15:02	44.00		
VP1	313	323	11	RTQRVDGQPMY	HLA-A*30:02	35.00		
VP1	314	322	9	TQRVDGQPM	HLA-B*15:01	36.00		
VP1	316	323	8	RVDGQPMY	HLA-C*05:01	9.00		
VP1	316	325	10	RVDGQPMYGM	HLA-C*05:01	14.00		
VP1	316	329	14	RVDGQPMYGMESQV	HLA-C*05:01	11.00		
VP1	319	332	14	GQPMYGMESQVEEV	HLA-A*02:06	36.00		
VP1	322	329	8	MYGMESQV	HLA-C*14:02	28.00		
VP1	322	332	11	MYGMESQVEEV	HLA-C*14:02	10.00		
VP1	322	334	13	MYGMESQVEEVRV	HLA-C*14:02	33.00		
VP1	322	335	14	MYGMESQVEEVRVF	HLA-C*14:02	13.00		
VP1	323	332	10	YGMESQVEEV	HLA-C*12:03	42.00		
VP1	323	335	13	YGMESQVEEVRVF	HLA-B*15:01	34.00		
VP1	325	335	11	MESQVEEVRVF	HLA-B*44:03	36.00		
VP1	327	334	8	SQVEEVRV	HLA-A*02:06	14.00		
VP1	327	335	9	SQVEEVRVF	HLA-B*15:01	18.00		
VP1	331	340	10	EVRVFDGTER	HLA-A*68:01	29.00		
VP1	333	340	8	RVFDGTER	HLA-A*31:01	14.00	HLA-A*68:01	40.00
VP1	333	341	9	RVFDGTERL	HLA-C*12:03	29.00		
VP1	337	350	14	GTERLPGDPDMIRY	HLA-A*01:01	16.00		
VP1	347	360	14	MIRYIDKQGQLQTK	HLA-A*30:01	13.00		
VP1	349	357	9	RYIDKQGQL	HLA-C*14:02	47.00		
VP1	349	361	13	RYIDKQGQLQTKM	HLA-C*14:02	34.00		
VP1	349	362	14	RYIDKQGQLQTKML	HLA-C*14:02	18.00		
VP1	350	357	8	YIDKQGQL	HLA-C*05:01	11.00	HLA-C*08:02	46.00
VP1	350	359	10	YIDKQGQLQT	HLA-C*05:01	35.00		
VP1	350	361	12	YIDKQGQLQTKM	HLA-C*05:01	11.00		
VP1	350	362	13	YIDKQGQLQTKML	HLA-C*05:01	8.00	HLA-C*08:02	26.00

VP2	2	11	10	GAALALLGDL	HLA-C*03:03	47.00						
VP2	3	11	9	AALALLGDL	HLA-C*03:03	40.00						
VP2	3	15	13	AALALLGDLVASV	HLA-A*02:06	25.00						
VP2	4	15	12	ALALLGDLVASV	HLA-A*02:01	44.00						
VP2	5	15	11	LALLGDLVASV	HLA-C*12:03	39.00						
VP2	6	15	10	ALLGDLVASV	HLA-A*02:01	8.00	HLA-A*02:06	11.00				
VP2	6	18	13	ALLGDLVASVSEA	HLA-A*02:01	30.00	HLA-A*02:06	23.00				
VP2	6	19	14	ALLGDLVASVSEAA	HLA-A*02:06	24.00						
VP2	7	15	9	LLGDLVASV	HLA-A*02:01	10.00	HLA-A*02:06	19.00				
VP2	11	19	9	LVASVSEAA	HLA-A*68:02	19.00						
VP2	11	24	14	LVASVSEAAAATGF	HLA-B*15:01	32.00						
VP2	14	26	13	SVSEAAAATGFSV	HLA-A*02:06	7.00	HLA-A*68:02	12.00				
VP2	15	24	10	VSEAAAATGF	HLA-C*05:01	26.00						
VP2	16	24	9	SEAAAATGF	HLA-B*40:01	40.00	HLA-B*44:02	29.00	HLA-B*44:03	19.00	HLA-B*44:03	19.00
VP2	16	26	11	SEAAAATGFSV	HLA-B*40:01	28.00	HLA-B*40:02	44.00				
VP2	16	29	14	SEAAAATGFSVAEI	HLA-B*40:01	38.00						
VP2	17	24	8	EAAAATGF	HLA-B*35:01	18.00						
VP2	17	25	9	EAAAATGFS	HLA-A*68:02	35.00						
VP2	17	26	10	EAAAATGFSV	HLA-A*68:02	3.00						
VP2	17	27	11	EAAAATGFSVA	HLA-A*68:02	16.00						
VP2	17	29	13	EAAAATGFSVAEI	HLA-A*68:02	4.00						
VP2	17	30	14	EAAAATGFSVAEIA	HLA-A*68:02	17.00						
VP2	18	26	9	AAAATGFSV	HLA-A*02:06	25.00						
VP2	18	29	12	AAAATGFSVAEI	HLA-C*03:03	25.00						
VP2	19	26	8	AAATGFSV	HLA-A*02:06	33.00						
VP2	19	29	11	AAATGFSVAEI	HLA-C*03:03	17.00						
VP2	25	34	10	SVAEIAAGEA	HLA-A*68:02	39.00						
VP2	25	35	11	SVAEIAAGEAA	HLA-A*68:02	14.00						
VP2	25	36	12	SVAEIAAGEAAA	HLA-A*02:06	47.00	HLA-A*68:02	23.00				
VP2	25	37	13	SVAEIAAGEAAAA	HLA-A*02:06	37.00	HLA-A*68:02	19.00				
VP2	25	38	14	SVAEIAAGEAAAAI	HLA-A*02:06	15.00	HLA-A*68:02	6.00				

VP2	27	35	9	AEIAAGEAA	HLA-B*40:02	35.00								
VP2	27	38	12	AEIAAGEAAAAI	HLA-B*40:01	5.00	HLA-B*40:02	24.00	HLA-B*44:02	26.00	HLA-B*44:03	22.00	HLA-B*44:03	22.00
VP2	27	40	14	AEIAAGEAAAAIEV	HLA-B*40:01	10.00	HLA-B*40:02	18.00	HLA-B*44:03	36.00	HLA-B*44:03	36.00		
VP2	28	35	8	EIAAGEAA	HLA-A*68:02	37.00								
VP2	28	36	9	EIAAGEAAA	HLA-A*68:02	45.00								
VP2	28	37	10	EIAAGEAAAA	HLA-A*68:02	37.00								
VP2	28	38	11	EIAAGEAAAAI	HLA-A*68:02	11.00								
VP2	28	40	13	EIAAGEAAAAIEV	HLA-A*68:02	7.00								
VP2	29	38	10	IAAGEAAAAI	HLA-C*03:03	3.00	HLA-C*12:03	20.00						
VP2	29	40	12	IAAGEAAAAIEV	HLA-C*03:03	12.00	HLA-C*12:03	38.00						
VP2	29	42	14	IAAGEAAAAIEVQI	HLA-C*03:03	10.00								
VP2	32	40	9	GEAAAAIEV	HLA-B*40:01	9.00								
VP2	32	42	11	GEAAAAIEVQI	HLA-B*40:01	12.00								
VP2	32	45	14	GEAAAAIEVQIASL	HLA-B*40:01	5.00	HLA-B*40:02	34.00						
VP2	33	40	8	EAAAAIEV	HLA-A*68:02	4.00								
VP2	33	42	10	EAAAAIEVQI	HLA-A*68:02	11.00								
VP2	33	45	13	EAAAAIEVQIASL	HLA-A*68:02	7.00	HLA-C*03:03	10.00						
VP2	33	46	14	EAAAAIEVQIASLA	HLA-A*68:02	9.00								
VP2	34	45	12	AAAAIEVQIASL	HLA-C*03:03	6.00								
VP2	35	45	11	AAAIEVQIASL	HLA-C*03:03	5.00								
VP2	36	45	10	AAIEVQIASL	HLA-C*03:03	5.00								
VP2	36	48	13	AAIEVQIASLATV	HLA-A*02:06	11.00	HLA-C*03:03	45.00	HLA-C*12:03	23.00				
VP2	38	45	8	IEVQIASL	HLA-B*40:01	13.00	HLA-C*03:03	32.00						
VP2	39	48	10	EVQIASLATV	HLA-A*68:02	20.00								
VP2	40	47	8	VQIASLAT	HLA-A*02:06	29.00								
VP2	40	48	9	VQIASLATV	HLA-A*02:01	23.00	HLA-A*02:06	2.00						
VP2	40	51	12	VQIASLATVEGI	HLA-A*02:06	6.00								
VP2	41	48	8	QIASLATV	HLA-A*02:06	32.00	HLA-A*68:02	26.00						
VP2	41	51	11	QIASLATVEGI	HLA-A*68:02	49.00								
VP2	45	58	14	LATVEGITSTSEAI	HLA-C*03:03	18.00								
VP2	46	58	13	ATVEGITSTSEAI	HLA-A*02:06	26.00								

VP2	53	61	9	STSEAIAAI	HLA-A*02:06	29.00	HLA-A*68:02	9.00						
VP2	53	63	11	STSEAIAAIGL	HLA-A*68:02	26.00								
VP2	55	63	9	SEAIAAIGL	HLA-B*40:01	6.00								
VP2	55	68	14	SEAIAAIGLTPQTY	HLA-B*18:01	30.00	HLA-B*44:02	23.00	HLA-B*44:03	30.00	HLA-B*44:03	30.00	HLA-C*03:03	15.00
VP2	56	63	8	EAIAAIGL	HLA-A*68:02	14.00								
VP2	56	68	13	EAIAAIGLTPQTY	HLA-A*26:01	48.00	HLA-B*35:01	11.00						
VP2	56	69	14	EAIAAIGLTPQTYA	HLA-A*68:02	13.00								
VP2	57	70	14	AIAAIGLTPQTYAV	HLA-A*02:01	33.00	HLA-A*02:06	12.00	HLA-A*68:02	29.00				
VP2	58	68	11	IAAIGLTPQTY	HLA-B*35:01	12.00	HLA-C*03:03	44.00	HLA-C*12:03	41.00				
VP2	58	70	13	IAAIGLTPQTYAV	HLA-A*02:06	42.00	HLA-C*03:03	15.00						
VP2	58	71	14	IAAIGLTPQTYAVI	HLA-C*03:03	5.00	HLA-C*12:03	29.00						
VP2	59	68	10	AAIGLTPQTY	HLA-B*15:01	42.00								
VP2	59	70	12	AAIGLTPQTYAV	HLA-A*02:06	20.00	HLA-C*03:03	39.00						
VP2	59	71	13	AAIGLTPQTYAVI	HLA-C*03:03	9.00								
VP2	62	70	9	GLTPQTYAV	HLA-A*02:01	15.00	HLA-A*02:06	25.00						
VP2	63	70	8	LTPQTYAV	HLA-A*68:02	36.00								
VP2	66	74	9	QTYAVIAGA	HLA-A*68:02	7.00								
VP2	66	78	13	QTYAVIAGAPGAI	HLA-A*68:02	12.00	HLA-C*12:03	50.00	HLA-C*14:02	20.00				
VP2	67	78	12	TYAVIAGAPGAI	HLA-C*14:02	10.00								
VP2	68	76	9	YAVIAGAPG	HLA-C*03:03	17.00								
VP2	68	77	10	YAVIAGAPGA	HLA-C*03:03	18.00								
VP2	68	78	11	YAVIAGAPGAI	HLA-C*03:03	2.00	HLA-C*12:03	11.00						
VP2	68	79	12	YAVIAGAPGAIA	HLA-C*03:03	8.00								
VP2	68	80	13	YAVIAGAPGAIAG	HLA-C*03:03	46.00								
VP2	68	81	14	YAVIAGAPGAIAGF	HLA-B*35:01	18.00	HLA-C*03:03	2.00						
VP2	69	77	9	AVIAGAPGA	HLA-A*02:06	25.00								
VP2	69	81	13	AVIAGAPGAIAGF	HLA-B*15:01	45.00								
VP2	71	78	8	IAGAPGAI	HLA-C*03:03	37.00								
VP2	71	84	14	IAGAPGAIAGFAAL	HLA-C*03:03	3.00								
VP2	72	84	13	AGAPGAIAGFAAL	HLA-C*03:03	22.00								
VP2	73	84	12	GAPGAIAGFAAL	HLA-C*03:03	14.00								

VP2	74	84	11	APGAIAGFAAL	HLA-B*07:02	19.00						
VP2	76	84	9	GAIAGFAAL	HLA-A*02:06	26.00	HLA-C*03:03	2.00				
VP2	76	85	10	GAIAGFAALI	HLA-C*03:03	25.00						
VP2	76	88	13	GAIAGFAALIQTV	HLA-A*02:06	18.00	HLA-C*03:03	33.00	HLA-C*12:03	43.00		
VP2	77	84	8	AIAGFAAL	HLA-A*02:06	39.00						
VP2	77	88	12	AIAGFAALIQTV	HLA-A*02:01	47.00	HLA-A*02:06	18.00				
VP2	78	88	11	IAGFAALIQTV	HLA-C*12:03	35.00						
VP2	81	88	8	FAALIQTV	HLA-A*02:06	28.00	HLA-A*68:02	17.00	HLA-C*03:03	6.00	HLA-C*12:03	6.00
VP2	81	91	11	FAALIQTVSGI	HLA-A*68:02	21.00	HLA-C*03:03	5.00	HLA-C*12:03	41.00		
VP2	81	94	14	FAALIQTVSGISSL	HLA-A*68:02	39.00	HLA-C*03:03	2.00	HLA-C*12:03	20.00		
VP2	82	94	13	AALIQTVSGISSL	HLA-C*03:03	6.00						
VP2	83	94	12	ALIQTVSGISSL	HLA-A*02:01	39.00						
VP2	86	97	12	QTVSGISSLAQV	HLA-A*68:02	18.00						
VP2	86	99	14	QTVSGISSLAQVGY	HLA-A*26:01	39.00						
VP2	87	94	8	TVSGISSL	HLA-A*68:02	16.00						
VP2	87	95	9	TVSGISSLA	HLA-A*68:02	8.00						
VP2	87	97	11	TVSGISSLAQV	HLA-A*68:02	6.00						
VP2	87	99	13	TVSGISSLAQVGY	HLA-A*29:02	25.00						
VP2	87	100	14	TVSGISSLAQVGYK	HLA-A*11:01	17.00	HLA-A*68:01	11.00				
VP2	88	100	13	VSGISSLAQVGYK	HLA-A*11:01	38.00						
VP2	91	100	10	ISSLAQVGYK	HLA-A*11:01	40.00						
VP2	91	101	11	ISSLAQVGYKF	HLA-B*58:01	40.00						
VP2	92	100	9	SSLAQVGYK	HLA-A*03:01	42.00	HLA-A*11:01	14.00	HLA-A*30:01	22.00		
VP2	92	105	14	SSLAQVGYKFFDDW	HLA-B*58:01	20.00						
VP2	93	100	8	SLAQVGYK	HLA-A*03:01	37.00	HLA-A*11:01	29.00				
VP2	94	102	9	LAQVGYKFF	HLA-C*03:03	47.00						
VP2	99	112	14	YKFFDDWDHKVSTV	HLA-C*12:03	42.00						
VP2	100	112	13	KFFDDWDHKVSTV	HLA-C*12:03	33.00						
VP2	101	109	9	FFDDWDHKV	HLA-C*12:03	47.00						
VP2	101	112	12	FFDDWDHKVSTV	HLA-C*12:03	14.00						
VP2	107	114	8	HKVSTVGL	HLA-B*39:01	31.00						

VP2	108	115	8	KVSTVGLY	HLA-A*29:02	38.00	HLA-A*30:02	17.00	HLA-A*80:01	19.00		
VP2	110	120	11	STVGLYQQSGM	HLA-A*26:01	38.00						
VP2	110	122	13	STVGLYQQSGMAL	HLA-C*03:03	20.00						
VP2	113	122	10	GLYQQSGMAL	HLA-C*03:03	45.00						
VP2	114	122	9	LYQQSGMAL	HLA-C*14:02	4.00						
VP2	114	124	11	LYQQSGMALEL	HLA-C*14:02	5.00						
VP2	114	125	12	LYQQSGMALELF	HLA-A*23:01	18.00	HLA-A*24:02	30.00	HLA-C*14:02	16.00		
VP2	115	122	8	YQQSGMAL	HLA-A*02:06	25.00	HLA-B*15:01	37.00	HLA-B*39:01	8.00	HLA-C*03:03	40.00
VP2	115	124	10	YQQSGMALEL	HLA-B*15:01	25.00	HLA-B*39:01	13.00				
VP2	115	125	11	YQQSGMALELF	HLA-B*15:01	22.00						
VP2	116	124	9	QQSGMALEL	HLA-B*39:01	22.00						
VP2	116	125	10	QQSGMALELF	HLA-B*15:01	33.00						
VP2	119	130	12	GMALELFNPDEY	HLA-A*29:02	14.00						
VP2	119	131	13	GMALELFNPDEYY	HLA-A*29:02	10.00						
VP2	120	130	11	MALELFNPDEY	HLA-B*35:01	2.00	HLA-C*12:03	16.00				
VP2	120	131	12	MALELFNPDEYY	HLA-A*29:02	28.00	HLA-B*35:01	5.00	HLA-B*53:01	27.00	HLA-C*12:03	12.00
VP2	120	133	14	MALELFNPDEYYDI	HLA-C*03:03	29.00	HLA-C*12:03	31.00				
VP2	122	133	12	LELFNPDEYYDI	HLA-B*40:02	48.00						
VP2	122	134	13	LELFNPDEYYDIL	HLA-B*40:01	22.00	HLA-B*40:02	24.00				
VP2	124	131	8	LFNPDEYY	HLA-A*29:02	25.00						
VP2	128	141	14	DEYYDILFPGVNTF	HLA-B*18:01	3.00						
VP2	129	138	10	EYYDILFPGV	HLA-C*14:02	16.00						
VP2	129	141	13	EYYDILFPGVNTF	HLA-A*23:01	20.00	HLA-C*14:02	6.00				
VP2	129	142	14	EYYDILFPGVNTFV	HLA-A*68:02	6.00	HLA-C*14:02	9.00				
VP2	130	138	9	YYDILFPGV	HLA-A*02:06	22.00	HLA-C*14:02	30.00				
VP2	130	141	12	YYDILFPGVNTF	HLA-A*24:02	33.00	HLA-C*14:02	7.00				
VP2	130	142	13	YYDILFPGVNTFV	HLA-A*02:06	38.00	HLA-C*14:02	14.00				
VP2	132	142	11	DILFPGVNTFV	HLA-A*68:02	40.00						
VP2	133	141	9	ILFPGVNTF	HLA-A*32:01	21.00	HLA-B*15:01	28.00	HLA-C*12:03	15.00		
VP2	133	142	10	ILFPGVNTFV	HLA-A*02:01	5.00	HLA-A*02:06	26.00	HLA-C*12:03	31.00		
VP2	133	145	13	ILFPGVNTFVNNI	HLA-A*02:01	12.00	HLA-C*12:03	14.00				

VP2	134	141	8	LFPGVNTF	HLA-C*14:02	31.00						
VP2	134	145	12	LFPGVNTFVNNI	HLA-C*14:02	40.00						
VP2	135	145	11	FPGVNTFVNNI	HLA-B*53:01	19.00						
VP2	135	147	13	FPGVNTFVNNIQY	HLA-B*35:01	4.00	HLA-B*53:01	15.00				
VP2	135	148	14	FPGVNTFVNNIQYL	HLA-B*53:01	41.00						
VP2	139	147	9	NTFVNNIQY	HLA-A*68:01	44.00						
VP2	139	148	10	NTFVNNIQYL	HLA-A*68:02	26.00						
VP2	139	151	13	NTFVNNIQYLDPR	HLA-A*33:01	34.00	HLA-A*68:01	6.00				
VP2	140	147	8	TFVNNIQY	HLA-A*29:02	35.00						
VP2	140	148	9	TFVNNIQYL	HLA-C*14:02	8.00						
VP2	141	148	8	FVNNIQYL	HLA-A*02:06	29.00	HLA-A*68:02	24.00	HLA-C*03:03	39.00	HLA-C*12:03	44.00
VP2	141	151	11	FVNNIQYLDPR	HLA-A*31:01	33.00	HLA-A*68:01	12.00				
VP2	145	157	13	IQYLDPRHWGPSL	HLA-C*03:03	28.00						
VP2	146	157	12	QYLDPRHWGPSL	HLA-C*14:02	11.00						
VP2	146	158	13	QYLDPRHWGPSLF	HLA-A*23:01	30.00	HLA-C*14:02	35.00				
VP2	147	157	11	YLDPRHWGPSL	HLA-C*05:01	14.00						
VP2	147	158	12	YLDPRHWGPSLF	HLA-C*05:01	2.00						
VP2	147	159	13	YLDPRHWGPSLFA	HLA-A*02:01	14.00	HLA-A*02:06	13.00	HLA-C*05:01	31.00		
VP2	147	160	14	YLDPRHWGPSLFAT	HLA-A*02:01	25.00	HLA-A*02:06	13.00				
VP2	149	157	9	DPRHWGPSL	HLA-B*07:02	28.00						
VP2	154	165	12	GPSLFATISQAL	HLA-B*07:02	34.00						
VP2	155	166	12	PSLFATISQALW	HLA-B*58:01	46.00						
VP2	156	165	10	SLFATISQAL	HLA-A*02:01	35.00	HLA-C*14:02	22.00				
VP2	156	166	11	SLFATISQALW	HLA-B*58:01	41.00						
VP2	156	168	13	SLFATISQALWHV	HLA-A*02:01	5.00	HLA-A*02:06	5.00				
VP2	156	169	14	SLFATISQALWHVI	HLA-A*02:01	35.00						
VP2	157	165	9	LFATISQAL	HLA-C*03:03	44.00	HLA-C*14:02	30.00				
VP2	158	165	8	FATISQAL	HLA-C*03:03	2.00						
VP2	158	166	9	FATISQALW	HLA-B*53:01	10.00	HLA-B*58:01	7.00				
VP2	158	168	11	FATISQALWHV	HLA-A*68:02	39.00	HLA-C*03:03	31.00	HLA-C*12:03	40.00		
VP2	158	169	12	FATISQALWHVI	HLA-C*03:03	7.00	HLA-C*12:03	17.00				

VP2	158	170	13	FATISQALWHVIR	HLA-A*68:01	16.00				
VP2	159	166	8	ATISQALW	HLA-B*58:01	7.00				
VP2	159	168	10	ATISQALWHV	HLA-A*02:06	13.00				
VP2	159	170	12	ATISQALWHVIR	HLA-A*31:01	47.00				
VP2	160	168	9	TISQALWHV	HLA-A*02:01	40.00	HLA-A*02:06	7.00	HLA-A*68:02	23.00
VP2	160	170	11	TISQALWHVIR	HLA-A*68:01	35.00				
VP2	161	170	10	ISQALWHVIR	HLA-A*31:01	22.00				
VP2	162	169	8	SQALWHVI	HLA-A*02:06	37.00	HLA-B*39:01	33.00		
VP2	162	170	9	SQALWHVIR	HLA-A*31:01	23.00				
VP2	163	176	14	QALWHVIRDDIPSI	HLA-C*12:03	28.00				
VP2	164	176	13	ALWHVIRDDIPSI	HLA-A*02:01	17.00				
VP2	165	176	12	LWHVIRDDIPSI	HLA-C*14:02	48.00				
VP2	166	176	11	WHVIRDDIPSI	HLA-B*38:01	48.00				
VP2	169	181	13	IRDDIPSITSQEL	HLA-C*06:02	45.00	HLA-C*07:01	24.00		
VP2	173	181	9	IPSITSQEL	HLA-B*07:02	33.00				
VP2	177	190	14	TSQELQRRTERFFR	HLA-A*31:01	32.00	HLA-A*33:01	49.00	HLA-A*68:01	25.00
VP2	178	190	13	SQELQRRTERFFR	HLA-A*31:01	50.00				
VP2	180	187	8	ELQRRTER	HLA-A*33:01	39.00				
VP2	180	190	11	ELQRRTERFFR	HLA-A*33:01	9.00	HLA-A*68:01	43.00		
VP2	181	188	8	LQRRTERF	HLA-B*15:01	28.00				
VP2	181	189	9	LQRRTERFF	HLA-B*15:01	32.00				
VP2	181	190	10	LQRRTERFFR	HLA-A*31:01	19.00				
VP2	183	190	8	RRTERFFR	HLA-B*27:05	47.00				
VP2	184	195	12	RTERFFRDSLAR	HLA-A*31:01	33.00				
VP2	185	193	9	TERFFRDSL	HLA-B*40:02	34.00				
VP2	187	195	9	RFFRDSLAR	HLA-A*31:01	19.00				
VP2	187	196	10	RFFRDSLARF	HLA-A*23:01	32.00				
VP2	187	197	11	RFFRDSLARFL	HLA-C*14:02	44.00				
VP2	188	195	8	FFRDSLAR	HLA-A*33:01	24.00				
VP2	188	196	9	FFRDSLARF	HLA-C*14:02	35.00				
VP2	188	197	10	FFRDSLARFL	HLA-C*14:02	14.00				

VP2	189	197	9	FRDSLARFL	HLA-B*39:01	26.00	HLA-C*06:02	16.00	HLA-C*07:01	29.00
VP2	191	202	12	DSLARFLEETTW	HLA-B*58:01	38.00				
VP2	192	204	13	SLARFLEETTWTI	HLA-A*02:01	23.00	HLA-A*02:06	46.00	HLA-A*32:01	32.00
VP2	192	205	14	SLARFLEETTWTIV	HLA-A*02:01	30.00	HLA-A*02:06	33.00		
VP2	196	204	9	FLEETTWTI	HLA-A*02:01	8.00	HLA-A*02:06	6.00		
VP2	196	205	10	FLEETTWTIV	HLA-A*02:01	21.00	HLA-A*02:06	25.00		
VP2	196	207	12	FLEETTWTIVNA	HLA-A*02:01	20.00	HLA-A*02:06	32.00		
VP2	196	209	14	FLEETTWTIVNAPI	HLA-A*02:01	13.00	HLA-A*02:06	14.00		
VP2	197	204	8	LEETTWTI	HLA-B*40:01	42.00				
VP2	197	209	13	LEETTWTIVNAPI	HLA-B*40:01	20.00				
VP2	199	207	9	ETTWTIVNA	HLA-A*68:02	15.00				
VP2	199	209	11	ETTWTIVNAPI	HLA-A*68:02	4.00				
VP2	199	212	14	ETTWTIVNAPINFY	HLA-A*26:01	18.00	HLA-A*68:01	19.00		
VP2	200	207	8	TTWTIVNA	HLA-A*68:02	21.00				
VP2	200	209	10	TTWTIVNAPI	HLA-A*68:02	4.00	HLA-C*14:02	30.00		
VP2	200	211	12	TTWTIVNAPINF	HLA-A*32:01	47.00				
VP2	200	212	13	TTWTIVNAPINFY	HLA-A*11:01	48.00	HLA-A*29:02	12.00	HLA-A*30:02	36.00
VP2	201	209	9	TWTIVNAPI	HLA-C*14:02	37.00				
VP2	201	214	14	TWTIVNAPINFYNY	HLA-A*29:02	41.00				
VP2	202	209	8	WTIVNAPI	HLA-A*68:02	16.00				
VP2	202	214	13	WTIVNAPINFYNY	HLA-A*29:02	7.00	HLA-A*30:02	47.00		
VP2	202	215	14	WTIVNAPINFYNYI	HLA-A*68:02	28.00				
VP2	203	214	12	TIVNAPINFYNY	HLA-A*29:02	27.00				
VP2	203	215	13	TIVNAPINFYNYI	HLA-A*68:02	38.00				
VP2	204	212	9	IVNAPINFY	HLA-A*30:02	25.00				
VP2	204	215	12	IVNAPINFYNYI	HLA-A*68:02	29.00	HLA-C*12:03	30.00		
VP2	207	214	8	APINFYNY	HLA-B*35:01	36.00				
VP2	210	218	9	NFYNYIQQY	HLA-A*29:02	28.00	HLA-C*14:02	36.00		
VP2	210	219	10	NFYNYIQQYY	HLA-A*29:02	12.00	HLA-C*14:02	24.00		
VP2	210	222	13	NFYNYIQQYYSDL	HLA-C*14:02	11.00				
VP2	211	218	8	FYNYIQQY	HLA-A*29:02	30.00	HLA-C*14:02	33.00		

VP2	211	219	9	FYNYIQQYY	HLA-A*29:02	14.00	HLA-C*14:02	27.00				
VP2	211	222	12	FYNYIQQYYSDL	HLA-C*14:02	6.00						
VP2	212	219	8	YNYIQQYY	HLA-A*29:02	12.00						
VP2	212	222	11	YNYIQQYYSDL	HLA-C*03:03	17.00						
VP2	213	222	10	NYIQQYYSDL	HLA-C*14:02	16.00						
VP2	213	225	13	NYIQQYYSDLSPI	HLA-C*14:02	8.00						
VP2	216	225	10	QQYYSDLSPI	HLA-A*02:06	30.00						
VP2	216	229	14	QQYYSDLSPIRPSM	HLA-B*15:01	41.00	HLA-C*14:02	20.00				
VP2	217	225	9	QYYSDLSPI	HLA-A*23:01	38.00	HLA-C*14:02	3.00				
VP2	217	229	13	QYYSDLSPIRPSM	HLA-C*14:02	3.00						
VP2	217	230	14	QYYSDLSPIRPSMV	HLA-C*14:02	4.00						
VP2	218	225	8	YYSDLSPI	HLA-C*12:03	33.00	HLA-C*14:02	3.00				
VP2	218	229	12	YYSDLSPIRPSM	HLA-C*07:02	48.00	HLA-C*14:02	4.00				
VP2	218	230	13	YYSDLSPIRPSMV	HLA-C*12:03	32.00	HLA-C*14:02	7.00				
VP2	219	229	11	YSDLSPIRPSM	HLA-C*05:01	16.00						
VP2	219	230	12	YSDLSPIRPSMV	HLA-A*01:01	30.00	HLA-C*05:01	2.00	HLA-C*12:03	27.00		
VP2	223	233	11	SPIRPSMVRQV	HLA-B*07:02	33.00						
VP2	223	234	12	SPIRPSMVRQVA	HLA-B*07:02	28.00						
VP2	225	233	9	IRPSMVRQV	HLA-C*06:02	29.00	HLA-C*07:01	26.00				
VP2	226	234	9	RPSMVRQVA	HLA-B*07:02	15.00						
VP2	228	236	9	SMVRQVAER	HLA-A*31:01	35.00						
VP2	229	236	8	MVRQVAER	HLA-A*31:01	31.00	HLA-A*33:01	31.00	HLA-A*68:01	20.00		
VP2	229	240	12	MVRQVAEREGTR	HLA-A*33:01	39.00	HLA-A*68:01	45.00				
VP2	231	243	13	RQVAEREGTRVHF	HLA-B*15:01	18.00						
VP2	233	243	11	VAEREGTRVHF	HLA-C*05:01	37.00						
VP2	234	247	14	AEREGTRVHFGHTY	HLA-B*18:01	18.00	HLA-B*44:03	42.00	HLA-B*44:03	42.00		
VP2	236	249	14	REGTRVHFGHTYSI	HLA-B*40:02	38.00						
VP2	238	248	11	GTRVHFGHTYS	HLA-A*30:01	28.00						
VP2	238	249	12	GTRVHFGHTYSI	HLA-A*30:01	10.00						
VP2	239	249	11	TRVHFGHTYSI	HLA-B*39:01	28.00						
VP2	240	247	8	RVHFGHTY	HLA-A*29:02	33.00	HLA-A*30:01	20.00	HLA-A*30:02	38.00	HLA-A*80:01	28.00

VP2	240	248	9	RVHFGHTYS	HLA-A*30:01	24.00								
VP2	241	249	9	VHFGHTYSI	HLA-B*39:01	15.00	HLA-C*06:02	14.00	HLA-C*07:01	8.00	HLA-C*14:02	42.00		
VP2	245	255	11	HTYSIDDADSI	HLA-A*68:02	12.00	HLA-C*14:02	22.00	HLA-C*15:02	42.00				
VP2	245	258	14	HTYSIDDADSIEEV	HLA-A*68:02	3.00	HLA-C*12:03	45.00	HLA-C*15:02	28.00				
VP2	246	255	10	TYSIDDADSI	HLA-C*14:02	18.00								
VP2	246	258	13	TYSIDDADSIEEV	HLA-C*14:02	34.00								
VP2	247	255	9	YSIDDADSI	HLA-C*03:03	7.00	HLA-C*12:03	7.00	HLA-C*15:02	32.00				
VP2	247	258	12	YSIDDADSIEEV	HLA-A*02:06	13.00	HLA-A*68:02	20.00	HLA-C*03:03	17.00	HLA-C*12:03	6.00	HLA-C*15:02	22.00
VP2	248	258	11	SIDDADSIEEV	HLA-C*05:01	33.00								
VP2	251	261	11	DADSIEEVTQR	HLA-A*68:01	50.00								
VP2	253	261	9	DSIEEVTQR	HLA-A*68:01	17.00								
VP2	253	265	13	DSIEEVTQRMDLR	HLA-A*68:01	19.00								
VP2	257	265	9	EVTQRMDLR	HLA-A*33:01	37.00	HLA-A*68:01	12.00						
VP2	257	270	14	EVTQRMDLRNQQSV	HLA-A*68:02	33.00								
VP2	260	270	11	QRMDLRNQQSV	HLA-C*06:02	23.00	HLA-C*07:01	17.00						
VP2	266	275	10	NQQSVHSGEF	HLA-B*15:01	21.00								
VP2	267	275	9	QQSVHSGEF	HLA-B*15:01	24.00								
VP2	269	278	10	SVHSGEFIEK	HLA-A*11:01	22.00								
VP2	278	288	11	KTIAPGGANQR	HLA-A*31:01	37.00								
VP2	279	288	10	TIAPGGANQR	HLA-A*68:01	31.00								
VP2	280	293	14	IAPGGANQRTAPQW	HLA-B*58:01	48.00								
VP2	287	297	11	QRTAPQWMLPL	HLA-B*39:01	34.00								
VP2	288	297	10	RTAPQWMLPL	HLA-C*15:02	41.00								
VP2	288	298	11	RTAPQWMLPLL	HLA-A*30:01	44.00	HLA-B*58:01	44.00						
VP2	288	299	12	RTAPQWMLPLLL	HLA-B*58:01	30.00								
VP2	288	301	14	RTAPQWMLPLLLGL	HLA-A*02:06	40.00								
VP2	290	298	9	APQWMLPLL	HLA-B*07:02	29.00								
VP2	290	301	12	APQWMLPLLLGL	HLA-B*07:02	21.00								
VP2	293	301	9	WMLPLLLGL	HLA-A*02:01	9.00	HLA-A*02:06	6.00						
VP2	293	302	10	WMLPLLLGLY	HLA-A*29:02	37.00	HLA-A*80:01	45.00	HLA-B*15:01	28.00				
VP2	293	304	12	WMLPLLLGLYGT	HLA-A*02:01	29.00	HLA-A*02:06	19.00						

VP2	293	305	13	WMLPLLLGLYGTV	HLA-A*02:01	14.00	HLA-A*02:06	5.00		
VP2	294	302	9	MLPLLLGLY	HLA-A*29:02	20.00				
VP2	295	302	8	LPLLLGLY	HLA-B*35:01	14.00				
VP2	297	305	9	LLLGLYGTV	HLA-A*02:01	46.00	HLA-A*02:06	27.00		
VP2	297	308	12	LLLGLYGTVTPA	HLA-A*02:01	37.00	HLA-A*02:06	50.00		
VP2	297	309	13	LLLGLYGTVTPAL	HLA-A*02:01	19.00	HLA-A*02:06	34.00	HLA-C*03:03	24.00
VP2	300	308	9	GLYGTVTPA	HLA-A*02:01	24.00				
VP2	300	309	10	GLYGTVTPAL	HLA-A*02:01	20.00	HLA-C*03:03	24.00	HLA-C*14:02	24.00
VP2	300	312	13	GLYGTVTPALEAY	HLA-A*29:02	24.00	HLA-B*15:01	41.00		
VP2	301	309	9	LYGTVTPAL	HLA-C*14:02	8.00				
VP2	304	312	9	TVTPALEAY	HLA-B*35:01	17.00				
VP2	310	321	12	EAYEDGPNQKKR	HLA-A*68:01	28.00				
VP2	310	322	13	EAYEDGPNQKKRR	HLA-A*68:01	40.00				
VP2	310	323	14	EAYEDGPNQKKRRV	HLA-C*12:03	11.00				
VP2	317	325	9	NQKKRRVSR	HLA-A*31:01	17.00				
VP2	319	330	12	KKRRVSRGSSQK	HLA-A*30:01	12.00				
VP2	319	332	14	KKRRVSRGSSQKAK	HLA-A*30:01	19.00				
VP2	320	330	11	KRRVSRGSSQK	HLA-A*30:01	18.00				
VP2	320	332	13	KRRVSRGSSQKAK	HLA-A*30:01	27.00				
VP2	321	330	10	RRVSRGSSQK	HLA-B*27:05	36.00				
VP2	321	332	12	RRVSRGSSQKAK	HLA-B*27:05	42.00				
VP2	322	330	9	RVSRGSSQK	HLA-A*03:01	47.00	HLA-A*11:01	23.00	HLA-A*30:01	25.00
VP2	322	332	11	RVSRGSSQKAK	HLA-A*30:01	36.00				
VP2	322	335	14	RVSRGSSQKAKGTR	HLA-A*31:01	42.00				
VP2	323	330	8	VSRGSSQK	HLA-A*30:01	14.00				
VP2	328	335	8	SQKAKGTR	HLA-A*31:01	36.00				
VP2	330	338	9	KAKGTRASA	HLA-A*30:01	6.00				
VP2	330	343	14	KAKGTRASAKTTNK	HLA-A*30:01	5.00				
VP2	333	343	11	GTRASAKTTNK	HLA-A*30:01	6.00				
VP2	335	345	11	RASAKTTNKRR	HLA-A*31:01	44.00				

VP2	335	347	13	RASAKTTNKRRSR	HLA-A*31:01	25.00							
VP2	336	343	8	ASAKTTNK	HLA-A*11:01	30.00							
VP2	337	350	14	SAKTTNKRRSRSSR	HLA-A*31:01	42.00							
VP2	339	347	9	KTTNKRRSR	HLA-A*31:01	17.00							
VP2	339	350	12	KTTNKRRSRSSR	HLA-A*31:01	14.00							
VP2	340	347	8	TTNKRRSR	HLA-A*31:01	23.00	HLA-A*33:01	25.00	HLA-A*68:01	35.00			
VP2	340	350	11	TTNKRRSRSSR	HLA-A*31:01	15.00	HLA-A*33:01	18.00	HLA-A*68:01	14.00			
VP3	1	11	11	MALELFNPDEY	HLA-B*35:01	2.00	HLA-C*12:03	16.00					
VP3	1	12	12	MALELFNPDEYY	HLA-A*29:02	28.00	HLA-B*35:01	5.00	HLA-B*53:01	27.00	HLA-C*12:03	12.00	
VP3	1	14	14	MALELFNPDEYYDI	HLA-C*03:03	29.00	HLA-C*12:03	31.00					
VP3	3	14	12	LELFNPDEYYDI	HLA-B*40:02	48.00							
VP3	3	15	13	LELFNPDEYYDIL	HLA-B*40:01	22.00	HLA-B*40:02	24.00					
VP3	5	12	8	LFNPDEYY	HLA-A*29:02	25.00							
VP3	9	22	14	DEYYDILFPGVNTF	HLA-B*18:01	3.00							
VP3	10	19	10	EYYDILFPGV	HLA-C*14:02	16.00							
VP3	10	22	13	EYYDILFPGVNTF	HLA-A*23:01	20.00	HLA-C*14:02	6.00					
VP3	10	23	14	EYYDILFPGVNTFV	HLA-A*68:02	6.00	HLA-C*14:02	9.00					
VP3	11	19	9	YYDILFPGV	HLA-A*02:06	22.00	HLA-C*14:02	30.00					
VP3	11	22	12	YYDILFPGVNTF	HLA-A*24:02	33.00	HLA-C*14:02	7.00					
VP3	11	23	13	YYDILFPGVNTFV	HLA-A*02:06	38.00	HLA-C*14:02	14.00					
VP3	13	23	11	DILFPGVNTFV	HLA-A*68:02	40.00							
VP3	14	22	9	ILFPGVNTF	HLA-A*32:01	21.00	HLA-B*15:01	28.00	HLA-C*12:03	15.00			
VP3	14	23	10	ILFPGVNTFV	HLA-A*02:01	5.00	HLA-A*02:06	26.00	HLA-C*12:03	31.00			
VP3	14	26	13	ILFPGVNTFVNNI	HLA-A*02:01	12.00	HLA-C*12:03	14.00					
VP3	15	22	8	LFPGVNTF	HLA-C*14:02	31.00							
VP3	15	26	12	LFPGVNTFVNNI	HLA-C*14:02	40.00							
VP3	16	26	11	FPGVNTFVNNI	HLA-B*53:01	19.00							
VP3	16	28	13	FPGVNTFVNNIQY	HLA-B*35:01	4.00	HLA-B*53:01	15.00					
VP3	16	29	14	FPGVNTFVNNIQYL	HLA-B*53:01	41.00							
VP3	20	28	9	NTFVNNIQY	HLA-A*68:01	44.00							
VP3	20	29	10	NTFVNNIQYL	HLA-A*68:02	26.00							

VP3	20	32	13	NTFVNNIQYLDPR	HLA-A*33:01	34.00	HLA-A*68:01	6.00				
VP3	21	28	8	TFVNNIQY	HLA-A*29:02	35.00						
VP3	21	29	9	TFVNNIQYL	HLA-C*14:02	8.00						
VP3	22	29	8	FVNNIQYL	HLA-A*02:06	29.00	HLA-A*68:02	24.00	HLA-C*03:03	39.00	HLA-C*12:03	44.00
VP3	22	32	11	FVNNIQYLDPR	HLA-A*31:01	33.00	HLA-A*68:01	12.00				
VP3	26	38	13	IQYLDPRHWGPSL	HLA-C*03:03	28.00						
VP3	27	38	12	QYLDPRHWGPSL	HLA-C*14:02	11.00						
VP3	27	39	13	QYLDPRHWGPSLF	HLA-A*23:01	30.00	HLA-C*14:02	35.00				
VP3	28	38	11	YLDPRHWGPSL	HLA-C*05:01	14.00						
VP3	28	39	12	YLDPRHWGPSLF	HLA-C*05:01	2.00						
VP3	28	40	13	YLDPRHWGPSLFA	HLA-A*02:01	14.00	HLA-A*02:06	13.00	HLA-C*05:01	31.00		
VP3	28	41	14	YLDPRHWGPSLFAT	HLA-A*02:01	25.00	HLA-A*02:06	13.00				
VP3	30	38	9	DPRHWGPSL	HLA-B*07:02	28.00						
VP3	35	46	12	GPSLFATISQAL	HLA-B*07:02	34.00						
VP3	36	47	12	PSLFATISQALW	HLA-B*58:01	46.00						
VP3	37	46	10	SLFATISQAL	HLA-A*02:01	35.00	HLA-C*14:02	22.00				
VP3	37	47	11	SLFATISQALW	HLA-B*58:01	41.00						
VP3	37	49	13	SLFATISQALWHV	HLA-A*02:01	5.00	HLA-A*02:06	5.00				
VP3	37	50	14	SLFATISQALWHVI	HLA-A*02:01	35.00						
VP3	38	46	9	LFATISQAL	HLA-C*03:03	44.00	HLA-C*14:02	30.00				
VP3	39	46	8	FATISQAL	HLA-C*03:03	2.00						
VP3	39	47	9	FATISQALW	HLA-B*53:01	10.00	HLA-B*58:01	7.00				
VP3	39	49	11	FATISQALWHV	HLA-A*68:02	39.00	HLA-C*03:03	31.00	HLA-C*12:03	40.00		
VP3	39	50	12	FATISQALWHVI	HLA-C*03:03	7.00	HLA-C*12:03	17.00				
VP3	39	51	13	FATISQALWHVIR	HLA-A*68:01	16.00						
VP3	40	47	8	ATISQALW	HLA-B*58:01	7.00						
VP3	40	49	10	ATISQALWHV	HLA-A*02:06	13.00						
VP3	40	51	12	ATISQALWHVIR	HLA-A*31:01	47.00						
VP3	41	49	9	TISQALWHV	HLA-A*02:01	40.00	HLA-A*02:06	7.00	HLA-A*68:02	23.00		
VP3	41	51	11	TISQALWHVIR	HLA-A*68:01	35.00						
VP3	42	51	10	ISQALWHVIR	HLA-A*31:01	22.00						

VP3	43	50	8	SQALWHVI	HLA-A*02:06	37.00	HLA-B*39:01	33.00		
VP3	43	51	9	SQALWHVIR	HLA-A*31:01	23.00				
VP3	44	57	14	QALWHVIRDDIPSI	HLA-C*12:03	28.00				
VP3	45	57	13	ALWHVIRDDIPSI	HLA-A*02:01	17.00				
VP3	46	57	12	LWHVIRDDIPSI	HLA-C*14:02	48.00				
VP3	47	57	11	WHVIRDDIPSI	HLA-B*38:01	48.00				
VP3	50	62	13	IRDDIPSITSQEL	HLA-C*06:02	45.00	HLA-C*07:01	24.00		
VP3	54	62	9	IPSITSQEL	HLA-B*07:02	33.00				
VP3	58	71	14	TSQELQRRTERFFR	HLA-A*31:01	32.00	HLA-A*33:01	49.00	HLA-A*68:01	25.00
VP3	59	71	13	SQELQRRTERFFR	HLA-A*31:01	50.00				
VP3	61	68	8	ELQRRTER	HLA-A*33:01	39.00				
VP3	61	71	11	ELQRRTERFFR	HLA-A*33:01	9.00	HLA-A*68:01	43.00		
VP3	62	69	8	LQRRTERF	HLA-B*15:01	28.00				
VP3	62	70	9	LQRRTERFF	HLA-B*15:01	32.00				
VP3	62	71	10	LQRRTERFFR	HLA-A*31:01	19.00				
VP3	64	71	8	RRTERFFR	HLA-B*27:05	47.00				
VP3	65	76	12	RTERFFRDSLAR	HLA-A*31:01	33.00				
VP3	66	74	9	TERFFRDSL	HLA-B*40:02	34.00				
VP3	68	76	9	RFFRDSLAR	HLA-A*31:01	19.00				
VP3	68	77	10	RFFRDSLARF	HLA-A*23:01	32.00				
VP3	68	78	11	RFFRDSLARFL	HLA-C*14:02	44.00				
VP3	69	76	8	FFRDSLAR	HLA-A*33:01	24.00				
VP3	69	77	9	FFRDSLARF	HLA-C*14:02	35.00				
VP3	69	78	10	FFRDSLARFL	HLA-C*14:02	14.00				
VP3	70	78	9	FRDSLARFL	HLA-B*39:01	26.00	HLA-C*06:02	16.00	HLA-C*07:01	29.00
VP3	72	83	12	DSLARFLEETTW	HLA-B*58:01	38.00				
VP3	73	85	13	SLARFLEETTWTI	HLA-A*02:01	23.00	HLA-A*02:06	46.00	HLA-A*32:01	32.00
VP3	73	86	14	SLARFLEETTWTIV	HLA-A*02:01	30.00	HLA-A*02:06	33.00		
VP3	77	85	9	FLEETTWTI	HLA-A*02:01	8.00	HLA-A*02:06	6.00		
VP3	77	86	10	FLEETTWTIV	HLA-A*02:01	21.00	HLA-A*02:06	25.00		
VP3	77	88	12	FLEETTWTIVNA	HLA-A*02:01	20.00	HLA-A*02:06	32.00		

VP3	77	90	14	FLEETTWTIVNAPI	HLA-A*02:01	13.00	HLA-A*02:06	14.00		
VP3	78	85	8	LEETTWTI	HLA-B*40:01	42.00				
VP3	78	90	13	LEETTWTIVNAPI	HLA-B*40:01	20.00				
VP3	80	88	9	ETTWTIVNA	HLA-A*68:02	15.00				
VP3	80	90	11	ETTWTIVNAPI	HLA-A*68:02	4.00				
VP3	80	93	14	ETTWTIVNAPINFY	HLA-A*26:01	18.00	HLA-A*68:01	19.00		
VP3	81	88	8	TTWTIVNA	HLA-A*68:02	21.00				
VP3	81	90	10	TTWTIVNAPI	HLA-A*68:02	4.00	HLA-C*14:02	30.00		
VP3	81	92	12	TTWTIVNAPINF	HLA-A*32:01	47.00				
VP3	81	93	13	TTWTIVNAPINFY	HLA-A*11:01	48.00	HLA-A*29:02	12.00	HLA-A*30:02	36.00
VP3	82	90	9	TWTIVNAPI	HLA-C*14:02	37.00				
VP3	82	95	14	TWTIVNAPINFYNY	HLA-A*29:02	41.00				
VP3	83	90	8	WTIVNAPI	HLA-A*68:02	16.00				
VP3	83	95	13	WTIVNAPINFYNY	HLA-A*29:02	7.00	HLA-A*30:02	47.00		
VP3	83	96	14	WTIVNAPINFYNYI	HLA-A*68:02	28.00				
VP3	84	95	12	TIVNAPINFYNY	HLA-A*29:02	27.00				
VP3	84	96	13	TIVNAPINFYNYI	HLA-A*68:02	38.00				
VP3	85	93	9	IVNAPINFY	HLA-A*30:02	25.00				
VP3	85	96	12	IVNAPINFYNYI	HLA-A*68:02	29.00	HLA-C*12:03	30.00		
VP3	88	95	8	APINFYNY	HLA-B*35:01	36.00				
VP3	91	99	9	NFYNYIQQY	HLA-A*29:02	28.00	HLA-C*14:02	36.00		
VP3	91	100	10	NFYNYIQQYY	HLA-A*29:02	12.00	HLA-C*14:02	24.00		
VP3	91	103	13	NFYNYIQQYYSDL	HLA-C*14:02	11.00				
VP3	92	99	8	FYNYIQQY	HLA-A*29:02	30.00	HLA-C*14:02	33.00		
VP3	92	100	9	FYNYIQQYY	HLA-A*29:02	14.00	HLA-C*14:02	27.00		
VP3	92	103	12	FYNYIQQYYSDL	HLA-C*14:02	6.00				
VP3	93	100	8	YNYIQQYY	HLA-A*29:02	12.00				
VP3	93	103	11	YNYIQQYYSDL	HLA-C*03:03	17.00				
VP3	94	103	10	NYIQQYYSDL	HLA-C*14:02	16.00				
VP3	94	106	13	NYIQQYYSDLSPI	HLA-C*14:02	8.00				
VP3	97	106	10	QQYYSDLSPI	HLA-A*02:06	30.00				

VP3	97	110	14	QQYYSDLSPIRPSM	HLA-B*15:01	41.00	HLA-C*14:02	20.00					
VP3	98	106	9	QYYSDLSPI	HLA-A*23:01	38.00	HLA-C*14:02	3.00					
VP3	98	110	13	QYYSDLSPIRPSM	HLA-C*14:02	3.00							
VP3	98	111	14	QYYSDLSPIRPSMV	HLA-C*14:02	4.00							
VP3	99	106	8	YYSDLSPI	HLA-C*12:03	33.00	HLA-C*14:02	3.00					
VP3	99	110	12	YYSDLSPIRPSM	HLA-C*07:02	48.00	HLA-C*14:02	4.00					
VP3	99	111	13	YYSDLSPIRPSMV	HLA-C*12:03	32.00	HLA-C*14:02	7.00					
VP3	100	110	11	YSDLSPIRPSM	HLA-C*05:01	16.00							
VP3	100	111	12	YSDLSPIRPSMV	HLA-A*01:01	30.00	HLA-C*05:01	2.00	HLA-C*12:03	27.00			
VP3	104	114	11	SPIRPSMVRQV	HLA-B*07:02	33.00							
VP3	104	115	12	SPIRPSMVRQVA	HLA-B*07:02	28.00							
VP3	106	114	9	IRPSMVRQV	HLA-C*06:02	29.00	HLA-C*07:01	26.00					
VP3	107	115	9	RPSMVRQVA	HLA-B*07:02	15.00							
VP3	109	117	9	SMVRQVAER	HLA-A*31:01	35.00							
VP3	110	117	8	MVRQVAER	HLA-A*31:01	31.00	HLA-A*33:01	31.00	HLA-A*68:01	20.00			
VP3	110	121	12	MVRQVAEREGTR	HLA-A*33:01	39.00	HLA-A*68:01	45.00					
VP3	112	124	13	RQVAEREGTRVHF	HLA-B*15:01	18.00							
VP3	114	124	11	VAEREGTRVHF	HLA-C*05:01	37.00							
VP3	115	128	14	AEREGTRVHFGHTY	HLA-B*18:01	18.00	HLA-B*44:03	42.00	HLA-B*44:03	42.00			
VP3	117	130	14	REGTRVHFGHTYSI	HLA-B*40:02	38.00							
VP3	119	129	11	GTRVHFGHTYS	HLA-A*30:01	28.00							
VP3	119	130	12	GTRVHFGHTYSI	HLA-A*30:01	10.00							
VP3	120	130	11	TRVHFGHTYSI	HLA-B*39:01	28.00							
VP3	121	128	8	RVHFGHTY	HLA-A*29:02	33.00	HLA-A*30:01	20.00	HLA-A*30:02	38.00	HLA-A*80:01	28.00	
VP3	121	129	9	RVHFGHTYS	HLA-A*30:01	24.00							
VP3	122	130	9	VHFGHTYSI	HLA-B*39:01	15.00	HLA-C*06:02	14.00	HLA-C*07:01	8.00	HLA-C*14:02	42.00	
VP3	126	136	11	HTYSIDDADSI	HLA-A*68:02	12.00	HLA-C*14:02	22.00	HLA-C*15:02	42.00			
VP3	126	139	14	HTYSIDDADSIEEV	HLA-A*68:02	3.00	HLA-C*12:03	45.00	HLA-C*15:02	28.00			
VP3	127	136	10	TYSIDDADSI	HLA-C*14:02	18.00							
VP3	127	139	13	TYSIDDADSIEEV	HLA-C*14:02	34.00							
VP3	128	136	9	YSIDDADSI	HLA-C*03:03	7.00	HLA-C*12:03	7.00	HLA-C*15:02	32.00			

VP3	128	139	12	YSIDDADSIEEV	HLA-A*02:06	13.00	HLA-A*68:02	20.00	HLA-C*03:03	17.00	HLA-C*12:03	6.00	HLA-C*15:02	22.00
VP3	129	139	11	SIDDADSIEEV	HLA-C*05:01	33.00								
VP3	132	142	11	DADSIEEVTQR	HLA-A*68:01	50.00								
VP3	134	142	9	DSIEEVTQR	HLA-A*68:01	17.00								
VP3	134	146	13	DSIEEVTQRMDLR	HLA-A*68:01	19.00								
VP3	138	146	9	EVTQRMDLR	HLA-A*33:01	37.00	HLA-A*68:01	12.00						
VP3	138	151	14	EVTQRMDLRNQQSV	HLA-A*68:02	33.00								
VP3	141	151	11	QRMDLRNQQSV	HLA-C*06:02	23.00	HLA-C*07:01	17.00						
VP3	147	156	10	NQQSVHSGEF	HLA-B*15:01	21.00								
VP3	148	156	9	QQSVHSGEF	HLA-B*15:01	24.00								
VP3	150	159	10	SVHSGEFIEK	HLA-A*11:01	22.00								
VP3	159	169	11	KTIAPGGANQR	HLA-A*31:01	37.00								
VP3	160	169	10	TIAPGGANQR	HLA-A*68:01	31.00								
VP3	161	174	14	IAPGGANQRTAPQW	HLA-B*58:01	48.00								
VP3	168	178	11	QRTAPQWMLPL	HLA-B*39:01	34.00								
VP3	169	178	10	RTAPQWMLPL	HLA-C*15:02	41.00								
VP3	169	179	11	RTAPQWMLPLL	HLA-A*30:01	44.00	HLA-B*58:01	44.00						
VP3	169	180	12	RTAPQWMLPLLL	HLA-B*58:01	30.00								
VP3	169	182	14	RTAPQWMLPLLLGL	HLA-A*02:06	40.00								
VP3	171	179	9	APQWMLPLL	HLA-B*07:02	29.00								
VP3	171	182	12	APQWMLPLLLGL	HLA-B*07:02	21.00								
VP3	174	182	9	WMLPLLLGL	HLA-A*02:01	9.00	HLA-A*02:06	6.00						
VP3	174	183	10	WMLPLLLGLY	HLA-A*29:02	37.00	HLA-A*80:01	45.00	HLA-B*15:01	28.00				
VP3	174	185	12	WMLPLLLGLYGT	HLA-A*02:01	29.00	HLA-A*02:06	19.00						
VP3	174	186	13	WMLPLLLGLYGTV	HLA-A*02:01	14.00	HLA-A*02:06	5.00						
VP3	175	183	9	MLPLLLGLY	HLA-A*29:02	20.00								
VP3	176	183	8	LPLLLGLY	HLA-B*35:01	14.00								
VP3	178	186	9	LLLGLYGTV	HLA-A*02:01	46.00	HLA-A*02:06	27.00						
VP3	178	189	12	LLLGLYGTVTPA	HLA-A*02:01	37.00	HLA-A*02:06	50.00						
VP3	178	190	13	LLLGLYGTVTPAL	HLA-A*02:01	19.00	HLA-A*02:06	34.00	HLA-C*03:03	24.00				
VP3	181	189	9	GLYGTVTPA	HLA-A*02:01	24.00								

VP3	181	190	10	GLYGTVTPAL	HLA-A*02:01	20.00	HLA-C*03:03	24.00	HLA-C*14:02	24.00
VP3	181	193	13	GLYGTVTPALEAY	HLA-A*29:02	24.00	HLA-B*15:01	41.00		
VP3	182	190	9	LYGTVTPAL	HLA-C*14:02	8.00				
VP3	185	193	9	TVTPALEAY	HLA-B*35:01	17.00				
VP3	191	202	12	EAYEDGPNQKKR	HLA-A*68:01	28.00				
VP3	191	203	13	EAYEDGPNQKKRR	HLA-A*68:01	40.00				
VP3	191	204	14	EAYEDGPNQKKRRV	HLA-C*12:03	11.00				
VP3	198	206	9	NQKKRRVSR	HLA-A*31:01	17.00				
VP3	200	211	12	KKRRVSRGSSQK	HLA-A*30:01	12.00				
VP3	200	213	14	KKRRVSRGSSQKAK	HLA-A*30:01	19.00				
VP3	201	211	11	KRRVSRGSSQK	HLA-A*30:01	18.00				
VP3	201	213	13	KRRVSRGSSQKAK	HLA-A*30:01	27.00				
VP3	202	211	10	RRVSRGSSQK	HLA-B*27:05	36.00				
VP3	202	213	12	RRVSRGSSQKAK	HLA-B*27:05	42.00				
VP3	203	211	9	RVSRGSSQK	HLA-A*03:01	47.00	HLA-A*11:01	23.00	HLA-A*30:01	25.00
VP3	203	213	11	RVSRGSSQKAK	HLA-A*30:01	36.00				
VP3	203	216	14	RVSRGSSQKAKGTR	HLA-A*31:01	42.00				
VP3	204	211	8	VSRGSSQK	HLA-A*30:01	14.00				
VP3	209	216	8	SQKAKGTR	HLA-A*31:01	36.00				
VP3	211	219	9	KAKGTRASA	HLA-A*30:01	6.00				
VP3	211	224	14	KAKGTRASAKTTNK	HLA-A*30:01	5.00				
VP3	214	224	11	GTRASAKTTNK	HLA-A*30:01	6.00				
VP3	216	226	11	RASAKTTNKRR	HLA-A*31:01	44.00				
VP3	216	228	13	RASAKTTNKRRSR	HLA-A*31:01	25.00				
VP3	217	224	8	ASAKTTNK	HLA-A*11:01	30.00				
VP3	218	231	14	SAKTTNKRRSRSSR	HLA-A*31:01	42.00				
VP3	220	228	9	KTTNKRRSR	HLA-A*31:01	17.00				
VP3	220	231	12	KTTNKRRSRSSR	HLA-A*31:01	14.00				
VP3	221	228	8	TTNKRRSR	HLA-A*31:01	23.00	HLA-A*33:01	25.00	HLA-A*68:01	35.00
VP3	221	231	11	TTNKRRSRSSR	HLA-A*31:01	15.00	HLA-A*33:01	18.00	HLA-A*68:01	14.00
LTA	6	13	8	NREESMEL	HLA-C*07:01	33.00				

LTA	6	14	9	NREESMELM	HLA-C*07:01	30.00						
LTA	6	17	12	NREESMELMDLL	HLA-C*07:01	49.00						
LTA	7	14	8	REESMELM	HLA-B*40:01	28.00						
LTA	7	17	11	REESMELMDLL	HLA-B*40:01	7.00						
LTA	7	19	13	REESMELMDLLGL	HLA-B*40:01	7.00						
LTA	9	17	9	ESMELMDLL	HLA-A*68:02	7.00						
LTA	9	19	11	ESMELMDLLGL	HLA-A*68:02	23.00						
LTA	9	21	13	ESMELMDLLGLER	HLA-A*33:01	35.00	HLA-A*68:01	18.00				
LTA	9	22	14	ESMELMDLLGLERA	HLA-A*68:02	26.00						
LTA	11	19	9	MELMDLLGL	HLA-B*40:01	20.00	HLA-B*40:02	28.00				
LTA	11	24	14	MELMDLLGLERAAW	HLA-B*18:01	24.00	HLA-B*44:02	18.00	HLA-B*44:03	22.00	HLA-B*44:03	22.00
LTA	12	21	10	ELMDLLGLER	HLA-A*33:01	48.00	HLA-A*68:01	34.00				
LTA	19	29	11	LERAAWGNLPL	HLA-B*40:01	28.00	HLA-B*40:02	40.00				
LTA	20	29	10	ERAAWGNLPL	HLA-B*39:01	7.00						
LTA	21	29	9	RAAWGNLPL	HLA-B*07:02	10.00	HLA-C*03:03	6.00				
LTA	21	30	10	RAAWGNLPLM	HLA-C*03:03	38.00						
LTA	21	31	11	RAAWGNLPLMR	HLA-A*31:01	37.00						
LTA	21	32	12	RAAWGNLPLMRK	HLA-A*03:01	43.00	HLA-A*11:01	25.00				
LTA	21	34	14	RAAWGNLPLMRKAY	HLA-B*35:01	26.00						
LTA	22	29	8	AAWGNLPL	HLA-C*03:03	7.00						
LTA	22	30	9	AAWGNLPLM	HLA-C*03:03	41.00						
LTA	22	34	13	AAWGNLPLMRKAY	HLA-B*35:01	44.00						
LTA	22	35	14	AAWGNLPLMRKAYL	HLA-C*03:03	10.00						
LTA	27	34	8	LPLMRKAY	HLA-B*35:01	8.00						
LTA	27	35	9	LPLMRKAYL	HLA-B*07:02	35.00	HLA-B*08:01	13.00				
LTA	29	36	8	LMRKAYLR	HLA-A*31:01	13.00						
LTA	29	37	9	LMRKAYLRK	HLA-A*03:01	27.00	HLA-A*30:01	7.00				
LTA	29	39	11	LMRKAYLRKCK	HLA-A*30:01	19.00						
LTA	29	41	13	LMRKAYLRKCKEF	HLA-B*15:01	30.00						
LTA	32	39	8	KAYLRKCK	HLA-A*30:01	50.00						
LTA	32	45	14	KAYLRKCKEFHPDK	HLA-A*03:01	45.00	HLA-A*30:01	25.00				

LTA	33	41	9	AYLRKCKEF	HLA-C*14:02	13.00				
LTA	37	45	9	KCKEFHPDK	HLA-A*30:01	6.00				
LTA	41	52	12	FHPDKGGDEDKM	HLA-C*07:01	31.00	HLA-C*14:02	45.00		
LTA	48	59	12	DEDKMKRMNTLY	HLA-B*18:01	14.00				
LTA	51	58	8	KMKRMNTL	HLA-A*30:01	26.00				
LTA	51	59	9	KMKRMNTLY	HLA-A*30:01	33.00	HLA-A*30:02	33.00	HLA-A*80:01	46.00
LTA	51	60	10	KMKRMNTLYK	HLA-A*03:01	14.00	HLA-A*11:01	28.00	HLA-A*31:01	16.00
LTA	51	61	11	KMKRMNTLYKK	HLA-A*30:01	5.00	HLA-A*31:01	8.00		
LTA	52	60	9	MKRMNTLYK	HLA-A*30:01	7.00				
LTA	53	61	9	KRMNTLYKK	HLA-B*27:05	17.00				
LTA	53	62	10	KRMNTLYKKM	HLA-B*27:05	28.00				
LTA	54	61	8	RMNTLYKK	HLA-A*03:01	40.00	HLA-A*11:01	26.00	HLA-A*31:01	10.00
LTA	62	70	9	MEQDVKVAH	HLA-B*18:01	41.00				
LTA	62	74	13	MEQDVKVAHQPDF	HLA-B*18:01	19.00	HLA-B*44:02	40.00		
LTA	67	77	11	KVAHQPDFGTW	HLA-B*58:01	12.00				
LTA	70	82	13	HQPDFGTWSSSEV	HLA-A*02:06	23.00				
LTA	74	85	12	FGTWSSSEVPTY	HLA-B*35:01	9.00				
LTA	77	85	9	WSSSEVPTY	HLA-B*35:01	13.00				
LTA	77	90	14	WSSSEVPTYGTEEW	HLA-B*58:01	8.00				
LTA	78	90	13	SSSEVPTYGTEEW	HLA-B*58:01	8.00				
LTA	80	90	11	SEVPTYGTEEW	HLA-B*44:02	28.00	HLA-B*44:03	24.00	HLA-B*44:03	24.00
LTA	80	93	14	SEVPTYGTEEWESW	HLA-B*44:02	32.00	HLA-B*44:03	26.00	HLA-B*44:03	26.00
LTA	82	90	9	VPTYGTEEW	HLA-B*53:01	15.00				
LTA	82	93	12	VPTYGTEEWESW	HLA-B*53:01	20.00				
LTA	82	94	13	VPTYGTEEWESWW	HLA-B*53:01	22.00				
LTA	84	97	14	TYGTEEWESWWSSF	HLA-A*23:01	38.00	HLA-A*24:02	39.00	HLA-C*14:02	50.00
LTA	87	94	8	TEEWESWW	HLA-B*44:02	38.00				
LTA	88	96	9	EEWESWWSS	HLA-B*40:02	50.00				
LTA	88	97	10	EEWESWWSSF	HLA-B*44:03	43.00	HLA-B*44:03	43.00		
LTA	88	101	14	EEWESWWSSFNEKW	HLA-B*44:03	15.00	HLA-B*44:03	15.00		
LTA	90	97	8	WESWWSSF	HLA-B*18:01	45.00	HLA-B*40:01	30.00		

LTA	90	101	12	WESWWSSFNEKW	HLA-B*44:02	47.00	HLA-B*44:03	26.00	HLA-B*44:03	26.00
LTA	91	100	10	ESWWSSFNEK	HLA-A*68:01	33.00				
LTA	91	101	11	ESWWSSFNEKW	HLA-B*58:01	13.00				
LTA	94	101	8	WSSFNEKW	HLA-B*58:01	14.00				
LTA	112	120	9	FASDEEATA	HLA-C*12:03	28.00				
LTA	118	129	12	ATADSQHSTPPK	HLA-A*11:01	14.00	HLA-A*30:01	21.00		
LTA	118	130	13	ATADSQHSTPPKK	HLA-A*11:01	20.00				
LTA	118	131	14	ATADSQHSTPPKKK	HLA-A*11:01	36.00				
LTA	122	129	8	SQHSTPPK	HLA-A*11:01	34.00	HLA-A*30:01	10.00		
LTA	122	130	9	SQHSTPPKK	HLA-A*30:01	33.00				
LTA	140	147	8	FPSDLHQF	HLA-B*35:01	5.00	HLA-B*53:01	8.00		
LTA	140	148	9	FPSDLHQFL	HLA-B*35:01	18.00	HLA-B*53:01	42.00	HLA-C*03:03	34.00
LTA	140	152	13	FPSDLHQFLSQAV	HLA-B*35:01	15.00	HLA-C*12:03	28.00		
LTA	140	153	14	FPSDLHQFLSQAVF	HLA-B*35:01	4.00	HLA-B*35:03	50.00	HLA-B*53:01	10.00
LTA	141	153	13	PSDLHQFLSQAVF	HLA-C*05:01	17.00				
LTA	143	156	14	DLHQFLSQAVFSNR	HLA-A*33:01	17.00	HLA-A*68:01	47.00		
LTA	145	152	8	HQFLSQAV	HLA-A*02:06	41.00	HLA-B*39:01	31.00		
LTA	145	153	9	HQFLSQAVF	HLA-B*15:01	24.00				
LTA	145	156	12	HQFLSQAVFSNR	HLA-A*31:01	16.00				
LTA	145	158	14	HQFLSQAVFSNRTL	HLA-B*39:01	21.00				
LTA	146	156	11	QFLSQAVFSNR	HLA-A*33:01	33.00				
LTA	146	158	13	QFLSQAVFSNRTL	HLA-C*14:02	28.00				
LTA	147	156	10	FLSQAVFSNR	HLA-A*68:01	31.00				
LTA	147	158	12	FLSQAVFSNRTL	HLA-A*02:01	37.00	HLA-C*03:03	11.00		
LTA	148	156	9	LSQAVFSNR	HLA-A*31:01	15.00				
LTA	148	161	14	LSQAVFSNRTLACF	HLA-B*15:01	38.00				
LTA	149	156	8	SQAVFSNR	HLA-A*31:01	33.00				
LTA	149	161	13	SQAVFSNRTLACF	HLA-B*15:01	24.00				
LTA	149	162	14	SQAVFSNRTLACFA	HLA-A*02:06	31.00				
LTA	150	158	9	QAVFSNRTL	HLA-C*03:03	4.00				
LTA	151	163	13	AVFSNRTLACFAV	HLA-A*02:06	19.00	HLA-A*68:02	28.00		

LTA	151	164	14	AVFSNRTLACFAVY	HLA-A*29:02	16.00	HLA-A*30:02	12.00	HLA-B*15:01	47.00					
LTA	152	164	13	VFSNRTLACFAVY	HLA-A*29:02	32.00									
LTA	153	164	12	FSNRTLACFAVY	HLA-A*01:01	25.00	HLA-A*30:02	39.00	HLA-B*15:01	26.00	HLA-B*35:01	30.00	HLA-C*12:03	31.00
LTA	156	163	8	RTLACFAV	HLA-A*02:06	4.00								
LTA	156	164	9	RTLACFAVY	HLA-A*30:02	8.00	HLA-A*80:01	23.00	HLA-B*15:01	38.00	HLA-B*58:01	32.00		
LTA	156	167	12	RTLACFAVYTTK	HLA-A*03:01	30.00	HLA-A*11:01	16.00	HLA-A*30:01	8.00	HLA-A*31:01	10.00		
LTA	156	169	14	RTLACFAVYTTKEK	HLA-A*11:01	27.00	HLA-A*30:01	13.00	HLA-A*31:01	50.00				
LTA	157	164	8	TLACFAVY	HLA-A*29:02	33.00								
LTA	157	167	11	TLACFAVYTTK	HLA-A*68:01	35.00								
LTA	160	173	14	CFAVYTTKEKAQIL	HLA-C*14:02	48.00								
LTA	161	169	9	FAVYTTKEK	HLA-A*68:01	39.00								
LTA	161	170	10	FAVYTTKEKA	HLA-C*03:03	41.00								
LTA	161	171	11	FAVYTTKEKAQ	HLA-C*03:03	36.00								
LTA	161	172	12	FAVYTTKEKAQI	HLA-C*03:03	3.00	HLA-C*12:03	19.00						
LTA	161	173	13	FAVYTTKEKAQIL	HLA-C*03:03	1.00	HLA-C*12:03	15.00						
LTA	161	174	14	FAVYTTKEKAQILY	HLA-A*29:02	33.00	HLA-B*35:01	4.00	HLA-C*03:03	9.00	HLA-C*12:03	45.00		
LTA	162	169	8	AVYTTKEK	HLA-A*11:01	33.00								
LTA	162	173	12	AVYTTKEKAQIL	HLA-C*03:03	21.00								
LTA	162	174	13	AVYTTKEKAQILY	HLA-A*29:02	28.00								
LTA	162	175	14	AVYTTKEKAQILYK	HLA-A*03:01	30.00	HLA-A*11:01	14.00	HLA-A*30:01	48.00				
LTA	163	172	10	VYTTKEKAQI	HLA-C*14:02	16.00								
LTA	163	173	11	VYTTKEKAQIL	HLA-C*14:02	7.00								
LTA	164	173	10	YTTKEKAQIL	HLA-C*03:03	19.00								
LTA	164	174	11	YTTKEKAQILY	HLA-A*01:01	48.00	HLA-A*80:01	29.00						
LTA	164	175	12	YTTKEKAQILYK	HLA-A*11:01	46.00	HLA-A*68:01	18.00						
LTA	164	176	13	YTTKEKAQILYKK	HLA-A*68:01	18.00								
LTA	164	177	14	YTTKEKAQILYKKL	HLA-C*03:03	23.00								
LTA	165	175	11	TTKEKAQILYK	HLA-A*30:01	10.00								
LTA	167	175	9	KEKAQILYK	HLA-A*30:01	29.00								
LTA	170	183	14	AQILYKKLMEKYSV	HLA-A*02:06	30.00								
LTA	172	180	9	ILYKKLMEK	HLA-A*03:01	12.00	HLA-A*11:01	33.00						

LTA	172	183	12	ILYKKLMEKYSV	HLA-A*02:01	10.00	HLA-A*02:06	35.00		
LTA	172	185	14	ILYKKLMEKYSVTF	HLA-A*32:01	18.00	HLA-B*15:01	43.00		
LTA	173	185	13	LYKKLMEKYSVTF	HLA-A*23:01	46.00	HLA-A*24:02	40.00	HLA-C*14:02	29.00
LTA	173	186	14	LYKKLMEKYSVTFI	HLA-C*14:02	15.00				
LTA	176	183	8	KLMEKYSV	HLA-A*02:06	4.00				
LTA	176	185	10	KLMEKYSVTF	HLA-B*15:01	38.00				
LTA	176	186	11	KLMEKYSVTFI	HLA-A*02:01	23.00	HLA-A*32:01	29.00		
LTA	176	188	13	KLMEKYSVTFISR	HLA-A*31:01	9.00				
LTA	177	185	9	LMEKYSVTF	HLA-B*15:01	47.00				
LTA	179	188	10	EKYSVTFISR	HLA-A*33:01	50.00				
LTA	180	188	9	KYSVTFISR	HLA-A*31:01	9.00				
LTA	180	192	13	KYSVTFISRHMCA	HLA-A*30:01	49.00				
LTA	181	188	8	YSVTFISR	HLA-A*68:01	10.00				
LTA	181	190	10	YSVTFISRHM	HLA-C*03:03	9.00	HLA-C*12:03	29.00		
LTA	184	197	14	TFISRHMCAGHNII	HLA-C*14:02	40.00				
LTA	185	196	12	FISRHMCAGHNI	HLA-A*02:06	34.00	HLA-C*12:03	39.00		
LTA	185	197	13	FISRHMCAGHNII	HLA-C*12:03	19.00				
LTA	188	197	10	RHMCAGHNII	HLA-B*39:01	34.00				
LTA	188	200	13	RHMCAGHNIIFFL	HLA-B*38:01	39.00	HLA-B*39:01	41.00		
LTA	189	199	11	HMCAGHNIIFF	HLA-B*15:01	41.00				
LTA	190	198	9	MCAGHNIIF	HLA-B*35:01	50.00				
LTA	190	200	11	MCAGHNIIFFL	HLA-A*68:02	15.00				
LTA	194	204	11	HNIIFFLTPHR	HLA-A*68:01	40.00				
LTA	194	206	13	HNIIFFLTPHRHR	HLA-A*33:01	46.00				
LTA	195	204	10	NIIFFLTPHR	HLA-A*68:01	8.00				
LTA	195	206	12	NIIFFLTPHRHR	HLA-A*33:01	16.00	HLA-A*68:01	19.00		
LTA	195	207	13	NIIFFLTPHRHRV	HLA-A*68:02	9.00				
LTA	196	204	9	IIFFLTPHR	HLA-A*31:01	32.00	HLA-A*33:01	46.00	HLA-A*68:01	10.00
LTA	196	206	11	IIFFLTPHRHR	HLA-A*31:01	24.00	HLA-A*33:01	23.00	HLA-A*68:01	23.00
LTA	196	207	12	IIFFLTPHRHRV	HLA-A*02:01	45.00	HLA-A*68:02	39.00		
LTA	197	204	8	IFFLTPHR	HLA-A*31:01	21.00	HLA-A*33:01	14.00		

LTA	197	206	10	IFFLTPHRHR	HLA-A*31:01	27.00	HLA-A*33:01	29.00				
LTA	197	210	14	IFFLTPHRHRVSAI	HLA-C*14:02	24.00						
LTA	198	206	9	FFLTPHRHR	HLA-A*33:01	9.00						
LTA	198	210	13	FFLTPHRHRVSAI	HLA-C*14:02	13.00						
LTA	199	207	9	FLTPHRHRV	HLA-A*02:01	23.00	HLA-A*02:06	9.00	HLA-C*12:03	37.00		
LTA	199	210	12	FLTPHRHRVSAI	HLA-A*02:01	48.00	HLA-A*02:06	23.00	HLA-C*12:03	22.00	HLA-C*14:02	27.00
LTA	201	209	9	TPHRHRVSA	HLA-B*07:02	21.00	HLA-B*08:01	19.00				
LTA	201	210	10	TPHRHRVSAI	HLA-B*07:02	25.00						
LTA	203	210	8	HRHRVSAI	HLA-B*39:01	17.00						
LTA	204	216	13	RHRVSAINNFCQK	HLA-A*30:01	14.00						
LTA	205	217	13	HRVSAINNFCQKL	HLA-B*39:01	49.00						
LTA	206	213	8	RVSAINNF	HLA-A*32:01	38.00						
LTA	206	216	11	RVSAINNFCQK	HLA-A*30:01	11.00	HLA-A*31:01	35.00				
LTA	207	220	14	VSAINNFCQKLCTF	HLA-B*58:01	29.00						
LTA	208	216	9	SAINNFCQK	HLA-A*11:01	19.00	HLA-A*68:01	22.00				
LTA	208	217	10	SAINNFCQKL	HLA-C*03:03	7.00						
LTA	208	220	13	SAINNFCQKLCTF	HLA-C*03:03	12.00						
LTA	209	216	8	AINNFCQK	HLA-A*11:01	17.00						
LTA	209	220	12	AINNFCQKLCTF	HLA-A*32:01	48.00						
LTA	209	222	14	AINNFCQKLCTFSF	HLA-A*32:01	22.00						
LTA	216	224	9	KLCTFSFLI	HLA-A*02:01	11.00	HLA-A*32:01	14.00				
LTA	216	226	11	KLCTFSFLICK	HLA-A*30:01	45.00						
LTA	218	226	9	CTFSFLICK	HLA-A*03:01	43.00	HLA-A*11:01	12.00	HLA-A*30:01	27.00	HLA-A*68:01	6.00
LTA	218	228	11	CTFSFLICKGV	HLA-A*68:02	23.00						
LTA	218	230	13	CTFSFLICKGVNK	HLA-A*03:01	41.00	HLA-A*11:01	15.00	HLA-A*68:01	12.00		
LTA	220	228	9	FSFLICKGV	HLA-A*68:02	39.00						
LTA	220	230	11	FSFLICKGVNK	HLA-A*68:01	20.00						
LTA	220	233	14	FSFLICKGVNKEYL	HLA-A*68:02	43.00	HLA-C*03:03	17.00				
LTA	222	233	12	FLICKGVNKEYL	HLA-A*02:01	21.00	HLA-A*02:06	33.00	HLA-C*03:03	8.00		
LTA	222	234	13	FLICKGVNKEYLL	HLA-A*02:01	9.00	HLA-A*02:06	35.00	HLA-C*03:03	20.00		
LTA	222	235	14	FLICKGVNKEYLLY	HLA-A*29:02	5.00	HLA-A*80:01	31.00				

LTA	223	235	13	LICKGVNKEYLLY	HLA-A*29:02	25.00	HLA-A*80:01	26.00				
LTA	227	235	9	GVNKEYLLY	HLA-A*29:02	7.00	HLA-A*80:01	14.00				
LTA	229	238	10	NKEYLLYSAL	HLA-B*39:01	45.00						
LTA	230	237	8	KEYLLYSA	HLA-B*40:02	6.00						
LTA	230	238	9	KEYLLYSAL	HLA-B*40:01	6.00	HLA-B*40:02	4.00				
LTA	230	240	11	KEYLLYSALTR	HLA-B*40:02	43.00						
LTA	230	243	14	KEYLLYSALTRDPY	HLA-B*18:01	14.00	HLA-B*40:02	44.00				
LTA	231	238	8	EYLLYSAL	HLA-C*14:02	11.00						
LTA	231	240	10	EYLLYSALTR	HLA-A*33:01	10.00						
LTA	232	243	12	YLLYSALTRDPY	HLA-A*29:02	12.00	HLA-B*15:01	45.00	HLA-C*14:02	49.00		
LTA	232	245	14	YLLYSALTRDPYHT	HLA-A*02:01	31.00						
LTA	233	243	11	LLYSALTRDPY	HLA-B*15:01	27.00						
LTA	233	246	14	LLYSALTRDPYHTI	HLA-A*02:01	20.00	HLA-A*02:06	40.00	HLA-C*03:03	31.00	HLA-C*12:03	25.00
LTA	234	243	10	LYSALTRDPY	HLA-C*14:02	28.00						
LTA	234	246	13	LYSALTRDPYHTI	HLA-A*24:02	21.00	HLA-C*14:02	9.00				
LTA	235	243	9	YSALTRDPY	HLA-A*01:01	26.00	HLA-B*15:01	46.00	HLA-B*35:01	31.00		
LTA	235	246	12	YSALTRDPYHTI	HLA-A*68:02	50.00	HLA-C*03:03	40.00	HLA-C*12:03	18.00		
LTA	236	243	8	SALTRDPY	HLA-B*35:01	41.00						
LTA	236	246	11	SALTRDPYHTI	HLA-C*12:03	25.00						
LTA	238	246	9	LTRDPYHTI	HLA-A*30:01	32.00	HLA-C*12:03	12.00				
LTA	239	246	8	TRDPYHTI	HLA-B*39:01	37.00	HLA-C*07:01	48.00				
LTA	241	250	10	DPYHTIEESI	HLA-C*12:03	44.00						
LTA	242	250	9	PYHTIEESI	HLA-C*14:02	9.00						
LTA	242	254	13	PYHTIEESIQGGL	HLA-C*14:02	17.00						
LTA	244	254	11	HTIEESIQGGL	HLA-A*26:01	40.00	HLA-A*68:02	24.00				
LTA	244	255	12	HTIEESIQGGLK	HLA-A*11:01	40.00	HLA-A*68:01	21.00				
LTA	246	254	9	IEESIQGGL	HLA-B*40:01	39.00						
LTA	259	270	12	FSPEEPEETKQV	HLA-C*12:03	21.00						
LTA	265	272	8	EETKQVSW	HLA-B*44:02	25.00	HLA-B*44:03	49.00	HLA-B*44:03	49.00		
LTA	265	278	14	EETKQVSWKLITEY	HLA-B*44:03	27.00	HLA-B*44:03	27.00				
LTA	266	273	8	ETKQVSWK	HLA-A*68:01	15.00						

LTA	266	274	9	ETKQVSWKL	HLA-A*68:02	45.00				
LTA	266	278	13	ETKQVSWKLITEY	HLA-A*26:01	6.00				
LTA	266	279	14	ETKQVSWKLITEYA	HLA-A*68:02	15.00				
LTA	268	278	11	KQVSWKLITEY	HLA-B*15:01	39.00				
LTA	268	279	12	KQVSWKLITEYA	HLA-A*02:06	28.00				
LTA	268	280	13	KQVSWKLITEYAV	HLA-A*02:06	10.00				
LTA	273	280	8	KLITEYAV	HLA-A*02:06	10.00				
LTA	276	288	13	TEYAVETKCEDVF	HLA-B*18:01	26.00				
LTA	276	289	14	TEYAVETKCEDVFL	HLA-B*40:01	12.00				
LTA	278	287	10	YAVETKCEDV	HLA-C*03:03	8.00	HLA-C*12:03	18.00		
LTA	278	288	11	YAVETKCEDVF	HLA-B*35:01	5.00	HLA-C*03:03	4.00	HLA-C*12:03	14.00
LTA	278	289	12	YAVETKCEDVFL	HLA-A*02:06	39.00	HLA-C*03:03	2.00	HLA-C*12:03	12.00
LTA	278	290	13	YAVETKCEDVFLL	HLA-A*02:06	20.00	HLA-C*03:03	2.00	HLA-C*12:03	11.00
LTA	278	291	14	YAVETKCEDVFLLL	HLA-A*02:06	26.00	HLA-C*03:03	2.00	HLA-C*12:03	8.00
LTA	280	290	11	VETKCEDVFLL	HLA-B*40:01	32.00				
LTA	280	291	12	VETKCEDVFLLL	HLA-B*40:01	29.00				
LTA	281	289	9	ETKCEDVFL	HLA-A*68:02	40.00				
LTA	281	294	14	ETKCEDVFLLLGMY	HLA-A*26:01	12.00				
LTA	284	291	8	CEDVFLLL	HLA-B*40:01	28.00				
LTA	284	295	12	CEDVFLLLGMYL	HLA-B*40:01	21.00				
LTA	286	294	9	DVFLLLGMY	HLA-A*25:01	45.00	HLA-A*26:01	11.00		
LTA	286	299	14	DVFLLLGMYLEFQY	HLA-A*29:02	36.00				
LTA	287	294	8	VFLLLGMY	HLA-A*29:02	9.00				
LTA	287	295	9	VFLLLGMYL	HLA-C*14:02	47.00				
LTA	287	297	11	VFLLLGMYLEF	HLA-A*23:01	31.00				
LTA	287	299	13	VFLLLGMYLEFQY	HLA-A*29:02	5.00				
LTA	288	295	8	FLLLGMYL	HLA-A*02:06	13.00	HLA-C*03:03	14.00		
LTA	288	299	12	FLLLGMYLEFQY	HLA-A*29:02	3.00				
LTA	288	301	14	FLLLGMYLEFQYNV	HLA-A*02:01	3.00	HLA-A*02:06	5.00		
LTA	289	297	9	LLLGMYLEF	HLA-A*29:02	50.00				
LTA	289	299	11	LLLGMYLEFQY	HLA-A*29:02	33.00				

LTA	289	301	13	LLLGMYLEFQYNV	HLA-A*02:01	5.00	HLA-A*02:06	13.00						
LTA	290	301	12	LLGMYLEFQYNV	HLA-A*02:01	10.00								
LTA	292	299	8	GMYLEFQY	HLA-A*29:02	6.00								
LTA	292	301	10	GMYLEFQYNV	HLA-A*02:01	11.00	HLA-A*02:06	49.00						
LTA	292	305	14	GMYLEFQYNVEECK	HLA-A*11:01	43.00								
LTA	297	304	8	FQYNVEEC	HLA-A*02:06	33.00								
LTA	307	314	8	CQKKDQPY	HLA-B*15:01	50.00								
LTA	307	316	10	CQKKDQPYHF	HLA-B*15:01	41.00								
LTA	313	323	11	PYHFKYHEKHF	HLA-C*14:02	21.00								
LTA	314	323	10	YHFKYHEKHF	HLA-C*06:02	43.00	HLA-C*07:01	23.00						
LTA	314	327	14	YHFKYHEKHFANAI	HLA-B*39:01	16.00	HLA-C*06:02	32.00	HLA-C*07:01	11.00	HLA-C*12:03	21.00	HLA-C*14:02	10.00
LTA	315	327	13	HFKYHEKHFANAI	HLA-C*14:02	27.00								
LTA	316	327	12	FKYHEKHFANAI	HLA-C*03:03	23.00	HLA-C*12:03	21.00	HLA-C*14:02	43.00				
LTA	316	328	13	FKYHEKHFANAII	HLA-C*03:03	33.00	HLA-C*12:03	30.00						
LTA	316	329	14	FKYHEKHFANAIIF	HLA-C*03:03	44.00								
LTA	317	324	8	KYHEKHFA	HLA-A*30:01	11.00								
LTA	317	327	11	KYHEKHFANAI	HLA-A*30:01	36.00	HLA-C*14:02	4.00						
LTA	317	328	12	KYHEKHFANAII	HLA-A*30:01	30.00	HLA-C*14:02	6.00						
LTA	317	329	13	KYHEKHFANAIIF	HLA-C*07:02	50.00	HLA-C*14:02	8.00						
LTA	317	330	14	KYHEKHFANAIIFA	HLA-A*30:01	6.00								
LTA	318	327	10	YHEKHFANAI	HLA-B*39:01	15.00	HLA-C*14:02	43.00						
LTA	318	328	11	YHEKHFANAII	HLA-B*39:01	28.00								
LTA	319	327	9	HEKHFANAI	HLA-B*40:01	31.00								
LTA	319	329	11	HEKHFANAIIF	HLA-B*40:01	46.00								
LTA	323	330	8	FANAIIFA	HLA-A*68:02	29.00								
LTA	323	333	11	FANAIIFAESK	HLA-A*68:01	14.00								
LTA	323	336	14	FANAIIFAESKNQK	HLA-A*68:01	20.00								
LTA	325	333	9	NAIIFAESK	HLA-A*68:01	12.00								
LTA	325	336	12	NAIIFAESKNQK	HLA-A*68:01	19.00								
LTA	325	338	14	NAIIFAESKNQKSI	HLA-C*12:03	28.00								
LTA	326	333	8	AIIFAESK	HLA-A*11:01	26.00								

LTA	329	338	10	FAESKNQKSI	HLA-C*03:03	17.00	HLA-C*12:03	8.00		
LTA	330	338	9	AESKNQKSI	HLA-B*44:02	37.00				
LTA	330	343	14	AESKNQKSICQQAV	HLA-B*40:02	41.00	HLA-B*44:02	44.00		
LTA	336	343	8	KSICQQAV	HLA-C*15:02	35.00				
LTA	336	346	11	KSICQQAVDTV	HLA-C*15:02	31.00				
LTA	336	347	12	KSICQQAVDTVL	HLA-B*58:01	28.00	HLA-C*15:02	42.00		
LTA	336	349	14	KSICQQAVDTVLAK	HLA-A*11:01	24.00	HLA-A*30:01	11.00		
LTA	337	349	13	SICQQAVDTVLAK	HLA-A*11:01	27.00				
LTA	337	350	14	SICQQAVDTVLAKK	HLA-A*11:01	37.00				
LTA	341	352	12	QAVDTVLAKKRV	HLA-C*12:03	37.00				
LTA	342	349	8	AVDTVLAK	HLA-A*11:01	23.00				
LTA	342	350	9	AVDTVLAKK	HLA-A*11:01	26.00				
LTA	344	351	8	DTVLAKKR	HLA-A*68:01	39.00				
LTA	347	355	9	LAKKRVDTL	HLA-B*08:01	24.00	HLA-C*03:03	21.00	HLA-C*12:03	15.00
LTA	347	357	11	LAKKRVDTLHM	HLA-C*12:03	28.00				
LTA	347	359	13	LAKKRVDTLHMTR	HLA-A*31:01	28.00				
LTA	351	359	9	RVDTLHMTR	HLA-A*31:01	18.00				
LTA	351	363	13	RVDTLHMTREEML	HLA-C*05:01	40.00				
LTA	353	366	14	DTLHMTREEMLTER	HLA-A*33:01	11.00	HLA-A*68:01	39.00		
LTA	354	362	9	TLHMTREEM	HLA-C*14:02	14.00				
LTA	354	363	10	TLHMTREEML	HLA-C*14:02	34.00				
LTA	354	367	14	TLHMTREEMLTERF	HLA-C*14:02	50.00				
LTA	355	362	8	LHMTREEM	HLA-C*07:01	15.00	HLA-C*14:02	50.00		
LTA	355	363	9	LHMTREEML	HLA-C*07:01	13.00				
LTA	357	366	10	MTREEMLTER	HLA-A*68:01	33.00				
LTA	357	370	14	MTREEMLTERFNHI	HLA-A*30:01	12.00	HLA-C*12:03	16.00		
LTA	358	370	13	TREEMLTERFNHI	HLA-C*07:01	38.00				
LTA	358	371	14	TREEMLTERFNHIL	HLA-B*39:01	18.00	HLA-C*06:02	40.00	HLA-C*07:01	23.00
LTA	359	371	13	REEMLTERFNHIL	HLA-B*40:01	4.00	HLA-B*40:02	42.00		
LTA	360	367	8	EEMLTERF	HLA-B*44:02	47.00	HLA-B*44:03	33.00	HLA-B*44:03	33.00
LTA	360	371	12	EEMLTERFNHIL	HLA-B*40:01	48.00				

LTA	362	370	9	MLTERFNHI	HLA-A*02:01	36.00	HLA-A*02:06	5.00	HLA-B*08:01	26.00	HLA-C*12:03	37.00
LTA	362	373	12	MLTERFNHILDK	HLA-A*03:01	47.00						
LTA	364	371	8	TERFNHIL	HLA-B*40:02	40.00						
LTA	365	374	10	ERFNHILDKM	HLA-C*07:01	34.00						
LTA	365	376	12	ERFNHILDKMDL	HLA-C*06:02	30.00	HLA-C*07:01	27.00				
LTA	365	377	13	ERFNHILDKMDLI	HLA-C*06:02	15.00	HLA-C*07:01	12.00				
LTA	369	380	12	HILDKMDLIFGA	HLA-A*02:06	33.00						
LTA	370	378	9	ILDKMDLIF	HLA-C*05:01	5.00						
LTA	373	380	8	KMDLIFGA	HLA-A*02:06	37.00						
LTA	373	385	13	KMDLIFGAHGNAV	HLA-A*02:01	50.00	HLA-A*02:06	13.00				
LTA	376	385	10	LIFGAHGNAV	HLA-A*02:06	26.00						
LTA	376	386	11	LIFGAHGNAVL	HLA-C*03:03	27.00						
LTA	376	389	14	LIFGAHGNAVLEQY	HLA-A*29:02	16.00						
LTA	377	389	13	IFGAHGNAVLEQY	HLA-A*29:02	22.00						
LTA	377	390	14	IFGAHGNAVLEQYM	HLA-C*14:02	40.00						
LTA	378	386	9	FGAHGNAVL	HLA-C*03:03	4.00						
LTA	378	390	13	FGAHGNAVLEQYM	HLA-C*03:03	16.00						
LTA	379	386	8	GAHGNAVL	HLA-C*03:03	9.00						
LTA	379	390	12	GAHGNAVLEQYM	HLA-C*03:03	38.00						
LTA	380	390	11	AHGNAVLEQYM	HLA-C*07:01	46.00						
LTA	383	390	8	NAVLEQYM	HLA-C*03:03	35.00						
LTA	383	396	14	NAVLEQYMAGVAWL	HLA-C*03:03	42.00						
LTA	384	393	10	AVLEQYMAGV	HLA-A*02:06	9.00						
LTA	384	396	13	AVLEQYMAGVAWL	HLA-A*02:06	24.00						
LTA	385	393	9	VLEQYMAGV	HLA-A*02:01	38.00						
LTA	386	395	10	LEQYMAGVAW	HLA-B*44:02	29.00						
LTA	386	396	11	LEQYMAGVAWL	HLA-B*40:01	21.00						
LTA	387	399	13	EQYMAGVAWLHCL	HLA-B*39:01	18.00						
LTA	387	400	14	EQYMAGVAWLHCLL	HLA-B*39:01	24.00						
LTA	388	395	8	QYMAGVAW	HLA-C*14:02	22.00						
LTA	388	396	9	QYMAGVAWL	HLA-C*14:02	10.00						

LTA	388	399	12	QYMAGVAWLHCL	HLA-A*24:02	43.00	HLA-C*14:02	6.00				
LTA	388	400	13	QYMAGVAWLHCLL	HLA-C*14:02	5.00						
LTA	389	396	8	YMAGVAWL	HLA-A*02:06	9.00	HLA-C*03:03	20.00				
LTA	389	397	9	YMAGVAWLH	HLA-A*29:02	10.00						
LTA	389	399	11	YMAGVAWLHCL	HLA-A*02:01	41.00	HLA-B*39:01	13.00	HLA-C*03:03	28.00		
LTA	389	400	12	YMAGVAWLHCLL	HLA-A*02:01	6.00	HLA-A*02:06	8.00	HLA-B*39:01	13.00	HLA-C*03:03	21.00
LTA	389	402	14	YMAGVAWLHCLLPK	HLA-A*03:01	32.00	HLA-A*11:01	22.00				
LTA	392	402	11	GVAWLHCLLPK	HLA-A*11:01	48.00						
LTA	393	400	8	VAWLHCLL	HLA-C*03:03	21.00						
LTA	395	406	12	WLHCLLPKMDSV	HLA-A*02:01	21.00	HLA-A*02:06	16.00				
LTA	398	406	9	CLLPKMDSV	HLA-A*02:01	40.00	HLA-A*02:06	11.00				
LTA	398	411	14	CLLPKMDSVIFDFL	HLA-A*02:06	34.00						
LTA	400	408	9	LPKMDSVIF	HLA-B*35:01	15.00						
LTA	400	410	11	LPKMDSVIFDF	HLA-B*35:01	34.00						
LTA	402	410	9	KMDSVIFDF	HLA-A*32:01	48.00						
LTA	402	411	10	KMDSVIFDFL	HLA-A*02:01	32.00	HLA-A*02:06	45.00				
LTA	402	414	13	KMDSVIFDFLHCI	HLA-A*02:01	7.00	HLA-A*02:06	18.00				
LTA	402	415	14	KMDSVIFDFLHCIV	HLA-A*02:01	13.00	HLA-A*02:06	18.00				
LTA	405	415	11	SVIFDFLHCIV	HLA-A*68:02	37.00						
LTA	405	416	12	SVIFDFLHCIVF	HLA-B*15:01	38.00						
LTA	405	418	14	SVIFDFLHCIVFNV	HLA-A*02:01	25.00	HLA-A*02:06	6.00	HLA-A*68:02	7.00		
LTA	406	414	9	VIFDFLHCI	HLA-A*02:01	9.00	HLA-A*02:06	14.00	HLA-C*12:03	24.00		
LTA	406	415	10	VIFDFLHCIV	HLA-A*02:06	35.00	HLA-C*12:03	39.00				
LTA	406	418	13	VIFDFLHCIVFNV	HLA-A*02:01	3.00	HLA-A*02:06	6.00	HLA-A*68:02	23.00		
LTA	408	418	11	FDFLHCIVFNV	HLA-B*40:02	41.00						
LTA	409	420	12	DFLHCIVFNVPK	HLA-A*33:01	15.00						
LTA	409	421	13	DFLHCIVFNVPKR	HLA-A*33:01	16.00						
LTA	409	422	14	DFLHCIVFNVPKRR	HLA-A*33:01	7.00						
LTA	410	418	9	FLHCIVFNV	HLA-A*02:01	4.00	HLA-A*02:06	8.00	HLA-A*68:02	28.00		
LTA	410	421	12	FLHCIVFNVPKR	HLA-A*68:01	43.00						
LTA	412	420	9	HCIVFNVPK	HLA-A*30:01	46.00						

LTA	413	420	8	CIVFNVPK	HLA-A*11:01	40.00							
LTA	413	421	9	CIVFNVPKR	HLA-A*68:01	39.00							
LTA	414	421	8	IVFNVPKR	HLA-A*31:01	27.00	HLA-A*68:01	13.00					
LTA	414	422	9	IVFNVPKRR	HLA-A*31:01	33.00	HLA-A*68:01	28.00					
LTA	414	424	11	IVFNVPKRRYW	HLA-B*58:01	31.00							
LTA	414	426	13	IVFNVPKRRYWLF	HLA-A*23:01	42.00							
LTA	414	427	14	IVFNVPKRRYWLFK	HLA-A*03:01	26.00	HLA-A*11:01	14.00	HLA-A*30:01	31.00	HLA-A*68:01	17.00	
LTA	418	426	9	VPKRRYWLF	HLA-B*08:01	17.00							
LTA	419	427	9	PKRRYWLFK	HLA-A*30:01	50.00							
LTA	420	427	8	KRRYWLFK	HLA-A*30:01	8.00							
LTA	421	428	8	RRYWLFKG	HLA-B*27:05	47.00							
LTA	421	430	10	RRYWLFKGPI	HLA-B*27:05	10.00							
LTA	421	434	14	RRYWLFKGPIDSGK	HLA-B*27:05	17.00							
LTA	422	430	9	RYWLFKGPI	HLA-C*14:02	10.00							
LTA	422	434	13	RYWLFKGPIDSGK	HLA-A*31:01	21.00							
LTA	423	430	8	YWLFKGPI	HLA-C*14:02	18.00							
LTA	424	437	14	WLFKGPIDSGKTTL	HLA-A*02:01	50.00							
LTA	428	437	10	GPIDSGKTTL	HLA-B*07:02	43.00	HLA-C*03:03	34.00					
LTA	435	448	14	TTLAAGLLDLCGGK	HLA-A*11:01	33.00							
LTA	437	444	8	LAAGLLDL	HLA-C*03:03	5.00							
LTA	437	450	14	LAAGLLDLCGGKAL	HLA-C*03:03	3.00							
LTA	440	452	13	GLLDLCGGKALNV	HLA-A*02:01	14.00	HLA-A*02:06	23.00					
LTA	441	450	10	LLDLCGGKAL	HLA-C*05:01	50.00	HLA-C*08:02	46.00					
LTA	448	456	9	KALNVNLPM	HLA-B*58:01	18.00	HLA-C*03:03	18.00					
LTA	448	458	11	KALNVNLPMER	HLA-A*31:01	19.00							
LTA	448	459	12	KALNVNLPMERL	HLA-C*03:03	40.00							
LTA	448	461	14	KALNVNLPMERLTF	HLA-A*32:01	27.00	HLA-B*58:01	11.00					
LTA	451	458	8	NVNLPMER	HLA-A*68:01	31.00							
LTA	451	463	13	NVNLPMERLTFEL	HLA-A*68:02	27.00							
LTA	454	461	8	LPMERLTF	HLA-B*35:01	7.00	HLA-B*53:01	24.00					
LTA	454	463	10	LPMERLTFEL	HLA-B*07:02	39.00							

LTA	454	467	14	LPMERLTFELGVAI	HLA-B*07:02	16.00	HLA-B*35:01	43.00	HLA-C*12:03	19.00		
LTA	456	463	8	MERLTFEL	HLA-B*40:02	20.00						
LTA	456	467	12	MERLTFELGVAI	HLA-B*40:02	27.00						
LTA	459	467	9	LTFELGVAI	HLA-A*02:06	40.00	HLA-A*32:01	18.00	HLA-A*68:02	29.00	HLA-C*12:03	19.00
LTA	459	470	12	LTFELGVAIDQY	HLA-A*29:02	31.00						
LTA	459	472	14	LTFELGVAIDQYMV	HLA-A*02:01	43.00	HLA-A*02:06	24.00	HLA-A*68:02	12.00	HLA-C*12:03	48.00
LTA	460	467	8	TFELGVAI	HLA-C*14:02	48.00						
LTA	461	471	11	FELGVAIDQYM	HLA-B*40:01	30.00	HLA-C*03:03	17.00				
LTA	461	472	12	FELGVAIDQYMV	HLA-B*40:02	24.00						
LTA	461	473	13	FELGVAIDQYMVV	HLA-B*40:01	22.00	HLA-B*40:02	32.00				
LTA	461	474	14	FELGVAIDQYMVVF	HLA-B*18:01	5.00	HLA-B*40:01	15.00	HLA-B*40:02	35.00		
LTA	465	472	8	VAIDQYMV	HLA-C*03:03	11.00	HLA-C*12:03	19.00				
LTA	465	473	9	VAIDQYMVV	HLA-C*03:03	4.00	HLA-C*12:03	10.00				
LTA	465	474	10	VAIDQYMVVF	HLA-C*03:03	5.00	HLA-C*12:03	33.00				
LTA	465	477	13	VAIDQYMVVFEDV	HLA-C*03:03	8.00						
LTA	466	474	9	AIDQYMVVF	HLA-C*05:01	34.00						
LTA	469	477	9	QYMVVFEDV	HLA-C*14:02	19.00						
LTA	470	477	8	YMVVFEDV	HLA-A*02:06	14.00						
LTA	471	478	8	MVVFEDVK	HLA-A*68:01	11.00						
LTA	472	485	14	VVFEDVKGTGAESK	HLA-A*11:01	42.00						
LTA	482	493	12	AESKDLPSGHGI	HLA-B*40:01	31.00						
LTA	487	499	13	LPSGHGINNLDSL	HLA-B*07:02	22.00	HLA-B*35:01	42.00				
LTA	492	500	9	GINNLDSLR	HLA-A*31:01	33.00						
LTA	498	509	12	SLRDYLDGSVKV	HLA-A*02:01	41.00						
LTA	502	509	8	YLDGSVKV	HLA-A*02:06	15.00	HLA-C*05:01	48.00	HLA-C*12:03	45.00		
LTA	502	511	10	YLDGSVKVNL	HLA-A*02:01	32.00	HLA-C*05:01	28.00				
LTA	505	513	9	GSVKVNLEK	HLA-A*11:01	28.00						
LTA	505	518	14	GSVKVNLEKKHLNK	HLA-A*11:01	26.00						
LTA	506	513	8	SVKVNLEK	HLA-A*30:01	10.00						
LTA	506	518	13	SVKVNLEKKHLNK	HLA-A*11:01	47.00	HLA-A*30:01	14.00				
LTA	506	519	14	SVKVNLEKKHLNKR	HLA-A*31:01	29.00						

LTA	508	518	11	KVNLEKKHLNK	HLA-A*30:01	27.00				
LTA	508	519	12	KVNLEKKHLNKR	HLA-A*31:01	26.00				
LTA	518	527	10	KRTQIFPPGL	HLA-B*27:05	26.00				
LTA	521	528	8	QIFPPGLV	HLA-A*68:02	26.00	HLA-C*12:03	47.00		
LTA	521	530	10	QIFPPGLVTM	HLA-C*12:03	17.00				
LTA	521	533	13	QIFPPGLVTMNEY	HLA-A*29:02	24.00				
LTA	522	530	9	IFPPGLVTM	HLA-C*14:02	24.00				
LTA	522	535	14	IFPPGLVTMNEYPV	HLA-C*14:02	28.00				
LTA	523	530	8	FPPGLVTM	HLA-B*35:01	8.00				
LTA	523	533	11	FPPGLVTMNEY	HLA-B*35:01	7.00				
LTA	526	535	10	GLVTMNEYPV	HLA-A*02:01	20.00				
LTA	527	535	9	LVTMNEYPV	HLA-A*68:02	27.00				
LTA	528	535	8	VTMNEYPV	HLA-A*02:06	15.00	HLA-A*68:02	23.00		
LTA	528	537	10	VTMNEYPVPK	HLA-A*03:01	43.00	HLA-A*11:01	12.00		
LTA	528	539	12	VTMNEYPVPKTL	HLA-C*03:03	18.00				
LTA	529	537	9	TMNEYPVPK	HLA-A*11:01	17.00	HLA-A*31:01	39.00		
LTA	529	542	14	TMNEYPVPKTLQAR	HLA-A*31:01	16.00				
LTA	531	539	9	NEYPVPKTL	HLA-B*18:01	12.00	HLA-C*12:03	23.00		
LTA	531	542	12	NEYPVPKTLQAR	HLA-B*18:01	39.00				
LTA	531	543	13	NEYPVPKTLQARF	HLA-B*18:01	8.00				
LTA	532	539	8	EYPVPKTL	HLA-C*14:02	14.00				
LTA	532	545	14	EYPVPKTLQARFVR	HLA-A*33:01	43.00				
LTA	533	543	11	YPVPKTLQARF	HLA-B*35:01	11.00				
LTA	533	544	12	YPVPKTLQARFV	HLA-C*12:03	14.00				
LTA	537	544	8	KTLQARFV	HLA-A*02:06	44.00	HLA-A*30:01	29.00	HLA-C*15:02	28.00
LTA	537	545	9	KTLQARFVR	HLA-A*31:01	8.00				
LTA	537	547	11	KTLQARFVRQI	HLA-A*32:01	32.00				
LTA	537	549	13	KTLQARFVRQIDF	HLA-A*32:01	37.00				
LTA	537	550	14	KTLQARFVRQIDFR	HLA-A*31:01	11.00				
LTA	538	545	8	TLQARFVR	HLA-A*31:01	19.00				
LTA	538	550	13	TLQARFVRQIDFR	HLA-A*31:01	26.00				

LTA	539	549	11	LQARFVRQIDF	HLA-B*15:01	45.00				
LTA	539	550	12	LQARFVRQIDFR	HLA-A*31:01	44.00				
LTA	540	550	11	QARFVRQIDFR	HLA-A*31:01	45.00	HLA-A*33:01	35.00	HLA-A*68:01	47.00
LTA	540	552	13	QARFVRQIDFRPK	HLA-A*30:01	10.00				
LTA	541	552	12	ARFVRQIDFRPK	HLA-B*27:05	44.00				
LTA	542	550	9	RFVRQIDFR	HLA-A*31:01	12.00				
LTA	543	550	8	FVRQIDFR	HLA-A*33:01	33.00	HLA-A*68:01	16.00		
LTA	543	556	14	FVRQIDFRPKIYLR	HLA-A*31:01	23.00	HLA-A*33:01	13.00	HLA-A*68:01	17.00
LTA	545	552	8	RQIDFRPK	HLA-A*11:01	26.00	HLA-A*30:01	26.00	HLA-A*31:01	46.00
LTA	545	553	9	RQIDFRPKI	HLA-A*02:06	42.00				
LTA	545	554	10	RQIDFRPKIY	HLA-B*15:01	16.00				
LTA	545	556	12	RQIDFRPKIYLR	HLA-A*31:01	5.00				
LTA	545	557	13	RQIDFRPKIYLRK	HLA-A*11:01	19.00	HLA-A*30:01	34.00		
LTA	548	556	9	DFRPKIYLR	HLA-A*33:01	3.00				
LTA	550	559	10	RPKIYLRKSL	HLA-B*07:02	10.00				
LTA	552	559	8	KIYLRKSL	HLA-C*14:02	22.00				
LTA	552	565	14	KIYLRKSLQNSEFL	HLA-C*14:02	48.00				
LTA	553	564	12	IYLRKSLQNSEF	HLA-A*23:01	31.00	HLA-A*24:02	27.00	HLA-C*14:02	10.00
LTA	553	565	13	IYLRKSLQNSEFL	HLA-A*24:02	30.00	HLA-C*14:02	7.00		
LTA	553	566	14	IYLRKSLQNSEFLL	HLA-A*23:01	45.00	HLA-A*24:02	41.00	HLA-C*14:02	20.00
LTA	554	564	11	YLRKSLQNSEF	HLA-B*15:01	30.00				
LTA	557	564	8	KSLQNSEF	HLA-B*58:01	19.00				
LTA	557	565	9	KSLQNSEFL	HLA-B*58:01	43.00	HLA-C*15:02	19.00		
LTA	557	566	10	KSLQNSEFLL	HLA-C*15:02	44.00				
LTA	557	568	12	KSLQNSEFLLEK	HLA-A*11:01	29.00	HLA-A*30:01	23.00		
LTA	557	570	14	KSLQNSEFLLEKRI	HLA-C*15:02	43.00				
LTA	558	566	9	SLQNSEFLL	HLA-A*02:01	23.00				
LTA	559	566	8	LQNSEFLL	HLA-A*02:06	42.00				
LTA	559	568	10	LQNSEFLLEK	HLA-A*11:01	47.00				
LTA	562	575	14	SEFLLEKRILQSGM	HLA-B*40:02	39.00				
LTA	564	571	8	FLLEKRIL	HLA-C*03:03	14.00				

LTA	564	575	12	FLLEKRILQSGM	HLA-A*02:01	28.00										
LTA	564	577	14	FLLEKRILQSGMTL	HLA-A*02:01	9.00	HLA-A*02:06	12.00	HLA-C*03:03	10.00	HLA-C*12:03	44.00	HLA-C*14:02	30.00		
LTA	566	577	12	LEKRILQSGMTL	HLA-B*40:01	39.00										
LTA	568	577	10	KRILQSGMTL	HLA-B*27:05	44.00										
LTA	568	579	12	KRILQSGMTLLL	HLA-B*27:05	42.00										
LTA	568	580	13	KRILQSGMTLLLL	HLA-B*27:05	40.00										
LTA	568	581	14	KRILQSGMTLLLLL	HLA-B*27:05	35.00										
LTA	570	578	9	ILQSGMTLL	HLA-A*02:01	35.00										
LTA	570	581	12	ILQSGMTLLLLL	HLA-A*02:01	32.00										
LTA	571	578	8	LQSGMTLL	HLA-A*02:06	38.00										
LTA	571	579	9	LQSGMTLLL	HLA-B*39:01	31.00										
LTA	571	580	10	LQSGMTLLLL	HLA-A*02:06	39.00	HLA-B*15:01	44.00								
LTA	571	582	12	LQSGMTLLLLLI	HLA-A*02:06	35.00										
LTA	571	584	14	LQSGMTLLLLLIWF	HLA-B*15:01	43.00										
LTA	572	583	12	QSGMTLLLLLIW	HLA-B*58:01	11.00										
LTA	572	585	14	QSGMTLLLLLIWFR	HLA-A*33:01	40.00										
LTA	573	583	11	SGMTLLLLLIW	HLA-B*58:01	14.00										
LTA	573	585	13	SGMTLLLLLIWFR	HLA-A*31:01	34.00										
LTA	574	585	12	GMTLLLLLIWFR	HLA-A*31:01	16.00	HLA-A*33:01	27.00								
LTA	575	583	9	MTLLLLLIW	HLA-B*58:01	5.00										
LTA	575	585	11	MTLLLLLIWFR	HLA-A*31:01	9.00	HLA-A*33:01	3.00	HLA-A*68:01	9.00						
LTA	575	587	13	MTLLLLLIWFRPV	HLA-A*02:06	15.00	HLA-A*68:02	25.00								
LTA	576	585	10	TLLLLLIWFR	HLA-A*31:01	26.00	HLA-A*33:01	11.00								
LTA	576	587	12	TLLLLLIWFRPV	HLA-A*02:01	28.00	HLA-A*02:06	46.00								
LTA	577	585	9	LLLLLIWFR	HLA-A*31:01	15.00	HLA-A*33:01	5.00								
LTA	577	587	11	LLLLLIWFRPV	HLA-A*02:01	38.00										
LTA	578	585	8	LLLLIWFR	HLA-A*31:01	15.00	HLA-A*33:01	7.00								
LTA	578	587	10	LLLLIWFRPV	HLA-A*02:01	25.00	HLA-A*02:06	20.00								
LTA	579	587	9	LLLIWFRPV	HLA-A*02:01	16.00	HLA-A*02:06	16.00								
LTA	580	587	8	LLIWFRPV	HLA-A*02:06	11.00										
LTA	580	591	12	LLIWFRPVADFA	HLA-A*02:06	47.00										

LTA	587	594	8	VADFATDI	HLA-C*05:01	6.00								
LTA	587	598	12	VADFATDIQSRI	HLA-C*05:01	4.00								
LTA	587	599	13	VADFATDIQSRIV	HLA-C*05:01	6.00								
LTA	590	597	8	FATDIQSR	HLA-A*68:01	15.00								
LTA	590	598	9	FATDIQSRI	HLA-A*68:02	31.00	HLA-C*03:03	5.00	HLA-C*12:03	6.00	HLA-C*12:03	6.00		
LTA	590	599	10	FATDIQSRIV	HLA-C*03:03	6.00								
LTA	590	601	12	FATDIQSRIVEW	HLA-B*53:01	5.00	HLA-B*58:01	19.00						
LTA	590	602	13	FATDIQSRIVEWK	HLA-A*68:01	13.00								
LTA	591	598	8	ATDIQSRI	HLA-C*05:01	34.00								
LTA	593	604	12	DIQSRIVEWKER	HLA-A*33:01	42.00								
LTA	597	604	8	RIVEWKER	HLA-A*31:01	45.00								
LTA	599	612	14	VEWKERLDSEISMY	HLA-B*18:01	30.00								
LTA	604	612	9	RLDSEISMY	HLA-C*05:01	6.00								
LTA	604	614	11	RLDSEISMYTF	HLA-C*05:01	6.00								
LTA	604	617	14	RLDSEISMYTFSRM	HLA-C*05:01	28.00								
LTA	606	616	11	DSEISMYTFSR	HLA-A*68:01	30.00								
LTA	607	614	8	SEISMYTF	HLA-B*40:01	43.00	HLA-B*40:02	45.00	HLA-B*44:02	33.00	HLA-B*44:03	11.00	HLA-B*44:03	11.00
LTA	607	617	11	SEISMYTFSRM	HLA-B*44:03	28.00	HLA-B*44:03	28.00						
LTA	607	619	13	SEISMYTFSRMKY	HLA-B*18:01	19.00	HLA-B*44:02	26.00	HLA-B*44:03	8.00	HLA-B*44:03	8.00		
LTA	608	616	9	EISMYTFSR	HLA-A*33:01	5.00	HLA-A*68:01	6.00						
LTA	608	618	11	EISMYTFSRMK	HLA-A*68:01	21.00								
LTA	608	621	14	EISMYTFSRMKYNI	HLA-A*68:02	19.00								
LTA	609	616	8	ISMYTFSR	HLA-A*11:01	25.00	HLA-A*31:01	8.00	HLA-A*33:01	18.00	HLA-A*68:01	9.00		
LTA	609	617	9	ISMYTFSRM	HLA-B*58:01	38.00	HLA-C*03:03	10.00						
LTA	609	618	10	ISMYTFSRMK	HLA-A*03:01	19.00	HLA-A*11:01	17.00						
LTA	609	619	11	ISMYTFSRMKY	HLA-B*58:01	24.00								
LTA	609	621	13	ISMYTFSRMKYNI	HLA-B*58:01	50.00								
LTA	610	617	8	SMYTFSRM	HLA-C*14:02	20.00								
LTA	610	618	9	SMYTFSRMK	HLA-A*03:01	14.00	HLA-A*11:01	13.00						
LTA	610	619	10	SMYTFSRMKY	HLA-A*03:01	18.00	HLA-A*11:01	41.00	HLA-A*29:02	17.00	HLA-B*15:01	19.00		
LTA	610	621	12	SMYTFSRMKYNI	HLA-A*02:01	37.00	HLA-A*32:01	25.00						

LTA	610	623	14	SMYTFSRMKYNICM	HLA-C*14:02	23.00								
LTA	611	619	9	MYTFSRMKY	HLA-A*29:02	16.00	HLA-C*14:02	26.00						
LTA	611	621	11	MYTFSRMKYNI	HLA-A*23:01	24.00	HLA-C*14:02	26.00						
LTA	611	623	13	MYTFSRMKYNICM	HLA-C*14:02	10.00								
LTA	612	619	8	YTFSRMKY	HLA-A*25:01	43.00	HLA-A*26:01	15.00	HLA-A*29:02	4.00	HLA-A*68:01	33.00	HLA-A*80:01	46.00
LTA	612	621	10	YTFSRMKYNI	HLA-A*68:02	31.00	HLA-C*12:03	14.00						
LTA	612	623	12	YTFSRMKYNICM	HLA-C*03:03	50.00	HLA-C*12:03	25.00	HLA-C*14:02	42.00				
LTA	612	625	14	YTFSRMKYNICMGK	HLA-A*03:01	29.00	HLA-A*11:01	17.00	HLA-A*68:01	5.00				
LTA	615	623	9	SRMKYNICM	HLA-B*27:05	47.00	HLA-B*39:01	25.00	HLA-C*06:02	28.00	HLA-C*07:01	19.00		
LTA	615	627	13	SRMKYNICMGKCI	HLA-C*07:01	36.00								
LTA	615	628	14	SRMKYNICMGKCIL	HLA-B*39:01	10.00	HLA-C*07:01	32.00						
LTA	616	623	8	RMKYNICM	HLA-A*30:01	39.00								
LTA	616	625	10	RMKYNICMGK	HLA-A*03:01	14.00	HLA-A*31:01	18.00						
LTA	616	628	13	RMKYNICMGKCIL	HLA-A*30:01	47.00								
LTA	617	625	9	MKYNICMGK	HLA-A*68:01	48.00								
LTA	618	625	8	KYNICMGK	HLA-A*30:01	44.00	HLA-A*31:01	37.00						
LTA	619	628	10	YNICMGKCIL	HLA-C*03:03	35.00								
LTA	620	632	13	NICMGKCILDITR	HLA-A*33:01	50.00								
LTA	621	628	8	ICMGKCIL	HLA-C*03:03	14.00								
LTA	649	657	9	SQSQCSSQV	HLA-A*02:06	48.00								
LTA	655	662	8	SQVSDTSA	HLA-A*02:06	33.00								
LTA	655	664	10	SQVSDTSAPA	HLA-A*02:06	33.00								
LTA	656	664	9	QVSDTSAPA	HLA-A*68:02	17.00								
LTA	656	669	14	QVSDTSAPAEDSQR	HLA-A*68:01	17.00								
LTA	659	669	11	DTSAPAEDSQR	HLA-A*68:01	11.00								
LTA	660	669	10	TSAPAEDSQR	HLA-A*68:01	20.00								
LTA	668	677	10	QRSDPHSQEL	HLA-C*06:02	24.00	HLA-C*07:01	15.00						
LTA	668	679	12	QRSDPHSQELHL	HLA-C*07:01	46.00								
LTA	669	677	9	RSDPHSQEL	HLA-C*05:01	6.00	HLA-C*15:02	15.00						
LTA	669	679	11	RSDPHSQELHL	HLA-C*05:01	5.00	HLA-C*15:02	47.00						
LTA	669	681	13	RSDPHSQELHLCK	HLA-A*11:01	33.00								

LTA	673	681	9	HSQELHLCK	HLA-A*11:01	34.00						
LTA	676	688	13	ELHLCKGFQCFKR	HLA-A*33:01	30.00						
LTA	678	686	9	HLCKGFQCF	HLA-B*15:01	42.00						
LTA	678	687	10	HLCKGFQCFK	HLA-A*03:01	29.00						
LTA	678	688	11	HLCKGFQCFKR	HLA-A*31:01	35.00	HLA-A*33:01	44.00				
LTA	679	687	9	LCKGFQCFK	HLA-A*30:01	24.00						
LTA	681	688	8	KGFQCFKR	HLA-A*31:01	22.00						
LTA	685	695	11	CFKRPKTPPPK	HLA-A*30:01	34.00						
stA	6	13	8	NREESMEL	HLA-C*07:01	33.00						
stA	6	14	9	NREESMELM	HLA-C*07:01	30.00						
stA	6	17	12	NREESMELMDLL	HLA-C*07:01	49.00						
stA	7	14	8	REESMELM	HLA-B*40:01	28.00						
stA	7	17	11	REESMELMDLL	HLA-B*40:01	7.00						
stA	7	19	13	REESMELMDLLGL	HLA-B*40:01	7.00						
stA	9	17	9	ESMELMDLL	HLA-A*68:02	7.00						
stA	9	19	11	ESMELMDLLGL	HLA-A*68:02	23.00						
stA	9	21	13	ESMELMDLLGLER	HLA-A*33:01	35.00	HLA-A*68:01	18.00				
stA	9	22	14	ESMELMDLLGLERA	HLA-A*68:02	26.00						
stA	11	19	9	MELMDLLGL	HLA-B*40:01	20.00	HLA-B*40:02	28.00				
stA	11	24	14	MELMDLLGLERAAW	HLA-B*18:01	24.00	HLA-B*44:02	18.00	HLA-B*44:03	22.00	HLA-B*44:03	22.00
stA	12	21	10	ELMDLLGLER	HLA-A*33:01	48.00	HLA-A*68:01	34.00				
stA	19	29	11	LERAAWGNLPL	HLA-B*40:01	28.00	HLA-B*40:02	40.00				
stA	20	29	10	ERAAWGNLPL	HLA-B*39:01	7.00						
stA	21	29	9	RAAWGNLPL	HLA-B*07:02	10.00	HLA-C*03:03	6.00				
stA	21	30	10	RAAWGNLPLM	HLA-C*03:03	38.00						
stA	21	31	11	RAAWGNLPLMR	HLA-A*31:01	37.00						
stA	21	32	12	RAAWGNLPLMRK	HLA-A*03:01	43.00	HLA-A*11:01	25.00				
stA	21	34	14	RAAWGNLPLMRKAY	HLA-B*35:01	26.00						
stA	22	29	8	AAWGNLPL	HLA-C*03:03	7.00						
stA	22	30	9	AAWGNLPLM	HLA-C*03:03	41.00						
stA	22	34	13	AAWGNLPLMRKAY	HLA-B*35:01	44.00						

stA	22	35	14	AAWGNLPLMRKAYL	HLA-C*03:03	10.00				
stA	27	34	8	LPLMRKAY	HLA-B*35:01	8.00				
stA	27	35	9	LPLMRKAYL	HLA-B*07:02	35.00	HLA-B*08:01	13.00		
stA	29	36	8	LMRKAYLR	HLA-A*31:01	13.00				
stA	29	37	9	LMRKAYLRK	HLA-A*03:01	27.00	HLA-A*30:01	7.00		
stA	29	39	11	LMRKAYLRKCK	HLA-A*30:01	19.00				
stA	29	41	13	LMRKAYLRKCKEF	HLA-B*15:01	30.00				
stA	32	39	8	KAYLRKCK	HLA-A*30:01	50.00				
stA	32	45	14	KAYLRKCKEFHPDK	HLA-A*03:01	45.00	HLA-A*30:01	25.00		
stA	33	41	9	AYLRKCKEF	HLA-C*14:02	13.00				
stA	37	45	9	KCKEFHPDK	HLA-A*30:01	6.00				
stA	41	52	12	FHPDKGGDEDKM	HLA-C*07:01	31.00	HLA-C*14:02	45.00		
stA	48	59	12	DEDKMKRMNTLY	HLA-B*18:01	14.00				
stA	51	58	8	KMKRMNTL	HLA-A*30:01	26.00				
stA	51	59	9	KMKRMNTLY	HLA-A*30:01	33.00	HLA-A*30:02	33.00	HLA-A*80:01	46.00
stA	51	60	10	KMKRMNTLYK	HLA-A*03:01	14.00	HLA-A*11:01	28.00	HLA-A*31:01	16.00
stA	51	61	11	KMKRMNTLYKK	HLA-A*30:01	5.00	HLA-A*31:01	8.00		
stA	52	60	9	MKRMNTLYK	HLA-A*30:01	7.00				
stA	53	61	9	KRMNTLYKK	HLA-B*27:05	17.00				
stA	53	62	10	KRMNTLYKKM	HLA-B*27:05	28.00				
stA	54	61	8	RMNTLYKK	HLA-A*03:01	40.00	HLA-A*11:01	26.00	HLA-A*31:01	10.00
stA	62	70	9	MEQDVKVAH	HLA-B*18:01	41.00				
stA	62	74	13	MEQDVKVAHQPDF	HLA-B*18:01	19.00	HLA-B*44:02	40.00		
stA	67	77	11	KVAHQPDFGTW	HLA-B*58:01	12.00				
stA	70	82	13	HQPDFGTWSSSEV	HLA-A*02:06	23.00				
stA	77	88	12	WSSSEVCADFPL	HLA-C*03:03	26.00				
stA	78	88	11	SSSEVCADFPL	HLA-A*68:02	38.00				
stA	80	88	9	SEVCADFPL	HLA-B*40:01	3.00				
stA	80	93	14	SEVCADFPLCPDTL	HLA-B*40:01	7.00				
stA	81	88	8	EVCADFPL	HLA-A*68:02	22.00				
stA	83	94	12	CADFPLCPDTLY	HLA-A*01:01	29.00	HLA-C*05:01	11.00		

stA	86	93	8	FPLCPDTL	HLA-B*35:01	14.00	HLA-B*53:01	49.00	HLA-C*03:03	28.00	HLA-C*12:03	23.00
stA	86	94	9	FPLCPDTLY	HLA-B*35:01	4.00	HLA-B*53:01	18.00				
stA	86	98	13	FPLCPDTLYCKEW	HLA-B*53:01	15.00						
stA	89	98	10	CPDTLYCKEW	HLA-B*53:01	34.00						
stA	91	104	14	DTLYCKEWPICSKK	HLA-A*68:01	35.00						
stA	92	100	9	TLYCKEWPI	HLA-A*02:01	10.00	HLA-A*02:06	39.00				
stA	92	103	12	TLYCKEWPICSK	HLA-A*03:01	29.00	HLA-A*11:01	37.00				
stA	92	104	13	TLYCKEWPICSKK	HLA-A*03:01	23.00	HLA-A*11:01	45.00				
stA	94	107	14	YCKEWPICSKKPSV	HLA-C*12:03	41.00						
stA	96	107	12	KEWPICSKKPSV	HLA-B*40:02	24.00						
stA	106	117	12	SVHCPCMLCQLR	HLA-A*68:01	31.00						
stA	107	118	12	VHCPCMLCQLRL	HLA-C*07:01	28.00						
stA	112	119	8	MLCQLRLR	HLA-A*33:01	34.00						
stA	112	123	12	MLCQLRLRHLNR	HLA-A*31:01	40.00	HLA-A*33:01	21.00				
stA	114	123	10	CQLRLRHLNR	HLA-A*31:01	47.00						
stA	114	127	14	CQLRLRHLNRKFLR	HLA-A*31:01	12.00						
stA	115	123	9	QLRLRHLNR	HLA-A*33:01	34.00						
stA	115	126	12	QLRLRHLNRKFL	HLA-B*08:01	31.00						
stA	115	127	13	QLRLRHLNRKFLR	HLA-A*31:01	16.00	HLA-A*33:01	17.00				
stA	115	128	14	QLRLRHLNRKFLRK	HLA-A*03:01	35.00	HLA-A*30:01	35.00				
stA	117	124	8	RLRHLNRK	HLA-A*03:01	28.00	HLA-A*30:01	6.00				
stA	117	127	11	RLRHLNRKFLR	HLA-A*31:01	5.00						
stA	117	128	12	RLRHLNRKFLRK	HLA-A*03:01	24.00	HLA-A*30:01	4.00				
stA	119	127	9	RHLNRKFLR	HLA-A*31:01	9.00						
stA	119	132	14	RHLNRKFLRKEPLV	HLA-C*07:01	45.00						
stA	120	127	8	HLNRKFLR	HLA-A*31:01	6.00	HLA-A*33:01	20.00				
stA	120	128	9	HLNRKFLRK	HLA-A*03:01	18.00	HLA-A*11:01	41.00				
stA	120	131	12	HLNRKFLRKEPL	HLA-B*08:01	37.00						
stA	124	131	8	KFLRKEPL	HLA-C*14:02	18.00						
stA	129	137	9	EPLVWIDCY	HLA-B*35:01	23.00						
stA	129	142	14	EPLVWIDCYCIDCF	HLA-B*35:01	45.00						

stA	132	142	11	VWIDCYCIDCF	HLA-A*23:01	31.00				
stA	133	142	10	WIDCYCIDCF	HLA-C*05:01	28.00				
stA	133	146	14	WIDCYCIDCFTQWF	HLA-C*05:01	25.00				
stA	137	148	12	YCIDCFTQWFGL	HLA-C*03:03	10.00				
stA	137	150	14	YCIDCFTQWFGLDL	HLA-C*03:03	6.00				
stA	144	155	12	QWFGLDLTEETL	HLA-C*14:02	31.00				
stA	146	155	10	FGLDLTEETL	HLA-C*03:03	15.00				
stA	146	157	12	FGLDLTEETLQW	HLA-B*58:01	21.00				
stA	146	159	14	FGLDLTEETLQWWV	HLA-A*02:06	24.00				
stA	147	159	13	GLDLTEETLQWWV	HLA-A*02:01	29.00				
stA	150	157	8	LTEETLQW	HLA-B*58:01	25.00				
stA	150	159	10	LTEETLQWWV	HLA-A*02:06	40.00				
stA	151	158	8	TEETLQWW	HLA-B*44:02	15.00	HLA-B*44:03	21.00	HLA-B*44:03	21.00
stA	155	162	8	LQWWVQII	HLA-A*02:06	36.00				
stA	155	167	13	LQWWVQIIGETPF	HLA-B*15:01	29.00				
stA	155	168	14	LQWWVQIIGETPFR	HLA-A*31:01	23.00				
stA	158	168	11	WVQIIGETPFR	HLA-A*68:01	43.00				
stA	159	167	9	VQIIGETPF	HLA-B*15:01	23.00				
stA	159	170	12	VQIIGETPFRDL	HLA-C*03:03	32.00				
stA	160	168	9	QIIGETPFR	HLA-A*68:01	19.00				
stA	160	171	12	QIIGETPFRDLK	HLA-A*11:01	46.00				
stA	163	170	8	GETPFRDL	HLA-B*40:01	19.00				
stA	163	172	10	GETPFRDLKL	HLA-B*40:01	19.00				
stA	164	171	8	ETPFRDLK	HLA-A*68:01	23.00				
